# Outcomes of Patients with Positive Interim Positron Emission Tomography (PET) Continuing ABVD in the Clinical Setting

**DOI:** 10.3390/cancers15061760

**Published:** 2023-03-14

**Authors:** Serena Zheng, Kanika Gupta, Piyush Goyal, Reiko Nakajima, Laure Michaud, Connie Lee Batlevi, Paul A. Hamlin, Steven Horwitz, Anita Kumar, Matthew J. Matasar, Alison J. Moskowitz, Craig H. Moskowitz, Ariela Noy, M. Lia Palomba, David J. Straus, Gottfried Von Keudell, Lorenzo Falchi, Joachim Yahalom, Andrew D. Zelenetz, Anas Younes, Gilles Salles, Heiko Schöder, Erel Joffe

**Affiliations:** 1 Icahn School of Medicine at Mount Sinai, New York, NY 10029, USA; 2 New York Medical College, Valhalla, NY 10595, USA; 3 College of Osteopathic Medicine, Touro University California, Vallejo, CA 94592, USA; 4 Memorial Sloan Kettering Cancer Center, Department of Radiology, New York, NY 10065, USA; 5 Memorial Sloan Kettering Cancer Center, Department of Medicine, Lymphoma Service, New York, NY 10065, USA; 6 Weill Cornell College of Medicine, New York, NY 10065, USA; 7 Sylvester Cancer Center, University of Miami, Miami, FL 33136, USA; 8 AstraZeneca, 1800 Concord Pike, Wilmington, DE 19803, USA

**Keywords:** PET adapted therapy, ABVD, Hodgkin’s lymphoma

## Abstract

**Simple Summary:**

This study aimed to highlight limitations in the use of interim PET (iPET) for treatment decisions and prognostication in the frontline management of Hodgkin’s lymphoma. It reinforces the observation from the ECHELON-1 study that outcomes of patients with a positive interim PET who nonetheless continue treatment with ABVD are not as dismal as previously described. Furthermore, we describe the performance of PET positivity grading by a quantitative measure based on SUV ratios compared to the subjective Deauville scoring.

**Abstract:**

Recent prospective clinical trial data suggest that patients with Hodgkin’s lymphoma who continue treatment with ABVD, despite failing to attain a complete metabolic response on interim PET (PET2+), may fare better than previously published. We describe the outcomes of PET2+ patients who continued ABVD and compare the performance of a quantitative measure based on the lesion-to-liver SUV ratio (LLS qPET2+) to that of the subjective Deauville criteria (dvPET2+). We analyzed all patients with newly diagnosed advanced-stage Hodgkin lymphoma treated with frontline ABVD at the Memorial Sloan Kettering Cancer Center between 2008 and 2017. Eligibility was set to correspond with the RATHL inclusion criteria. Images were reviewed by two nuclear medicine physicians and discordant cases were resolved with a third expert in consensus. qPET2+ was defined as LLS ≥ 1.3. We identified 227 patients of whom 25% (57) were qPET2+, but only 14% (31) were dvPET2+. Forty-eight patients (84%) continued ABVD with a 3-year PFS of 70% for qPET2+ and 64% for dvPET2+. In conclusion, interim PET interpretation in clinical practice may be associated with a higher rate of scans deemed positive. Irrespective of the criteria for PET2 positivity, a subset of patients may continue ABVD without a dismal outcome.

## 1. Introduction

Hodgkin’s lymphoma (HL) is known for being one of the most curable cancers. However, a subset of patients fare poorly. As a result, two competing treatment paradigms have evolved. The first focuses on maximizing the cure rates by employing the more intensive escalated BEACOPP regimen (bleomycin, etoposide, doxorubicin, cyclophosphamide, vincristine, procarbazine, prednisone) [1,2,3,4]. The second employs a stepwise approach whereby patients are treated with the less efficacious yet less toxic ABVD (doxorubicin, bleomycin, vinblastine, dacarbazine), referring patients with incomplete responses or progression to salvage regimens followed by high-dose chemotherapy with stem cell support (ASCT) [5]. In an attempt to improve upon the stepwise ABVD paradigm, it has been suggested to use positron emission tomography interim scanning after two cycles of treatment (PET2) to identify high risk patients for early treatment intensification without the use of subsequent ASCT [4,6].

Several studies have demonstrated the prognostic power of interim PET for the prediction of treatment failure and early relapse after ABVD [7,8]. The seminal study by Gallamini et al. reported a 13% probability of a 2-year PFS for PET2+ patients compared to 96% for PET2−. PET2 status was thus determined as the single most important prognostic factor in advanced HL [8]. A subsequent international multi-institutional study demonstrated a 3-year failure-free survival of 28% for PET2+ and of 83% for PET2− patients, validating interim PET as a robust prognostic factor [9]. Following these reports, large studies evaluating ABVD have been assigning patients with PET2+ scans to intensified regimens, without an ABVD comparator arm [4,6,10]. However, the recently published post-hoc analysis of the ECHELON-1 trial demonstrated considerably better outcomes for patients with positive PET2 scans who continued on ABVD, with a 3-year PFS of 52% [11,12].

Importantly, in many of the studies demonstrating poor outcomes in PET2+ patients continuing ABVD, the determination of refractory or progressing disease could be made solely based on the presence of residual FDG avidity on the end of treatment scan without mandating a confirmatory biopsy (Supplementary Table S1—selected studies). This was the case in the ECHELON-1 study and may have led to an overestimation of events in the ABVD arm because of non-lymphomatous residual FDG uptake [13]. In fact, previous studies have shown that the false positive rate of PET in HL is not negligible, ranging from 15–24% [14,15,16]. More recently, the measurement of circulating tumor DNA levels in the peripheral blood has been suggested as a method to overcome the false-positive and false-negative rates of PET2 in the prediction of long-term treatment outcomes in HL [17].

It has been our unpublished clinical experience that the disease course of PET2+ patients who continue ABVD is in keeping with that reported on the ECHELON-1 study. Importantly, it has been our practice to define a disease as progressive only after a confirmatory biopsy. In this work, we sought to describe the outcomes of PET2+ patients who continued ABVD and to evaluate the utility of determining PET2−positivity by a quantitative reviewer-independent measure based on standard FDG uptake values (SUV).

## 2. Materials and Methods

**Figure 1:**
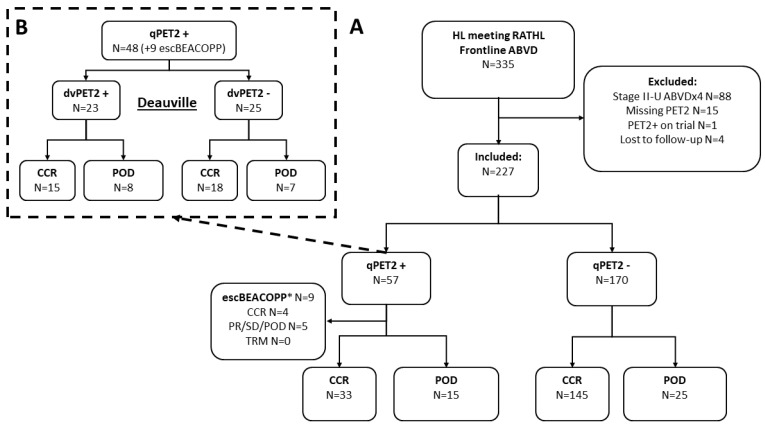
CONSORT diagram. CONSORT diagram and ultimate progression-free-survival events (for CONSORT depicting response to treatment see Supplementary Figure S1). (**A**) Interim PET by semi-quantitative lesion SUVmax to liver SUVmean ratio ≥ 1.3 (qPET); (**B**) interim PET by Deauville criteria. Inclusion criteria were modeled after the RATHL protocol (stage IIB to IV, or stage IIA with bulky disease or at least three involved sites). We excluded patients who only received four cycles of ABVD, did not undergo a PET scan after cycle 2 of ABVD, switched to any treatment other than escBEACOPP, or were lost to follow-up before the completion of therapy. Abbreviations: CCR, clinical complete response; POD, progression of disease; PR, partial response; SD, stable disease; TRM, treatment related mortality. See also Supplementary Figure S1—CONSORT diagram. * Data for patients switched to escBEACOPP are included for reference only.

In this retrospective study, we reviewed all patients with newly diagnosed advanced-stage Hodgkin lymphoma treated with a plan for 6 cycles of frontline ABVD at Memorial Sloan Kettering Cancer Center between 2008 and 2017. Eligibility criteria were set to correspond with the RATHL inclusion (stage IIB to IV, or II with bulky disease or ≥3 involved sites) [6]. We excluded patients with PET2−positivity who were switched to treatment other than escBEACOPP (N = 1), those missing an interim PET scan after 2 cycles of ABVD (N = 15), those who only completed 4 cycles of ABVD for reasons other than progression (e.g., stage II bulky treated as early-unfavorable), and those lost to follow-up before completion of the treatment (Figure 1—CONSORT). The study was approved by the institutional review board and conducted in accordance with the Declaration of Helsinki and the Good Clinical Practice guidelines.

We reviewed all PET2 reports undertaken after 2 cycles of ABVD and identified those in which the staff radiologist indicated a concern for residual uptake above liver. All these cases were subsequently reviewed independently by two experts in nuclear radiology (LM and RN) to confirm the presence of residual uptake and validate the measurements of maximal lesion SUV and mean and maximal SUV in the liver. All PET2+ images were graded by the two nuclear medicine physicians per the Deauville (DV) criteria as follows: no uptake (1), slight uptake lower than the mediastinal blood pool (2), uptake equal to or slightly higher than the mediastinal blood pool but less than liver (3), uptake moderately higher than the liver (4), and uptake markedly higher than the liver or new FDG avid lesions consistent with disease progression (5). dvPET2+ was defined as DV ≥ 4. Cases with a discordant interpretation by the two readers were resolved with a third expert (HS) in consensus.

All images were also graded using a quantitative reviewer-independent scale whereby a qPET2+ was defined as those cases with a lesion SUV_max_ to liver SUV_mean_ ratio ≥ 1.3 per the qPET threshold (noting that the original qPET measure used the SUVpeak rather than SUVmax where SUV_max_ is the highest tracer uptake in a single voxel and SUV_Peak_ is the average uptake in a 1 cm region of interest in the region of highest FDG uptake) [18,19]. For all patients, we calculated the IPS as previously described [20].

Progression-free was calculated from the date of initial treatment until progression of disease or death of any cause, and overall survival (OS) from initial treatment until death of any cause, censoring cases without an event at the date of last clinical follow-up. Consolidative radiation, planned or not, was not considered as a PFS event (Supplementary Figure S1—CONSORT—response and use of radiotherapy). All progressions were confirmed by biopsy. Images and patient specific data including baseline stage, IPS, lesion/liver SUVs, use of radiation, biopsy, site of progression, and salvage regimen for all patients with a positive PET2 are provided in the Supplementary Materials File S1.

### Statistical Analysis

We compared the baseline features between the PET2− and PET2+ patients treated with ABVD using the Fisher exact test for discrete variables and the Wilcoxon rank sum for numeric values. Follow-up time was estimated using the reverse Kaplan–Meier test. Survival was compared between the groups using Kaplan–Meier plots and the log-rank test. As the objective of this study was to evaluate outcomes of the patients who continued on ABVD despite PET2+, the data about nine patients treated with escBEACOPP are presented for reference only (see Discussion regarding the interpretation of results in the context of possible selection bias). All analyses were performed using R version 3.5 (R foundation, Viena, Austria).

## 3. Results

We identified 227 patients meeting the RATHL inclusion criteria treated with ABVD. The median age was 34 (range 18–87), with 25% (57) older than 45 years, 12% (26) with an IPS ≥ 4; 28% (64) stage II (5%, N = 12 stage II–X), and 38% (87) with extranodal involvement (Table 1; Supplementary Table S2). There were 57 (25%) patients who were qPET2+ (lesion SUV_max_ to liver SUV_mean_ ratio ≥ 1.3), dan most continued on ABVD (48, 84%), but nine were switched to escBEACOPP (Figure 1). Compared to patients who remained on ABVD, a greater percentage of the patients who were switched to escBEACOPP had an IPS ≥ 4 (33%) and bulky disease (44%) (Table 1; see Supplementary Materials, File S1). Only 31 of the qPET2+ patients were considered dvPET2+ by the nuclear medicine physicians as per the Deauville criteria (14% of the entire cohort). Of these, 74% (23) continued ABVD and eight were switched to escBEACOPP (one patient with DV-3 was also switched to escBEACOPP) (Figure 1).

**Figure 2:**
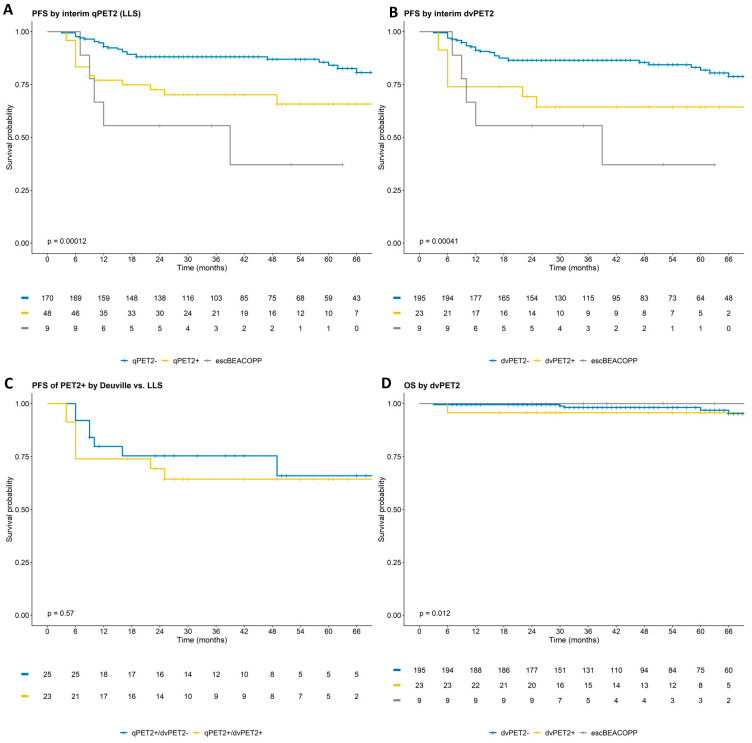
Progression free and overall survival by dvPET2 and qPET2. PFS by qPET2 (**A**); PFS by dvPET2 (**B**); comparison of PFS in patients considered qPET2+ but not dvPET2+ to those considered PET2+ by both assessments (**C**); overall survival by dvPET2 (not depicted 2 events at 6.5 and 8.5 years) (**D**). *p*-values calculated only for patients treated with ABVD. Patients treated with escBEACOPP are presented for reference.

With a median follow-up of 47 months, patients with dvPET2+ who continued ABVD had a 3-year PFS of 64% (95% CI 47–88%), with 20% of all patients who ultimately experienced a PFS event being dvPET2+. Patients with dvPET2− had a 3-year PFS of 86% (95% CI 0.82–0.91) (Figure 2; Table 2).

The 3-year PFS by qPET2 was not statistically different to that by dvPET2 (70% vs. 64%, *p* = 0.6 for PET2+; and 88% vs. 86%, *p* = 0.9 for PET2−). The nine patients who were switched to escBEACOPP had a 3-year PFS of 56% (95% CI 31–100%, eight dvPET+ and one dvPET), which may have translated to a slight overestimation of good outcomes in the PET2+ ABVD subgroups (Figure 2; Table 2). Of note, overall, there were 12 patients with a Deauville score of 5 (SUVmax range 5.5–10.8), of whom seven continued ABVD, none of whom had experienced a POD, and five switched to escBEACOPP with three experiencing a POD.

There were 21 (9%) patients who received consolidative radiation. Of these, 15 were patients in PET CR at the end of therapy (10 previously assessed as qPET2 negative and five as qPET2 positive) and six were due to persistent FDG uptake (two qPET2 positive; four escBEACOPP) (Supplementary Figure S1—CONSORT—response and use of radiotherapy). Overall survival was excellent regardless of PET2 status or PET2 evaluation criteria (5 years—OS 96% for PET2+ and 98% for PET2−). In this regard, most patients with refractory or relapsed disease went on to salvage regimens including novel agents such as brentuximab, followed by ASCT (Supplementary File S1).

**Figure 3:**
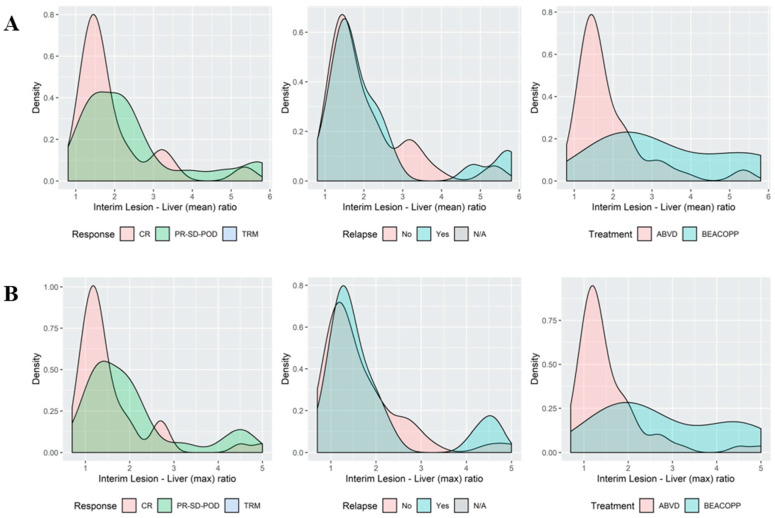
Histograms of the association between the qPET2 thresholds for the LLS ratio and outcomes histogram depicting the occurrence of cases (*Y* axis) at different LLS ratios. The area under the curve represents all cases. Note the overlap in ‘peaks’ at the lower LLS ratio and at the higher LLS ratios. Distribution of the interim lesion SUVmax to liver SUVmean ratio (*X* axis) (**A**) and the lesion SUVmax to liver SUVmax ratio (**B**), demonstrating an overlap between patients with ‘good’ (CR; no relapse) and ‘bad’ (PR-SD-POD; relapse) outcomes. For interim PET images and outcomes see Supplementary File S1.

When reviewing the association between various values of lesion-to-liver SUV ratios and different outcome measures such as response to treatment or occurrence of refractory or relapsed disease, there was no distinct cutoff to discern patients with poor prognosis from those with a delayed, but complete response to continued ABVD. That is, even borderline elevation of SUV values above the liver was associated with poorer outcomes, with a modest tradeoff between sensitivity and specificity (Figure 3; Supplementary File S1).

## 4. Discussion

Recent data from the ECHELON-1 study demonstrated that patients with advanced stage Hodgkin’s who displayed a positive interim PET on ABVD had better outcomes than the previously retrospectively described outcomes (albeit still inferior to those of patients with a negative PET2) [21]. Along the same lines, this study aimed to evaluate the rate and prognostic implication of PET2 positivity in the clinical setting. We identified 227 patients meeting the RATHL inclusion criteria who were planned for six cycles of ABVD. Of these, 25% were suspected to have a positive PET2 (as defined by a lesion SUVmax to liver SUVmean ratio ≥ 1.3; qPET2+), but only 14% were positive by consensus Deauville designation (dvPET2). Most patients with PET2+ continued ABVD with a 3-year PFS of 70% for qPET2+ and 64% for dvPET2+. Notably, POD was uniformly confirmed by biopsy and 5-year OS was very good across groups.

We modeled our study on the design of the RATHL protocol, which is the current standard of PET2 guided treatment with ABVD for advanced HL. Like RATHL and similar studies, 25% of the patients were older than 45 years, over two thirds (72%) had stage III/IV disease, and over one third (38%) had extranodal involvement. The rate of PET2−positivity by the Deauville criteria (14%) was in keeping with previous publications (9–29%) [6,8,9,11,21,22,23].

How can one explain, then, the better outcomes seen in our PET2+ cohort compared to prior reports, in particular to the 3-year PFS of 52% reported for the PET2+ patients on the ABVD control arm of ECHELON-1 [12] First, inclusion of the nine cases transitioned to escBEACOPP (of which at least five would have been expected to experience a PFS event with ABVD as they did on escBEACOPP) would have resulted in a 3-year PFS in the range of 50–60%, in keeping with ECHELON-1. A more profound selection bias is unlikely considering the similar PET2 positivity rates compared to prior studies, and a similar percentage of patients referred to escBEACOPP compared to reports from other U.S. institutions [24]. A possible explanation is that many protocols have used PET, not only as the modality for interim imaging, but also as the sole modality for defining the PFS event of refractory disease referred to further treatment [8,21]. However, it is known that the false positive rate of PET in HL is not negligible, ranging from 15 to 24% [14,15,16]. A good example is the original ECHELON-1 study, which used as its primary endpoint a modified PFS (mPFS) that was included as an event imaging-based designation of incomplete response and subsequent anticancer therapy [21]. The definition of PET progression in that setting was conducted locally and did not require a centralized review of the images. Thus, of the 31 PFS events in the PET2+ cases on the study’s ABVD arm, up to 22 had residual FDG avidity at the end of treatment, were declared treatment failure, and went on to further treatment without requiring a confirmatory biopsy [12,21] Importantly, 18 of these cases were later deemed false-positives by a central review and may not have required further treatment [21]. Thus, it is possible that the 52% 3-year PFS for PET2+ patients on ECHELON-1 was underestimated. Similarly, a recent multi-institutional study analyzing data from 12 U.S. centers documented that few patients had a confirmatory biopsy following a positive PET2 and that over half of these biopsies failed to document active lymphoma [24]. Likewise, an evaluation of PET at the end of therapy with ABVD for identifying a residual disease by a fine needle core biopsy demonstrated that the PPV for Deauville 4 cases was only 13%, while for Deauville 5 cases, it was 100%. Importantly, all patients with a positive PET and a negative biopsy were progression-free after a 5-year follow-up, highlighting the excellent negative predictive value of the biopsies in this setting [25]. In this regard, a preliminary evaluation of circulating tumor DNA (ctDNA) against interim PET in Hodgkin’s lymphoma demonstrated that only two out of six patients with a PET2+ scan eventually progressed, both having less than a 2-log-fold decrease in ctDNA. The remaining four patients with PET2+ had a more profound decrease in ctDNA and never progressed, suggesting ctDNA as an alternative to a confirmatory biopsy in this scenario [17].

We also noted a higher rate of lesions with concerning elevated SUVs (qPET2+) than those eventually deemed dvPET2+ (25% vs. 14% respectively) which may represent a higher rate of suspected PET2+ reads in clinical practice. The Deauville score used to differentiate the threshold for a positive PET2 from a negative one (i.e., DV ≥ 4 as opposed to DV ≤ 3) employs a subjective visual definition of “at least a moderately increased uptake above liver background” [26]. In the clinical setting, it is often unclear what constitutes a “moderately” increased uptake, and there is considerable variability in the rate of positive scans across studies and between local and central review [9]. For example, in the international validation of PET2, 27% of cases deemed PET2+ were overruled and considered negative by central review, as were 6% of cases initially deemed PET2− that were subsequently overruled and considered PET2+ [9]. A similar observation was made in the ECHELON-1 trial, where independent investigators classified more patients as ‘incomplete response’ at the end of treatment compared to the assessment by the independent review committee [21]. It may be that there is a higher rate of cases considered PET2-positive in the clinical setting because the interpretation of the PET scans is influenced by the context in which it is read. For example, a scan may be interpreted more conservatively when it is used for treatment escalation/de-escalation, or when a mistake may be associated with medical liability. Thus, the PET2 positivity rate in the RATHL study, where PET was used to de-escalate treatment, was 16% compared to 9% in the ABVD arm of the ECHELON-1 study, where the PET reading had no implication for treatment decisions [6,12]. Likewise, the rate of PET2+ was 18% on SWOG S8016 and 20% on the HD0801, protocols such as RATHL intensified treatment in PET2+ patients [4,10]. In this regard, a quantitative method of defining increased uptake based on SUV may make the definition of PET response more reproducible in clinical practice [18,19].

Notwithstanding, our data demonstrate that even low levels of FDG avidity (here defined as lesion SUV_max_ to liver SUV_mean_ ratio ≥ 1.3) not meeting the Deauville 4 criteria by visual assessment are associated with a worse prognosis, albeit at a lower specificity. Over a third of the patients with qPET2+ were classified as dvPET2−. These patients had a similar 3-year PFS to qPET2+/dvPET2+ patients and a significantly shorter 3-year PFS than patients considered PET2− by both criteria (Figure 2). Furthermore, when evaluating the distribution of lesion-to-liver SUV ratios across patients with and without a PFS event, there was no clear cutoff to discern between the different prognoses, suggesting that any value of SUV above the liver-mean may be associated with a poorer prognosis (Figure 3). In the rPET study, a positive cutoff of rPET ≥ 1.14, like DV ≥ 4, was found to be a strong outcome predictor in HL, demonstrating similar negative predictive values to the Deauville score and superior sensitivity [27]. Another measure of tumor to liver uptake ratio (rDS) ≥ 1.4 was evaluated in DLBCL and found to be superior to the Deauville score in survival prediction [28]. Whether the qPET2 evaluation is superior to dvPET2 and at what cutoffs depend on the context of the clinical decision. Specifically, whether the objective is to maximize the identification of ‘at-risk’ patients (for example, in the case of an available low-toxicity and/or high-efficacy treatment modification), or for the exclusion of ‘good’ patients (for example, for treatment de-escalation). These explorations would require a much larger sample than available in our study and the incorporation of a validation set. It is possible that to improve the accuracy of the prognostic predication of PET, an independent modality such as circulating tumor DNA should be incorporated [17].

Since the initial publication of a high rate of treatment failure of ABVD in patients with PET2+ scans, several large clinical trials have directed this subset of patients to intensified treatment with escBEACOPP without a comparator arm of ABVD (as the latter was considered ethically unjustifiable). These studies demonstrated a 3-year PFS of 60–70% for escBEACOPP [4,6,21,29]. Unfortunately, this 3-year PFS rate was considerably lower than the excellent overall 3-year PFS of 92% seen in the HD-18 trial for upfront escBEACOPP. This difference highlights that much of the superiority of escBEACOPP over ABVD is, in fact, driven by better PFS outcomes in patients who would have had a negative PET2 on ABVD [1,6]. Thus, while one can clearly make a case to favor frontline escBEACOPP over ABVD for upfront treatment (particularly with the prospect of an abbreviated PET adapted treatment), it remains unclear whether switching PET2+ patients to escBEACOPP is superior to the continuation of ABVD with close monitoring. The latter approach would limit intensive treatment to approximately 40–50% of patients with truly refractory disease while the former would mean 30–40% of the PET2+ patients would require salvage therapy, despite having been switched to escBEACOPP. Unfortunately, whether treated with escBEACOPP or a subsequent salvage, a substantial number of these patients will prove to be refractory to any treatment based solely on chemotherapy, noting a PFS of only 41% of patients undergoing ASCT for relapsed refractory disease on the ATHERA placebo-control arm. Thus, there is a strong case for exploring regimens incorporating novel agents in this patient population, either upon a positive PET2 or at the end of therapy [30,31].

Finally, as with previous studies, though PET2-positivity was clearly associated with a higher rate of treatment failures and early progressions, overall, patients with HL had excellent long-term outcomes regardless of PET2 status. Similar results were demonstrated previously, with an overall survival at 2 years ranging from 88 to 98% and at 5 years ranging from 85 to 95% [32].

## 5. Conclusions

The outcomes of the PET2+ patients who continued ABVD in this analysis were better than previously reported, though still inferior to those reported for patients switched to escBEACOPP in recent clinical trials. Notably, even patients with PET2+ who remained on ABVD had an excellent overall survival, verifying the ability to salvage patients with relapsed or refractory disease.

## Figures and Tables

**Table 1:** Descriptive statistics by PET2 status.

Characteristic	All	qPET2−	qPET2+	EscBEACOPP ^†^	*p* *
No. of patients	227	170	48	9	
Median age (range), years	34 [18; 87]	34 [18; 82]	35.0 [19; 87]	32.0 [19; 59]	0.66
Age ≥ 45	57 (25%)	41 (24%)	13 (27%)	3 (33%)	0.82
Female sex	115 (51%)	87 (51%)	24 (50.0%)	4 (44%)	0.99
ECOG > 1	6 (3%)	3 (2%)	3 (6%)	0 (0%)	0.12
Ann Arbor Stage					0.12
II-U	52 (23%)	39 (23%)	10 (21%)	3 (33%)	
II-X	12 (5%)	6 (4%)	5 (10%)	1 (11%)	
III	83 (37%)	68 (40%)	13 (27%)	2 (22%)	
IV	80 (35%)	57 (34%)	20 (42%)	3 (33%)	
IPS score					0.004
0–3	201 (89%)	158 (93%)	37 (77%)	6 (67%)	
4–7	26 (12%)	12 (7%)	11 (23%)	3 (33%)	
Extranodal Involvement	87 (38%)	61 (36%)	23 (48%)	3 (33%)	0.18
Bulk	29 (13%)	18 (11%)	7 (15%)	4 (44%)	0.61
Response (chemotherapy)					
CR	194 (86%)	159 (94%)	31 (65%)	4 (44%)	<0.001
PR	12 (5%)	1 (1%)	9 (19%)	2 (22%)	
POD	19 (8%)	9 (5%)	7 (15%)	3 (33%)	
TRM	2 (1%)	1 (1%)	1 (2%)	0 (0%)	
Radiotherapy	21 (9%)	10 (6%)	7 (15%)	4 (44%)	0.07

Descriptive statistics grouped by dvPET are presented in Supplementary Table S2. ECOG, Eastern Cooperative Oncology Group Performance Status; escBEACOPP, escalated bleomycin, etoposide, doxorubicin, cyclophosphamide, vincristine, procarbazine, prednisolone; IPS, International Prognostic Score; PET2, ^18^F-fluoro-deoxy-D-glucose positron emission tomography performed after two cycles of adriamycin, bleomycin, vinblastine, and dacarbazine. Data presented as N (%) for discrete rates and median [IQR] for numeric values. * *p*-values were calculated for the comparison ABVD PET2 positive vs. PET2 negative only. † Data about BEACOPP are presented for reference.

**Table 2:** Progression free and overall survival by Deauville and LLS.

	Pts.	PFS					OS				
		Events	Median Survival	3-Year PFS	HR	*p*	Events	Median Survival	5-Year OS	HR	*p*
LLS *											
qPET2−	170	25	NR	0.88 [0.83; 0.93]	1.0		6	NR [NR; NR]	0.96 [0.93; 1.0]	1.0	1.0
qPET2+	48	15	NR	0.70 [0.58; 0.85]	2.62 [1.38:4.97]	<0.001	4	102 [86; NR]	0.98 [0.94; 1.0]	2.61 [0.73:9.31]	0.14
Deauville ^†^											
dvPET2−	195	32	NR	0.86 [0.82; 0.91]	1.0		7	NA [102; NR]	0.97 [0.94; 1.0]	1.0	1.0
dvPET2+	23	8	NR	0.64 [0.47; 0.88]	2.65 [1.22:5.77]	0.01	3	86 [79; NR]	0.96 [0.88; 1.0]	6.15 [1.46:25.96]	0.01
escBEACOPP ^§^	9	5	39 [10; NR]	0.56 [0.31; 1.0]	4.53 [1.76:11.68]	<0.001	0	NR [NR; NR]	1.0 [1.0; 1.0]		

LLS, lesion SUVmax to liver SUVmean ratio; NR not reached; OS, overall survival; PFS, progression free survival. * Per LLS: qPET2− refers to LLS < 1.3 and qPET2+ refers to LLS ≥ 1.3 ^†^ Per Deauville: dvPET2− refers to Deauville ≤ 3 and dvPET2+ refers to Deauville ≥ 4. ^§^ Data of about 9 patients switched to escBEACOPP are displayed for reference only.

## Data Availability

The data presented in this study are available on request from the corresponding author.

## Supplementary Materials

The following supporting information can be downloaded at: https://www.mdpi.com/article/10.3390/cancers15061760/s1, Figure S1: CONSORT—Response and use of radiotherapy; Table S1: Selected studies reporting PET2 on ABVD; Table S2: Descriptive statistics by dvPET2 status; File S1: Images and clinical data of interim PET+ cases (qPET2+ and/or dvPET2+).
