# Supplementary material for: Outcomes of Patients with Positive Interim Positron Emission Tomography (PET) Continuing ABVD in the Clinical Setting
Source: Cancers (Basel). 2023 Mar 14;15(6):1760. doi: 10.3390/cancers15061760 (PMC10046293; doi:10.3390/cancers15061760)

Supplementary Material

Supp. table S1 - Selected studies reporting PET2 on ABVD

Study	N	PET2+ (%, n)	Treatment failure PET2+ (POD + PR + SD)	3y PFS PET2-	3y PFS PET2+	Comment*
Gallamini (2007)	260	19%*	88%	2yPFS 95%*	13%	2y PFS (not 3y)<br>PET2+ by IHP (~Deauville >2)
Biggi (2013)	260	17% (45)	NA	95%*	Central review 28%<br>Local review 54%	FFS NOT PFS; 25% of PET2+ were FP<br>Excluded pts. w/ escalation based on PET2<br>8 patients (3+) w/o consensus were PET2+
Oki (2014)	174	17%	NA	83%	~48%	
Zinzani (2012)	304	22%	62%	NA	~40%*	Patient with EOT PET PR to further therapy w/o biopsy = event
Johnson (2016)<br>RATHL	1119	16% (160)	25% *<br>(on escBEACOPP)	85%	68%<br>(on escBEACOPP)	24 pts. Received further chemo for consolidation after escBEACOPP and 43 RT
Zinzani (2016)<br>HD0801	512	20% (103)*<br>14% DV >3 (72)	50%	2yPFS 81%	2yPFS 76%<br>(IGEV+ASCT)	30% of PET2+ cases that went to intensive treatment were actually Deauville 3<br>15 PET2+ continued ABVD with 73% CR
Press (2016)<br>SWOG S0816	331	18% (61)	45%*<br>(on escBEACOPP)	2yPFS 82%	2yPFS 64%<br>(on escBEACOPP)	38% PET-PR data not provided on RT additional therapy; 7% SD/UNK
Straus (2021)<br>ECHELON-1	636	**9%**	NA	3yPFS 76%	3yPFS 55%*	Censoring mPFS at the modified event may have caused an underestimation of PFS. 5y-PFS recently published down to 46%
**MSK**	227	14%/25%	13%	86%	64%*<br>(by dvPET2+)	If we were to include transition to escBEACOPP as event (PFS 23% if all cases = events<br>PFS 61% if only POD on escBEACOPP = event)

Supp. figure S1 - CONSORT – Response and use of radiotherapy

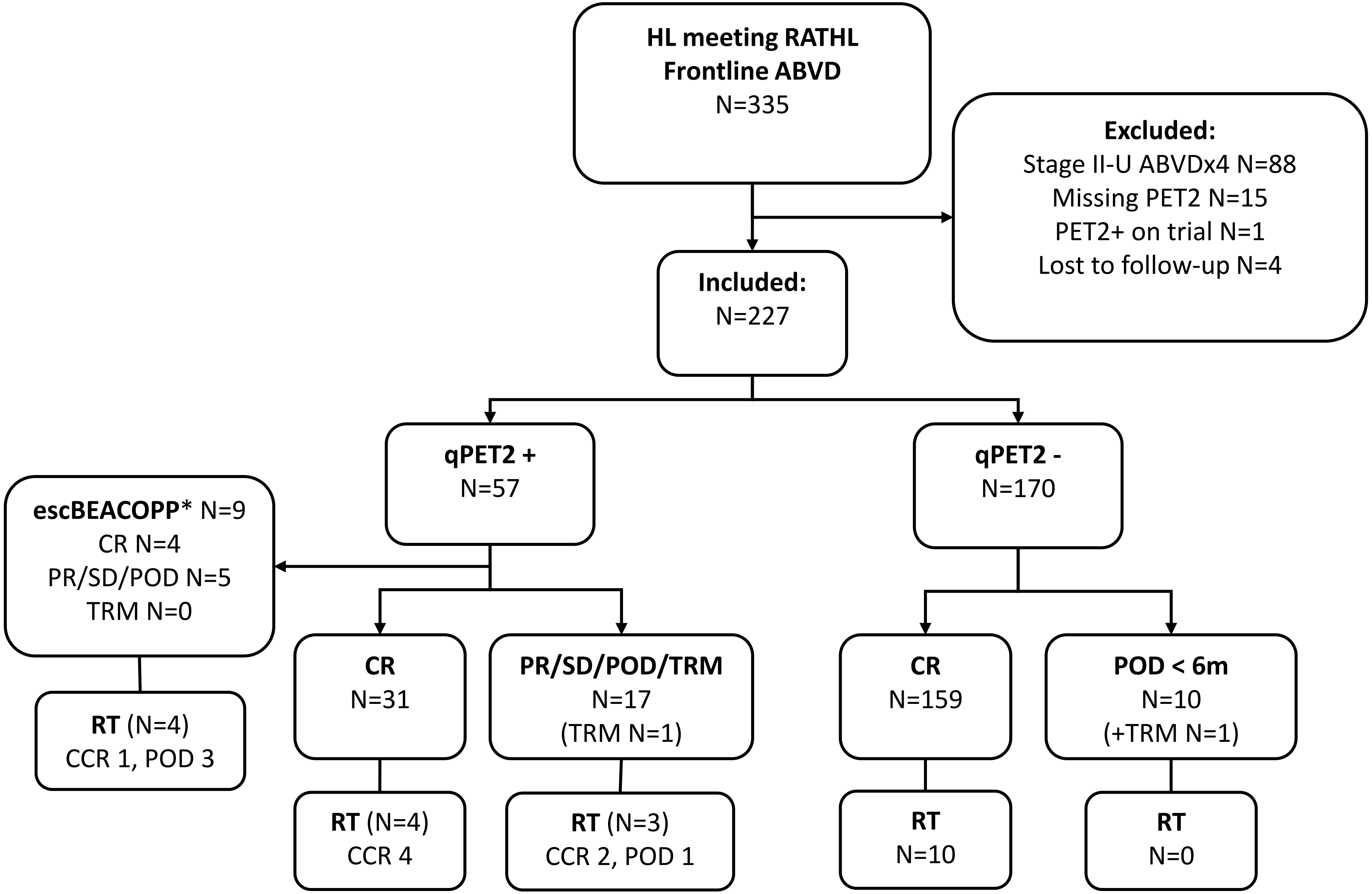

Images and clinical data of interim PET+ cases (qPET2+ and/or dvPET2+)

Images are ordered from left to right
Diagnosis – Interim – End of Therapy – [subsequent scans]

Abbreviations

- qPET2+ - lesion SUVmax to liver SUVmean ≥ 1.3
- dvPET2+ - Deauville score 4-5
- OSH – imaging loaded only to main radiology system (not available for download)
- POD – Progression of disease
- Tx 2 – second line treatment

- IPS – International prognostic score
- SUV ratio – lesion SUVmax to liver SUVmean
- SUV – SUV max @ lesion
- Liver – SUVmean @ liver
- Response – by imaging prior to any radiotherapy (imaging PR with negative biopsy or no progression on short follow-up -> CR)
- RT – radiotherapy after frontline therapy
- RT resp – response after radiotherapy
- Rel prev site – relapse at site of initial diagnosis
- Bx confirmed – was biopsy done to confirm relapse

escBEACOPP
Patients with POD

MRN	Age	IPS	Escalated	Cycles	SUV Ratio	Deauville	SUV	Liver	Response	RT	RT resp	Relapse	PFS	Death	OS	Rel prev site	Bx confirmed
	53	4-7	BEACOPP	6	>=1.3	>=4	10	1.8	PR	1	POD	1	12	0	40	1	1

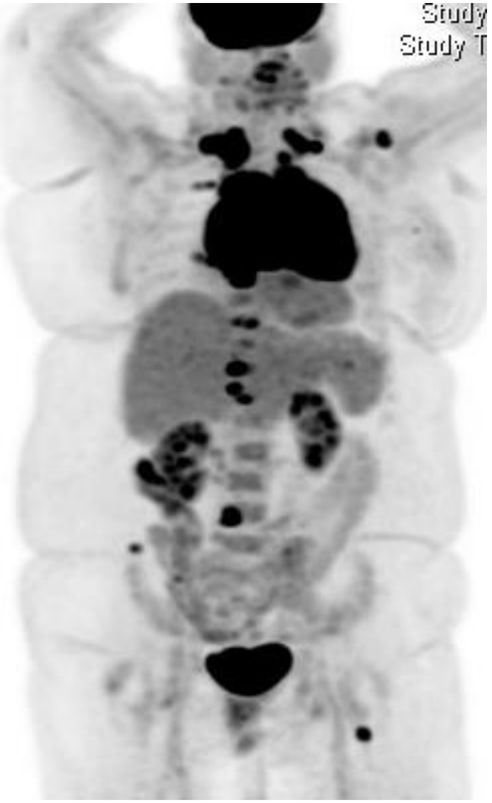

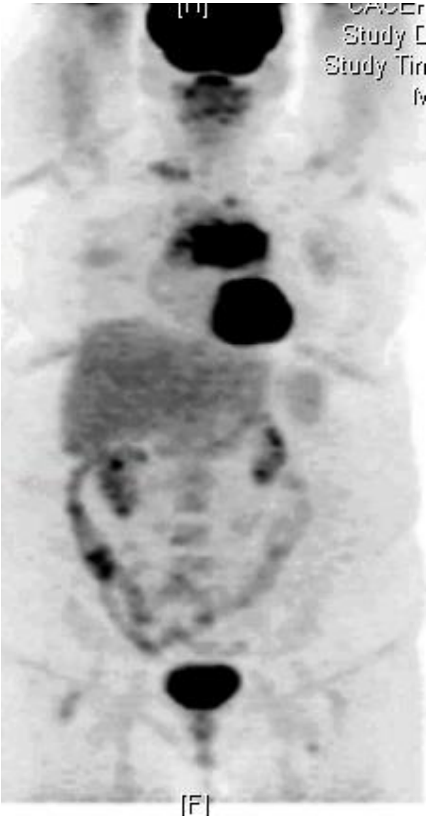

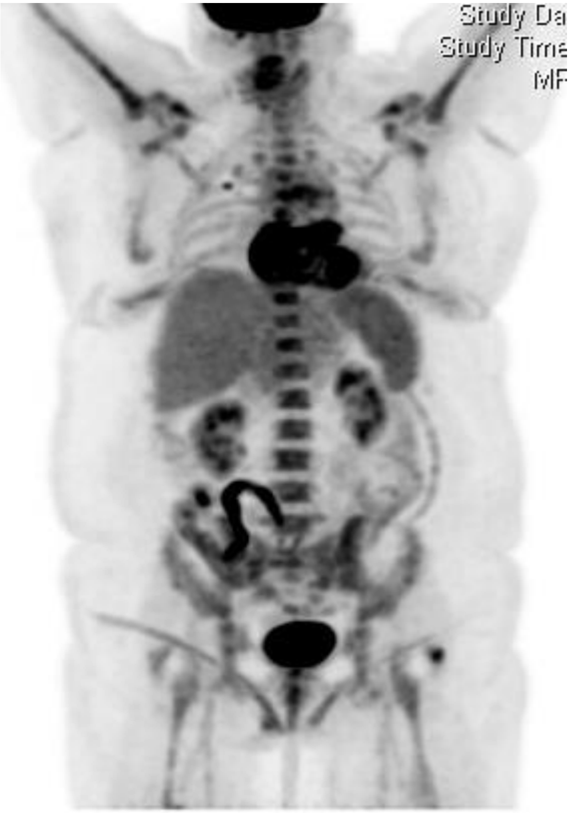

Tx-2
BV-ICE - ASCT

MRN	Age	IPS	Escalated	Cycles	SUV Ratio	Deauville	SUV	Liver	Response	RT	RT resp	Relapse	PFS	Death	OS	Rel prev site	Bx confirmed
	36	0-1	BEACOPP	6	>=1.3	>=4	5.2	2.3	POD	0		1	7	0	35	1	1

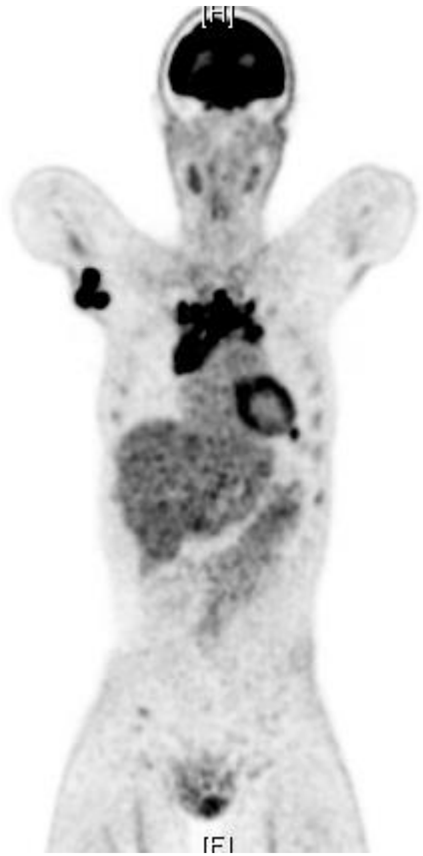

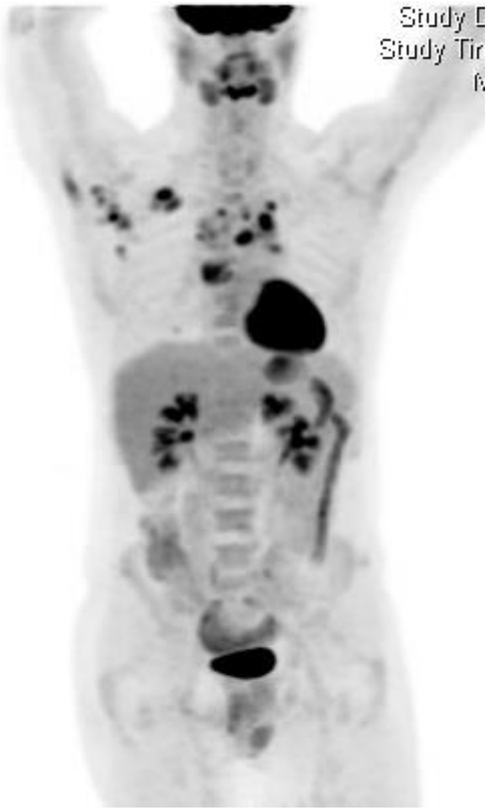

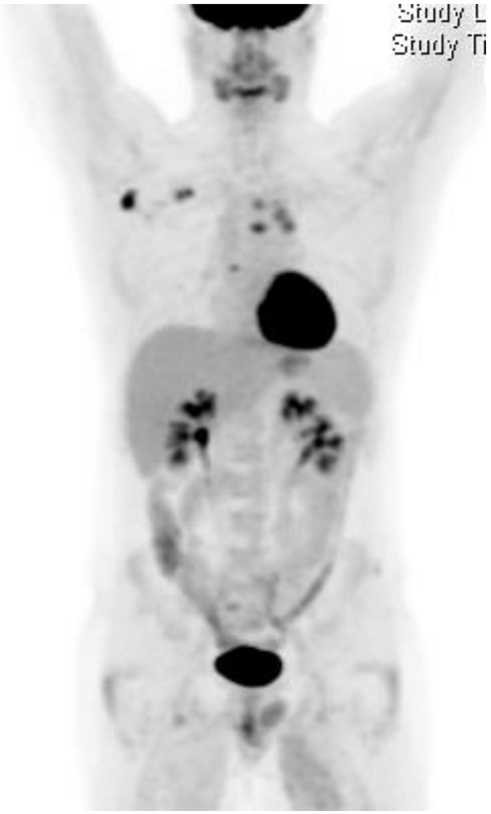

Tx-2
BV + RT -> ASCT

MRN	Age	IPS	Escalated	Cycles	SUV Ratio	Deauville	SUV	Liver	Response	RT	RT resp	Relapse	PFS	Death	OS	Rel prev site	Bx confirmed
	21	0-1	BEACOPP	6	>=1.3	>=4	9	1.55	POD	1	POD	1	10	0	90	1	1

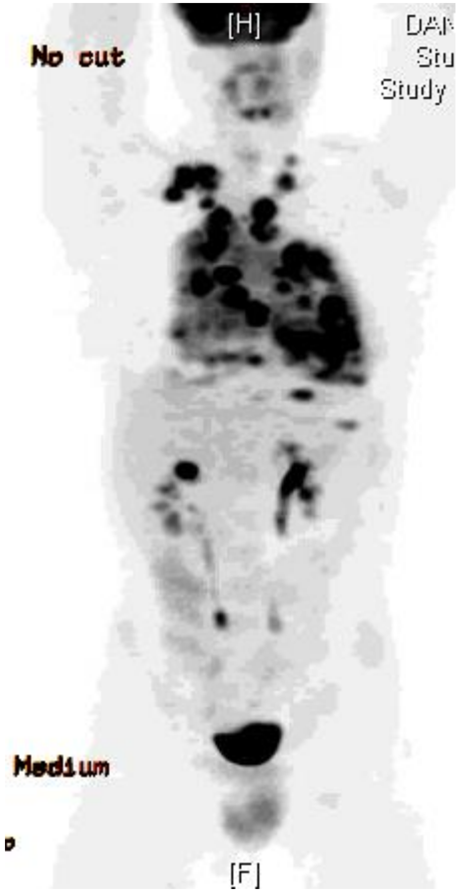

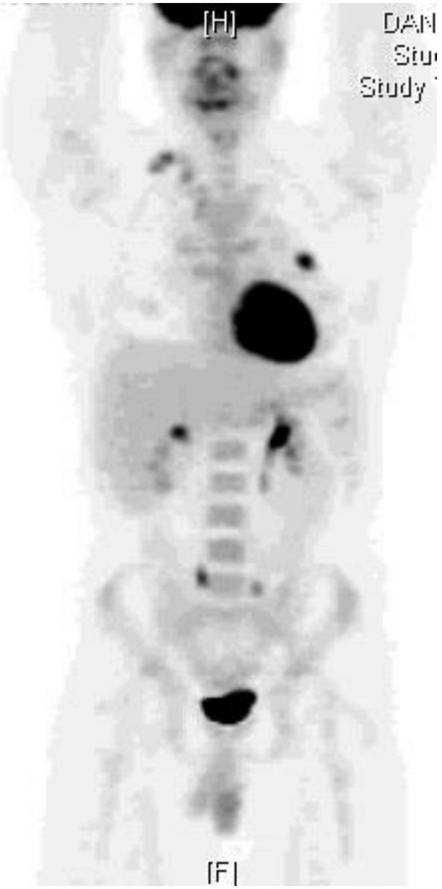

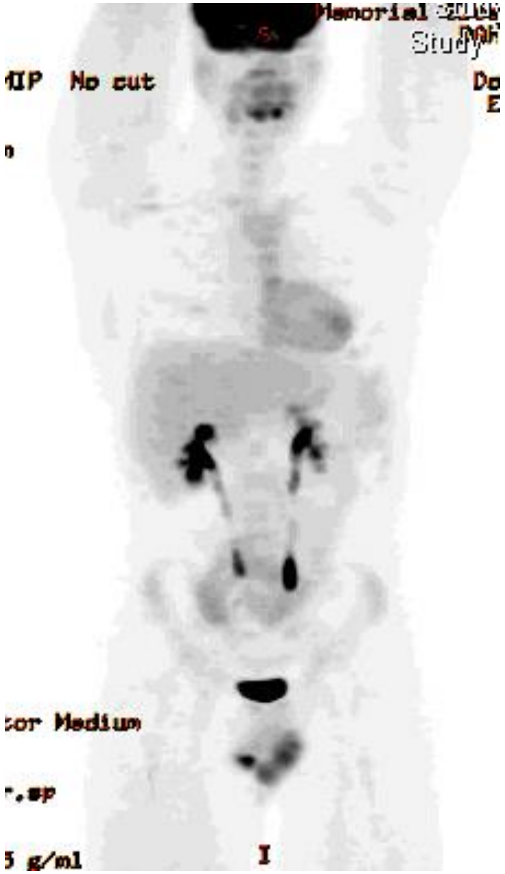

EOT

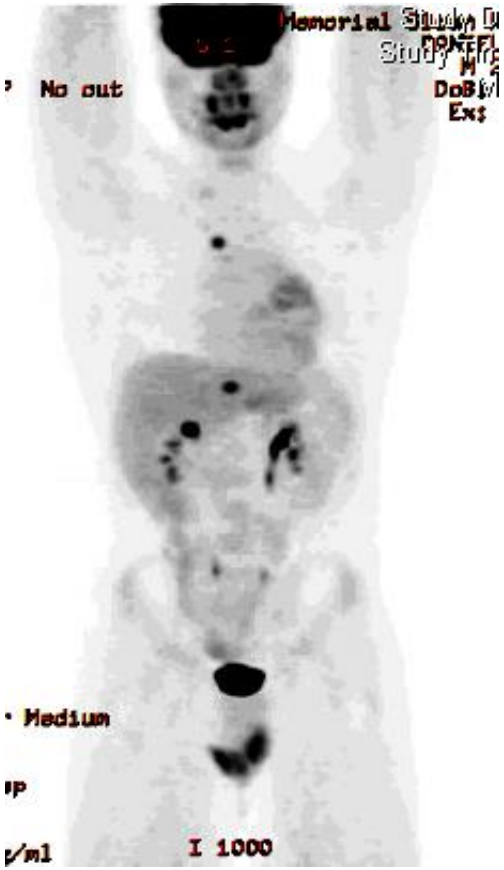

Relapsed w/in 6 mo

Tx-2
Investigational + ICE -> ASCT

MRN	Age	IPS	Escalated	Cycles	SUV Ratio	Deauville	SUV	Liver	Response	RT	RT resp	Relapse	PFS	Death	OS	Rel prev site	Bx confirmed
	25	0-1	BEACOPP	6	>=1.3	>=4	7.8	1.63	POD	1	POD	1	9	0	27	1	1

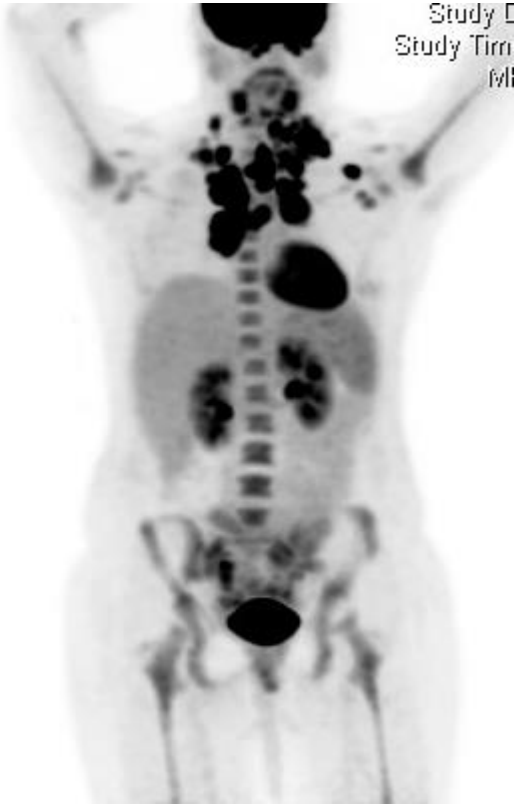

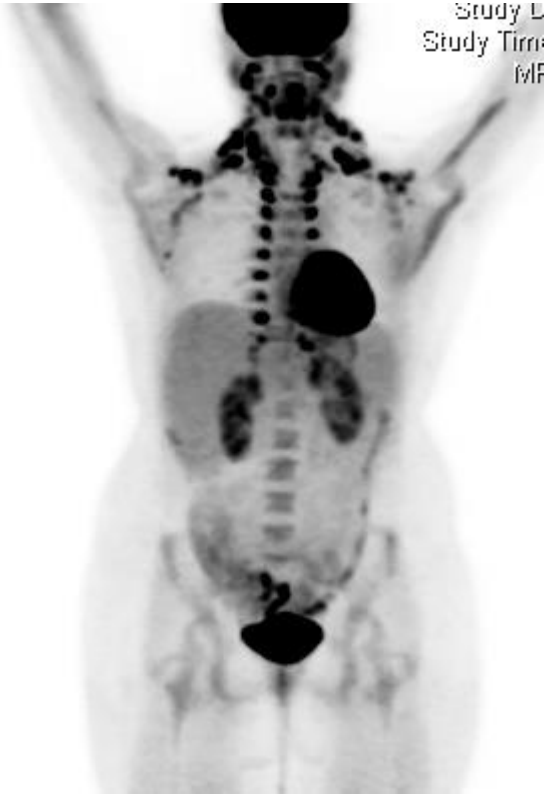

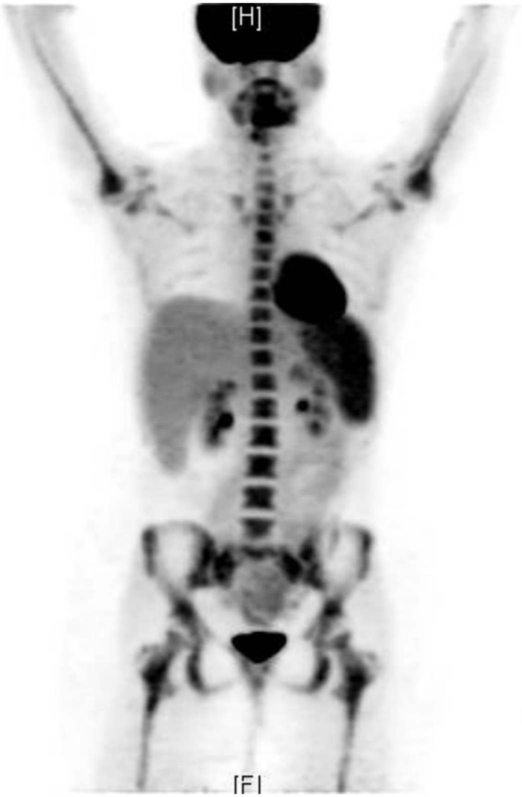

EOT CR

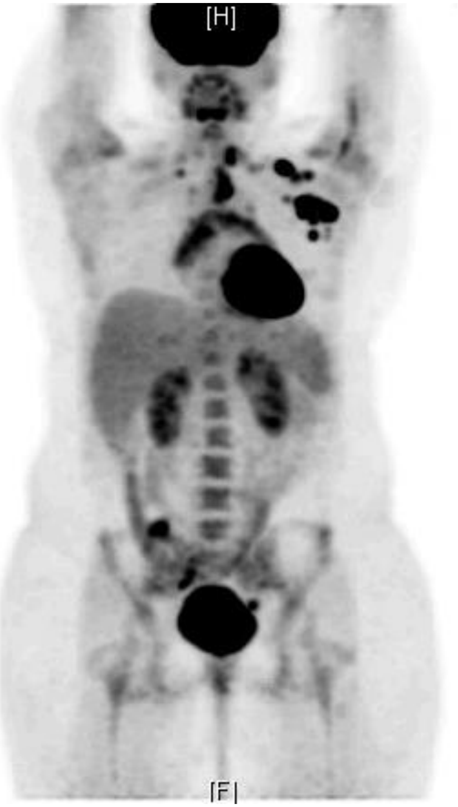

Relapsed within 5 mo

Tx-2
BV-ICE - ASCT

MRN	Age	IPS	Escalated	Cycles	SUV Ratio	Deauville	SUV	Liver	Response	RT	RT resp	Relapse	PFS	Death	OS	Rel prev site	Bx confirmed
	50	2-3	BEACOPP	6	>=1.3	>=4	5	2.78	CR	0		1	39	0	99	1	1

Note: relapse after 39m

Tx-2
BV + RT -> alloSCT

OSH

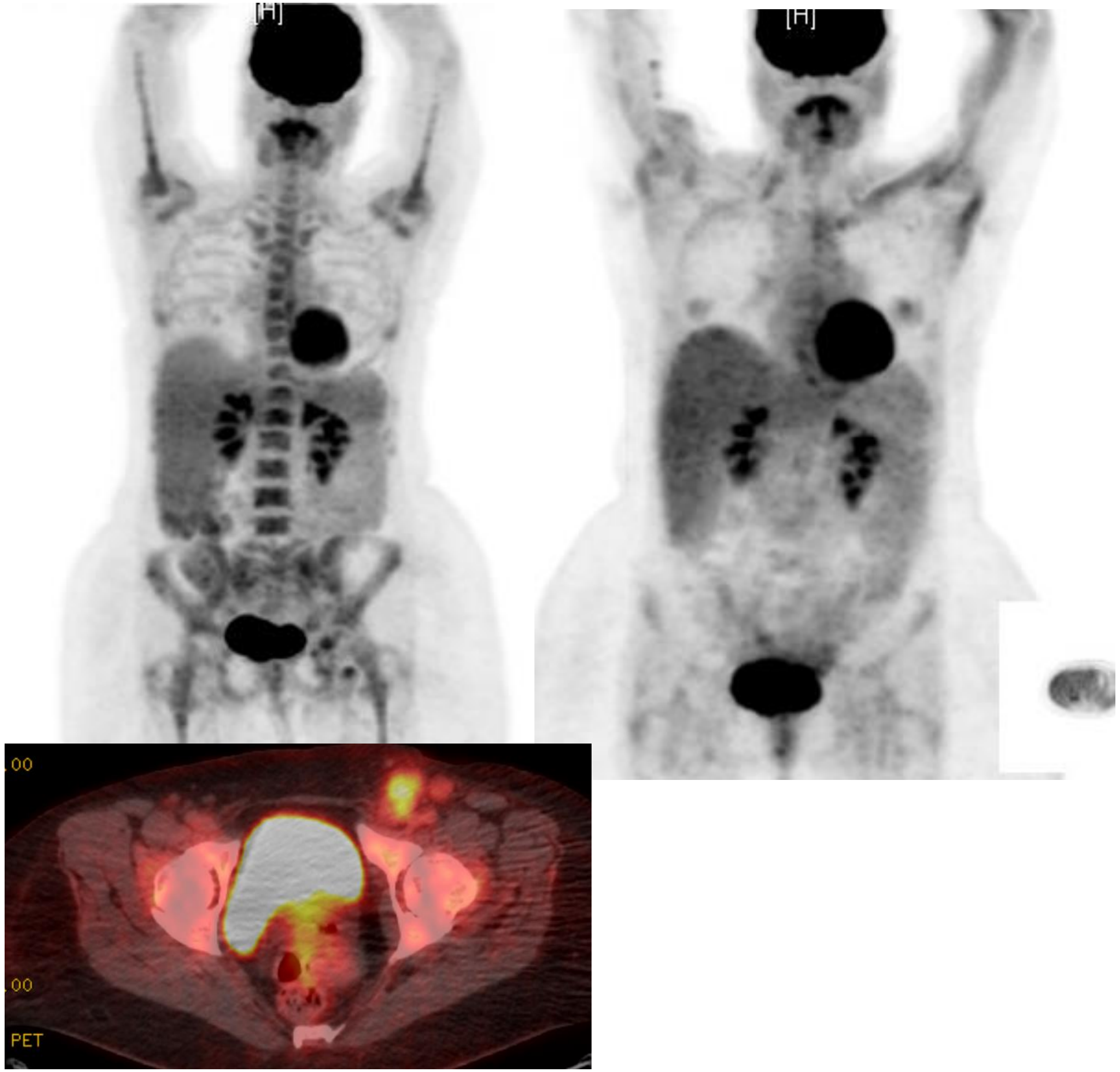

escBEACOPP
Patients with long-term remission

MRN	Age	IPS	Escalated	Cycles	SUV Ratio	Deauville	SUV	Liver	Response	RT	RT resp	Relapse	PFS	Death	OS	Rel prev site	Bx confirmed
	19	0-1	BEACOPP	6	**>=1.3**	>=4	8	2.4	CR	0		**0**	**63**	0	63		

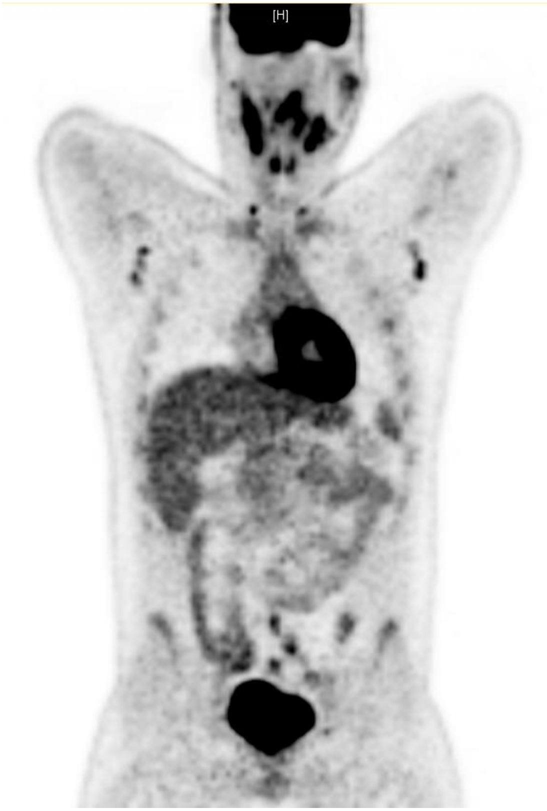

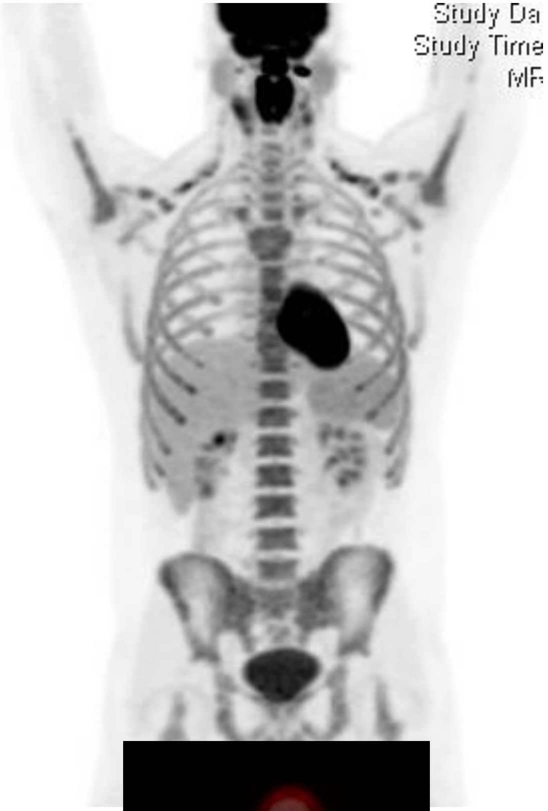

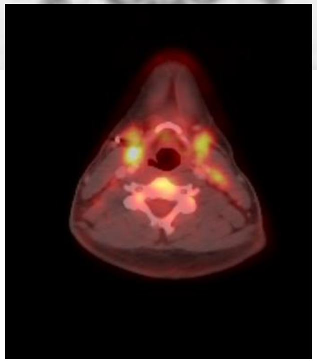

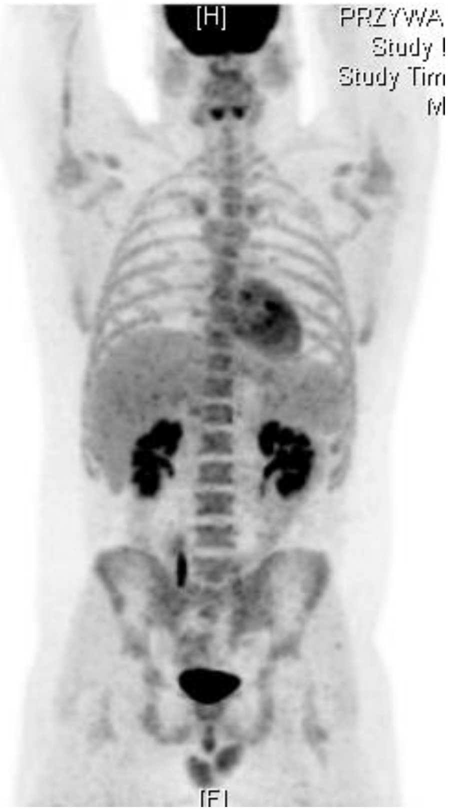

MRN	Age	IPS	Escalated	Cycles	SUV Ratio	Deauville	SUV	Liver	Response	RT	RT resp	Relapse	PFS	Death	OS	Rel prev site	Bx confirmed
	59	4-7	**BEACOPP**	8	>=1.3	>=4	**5.2**	**2.3**	CR	0		0	24	0	24		

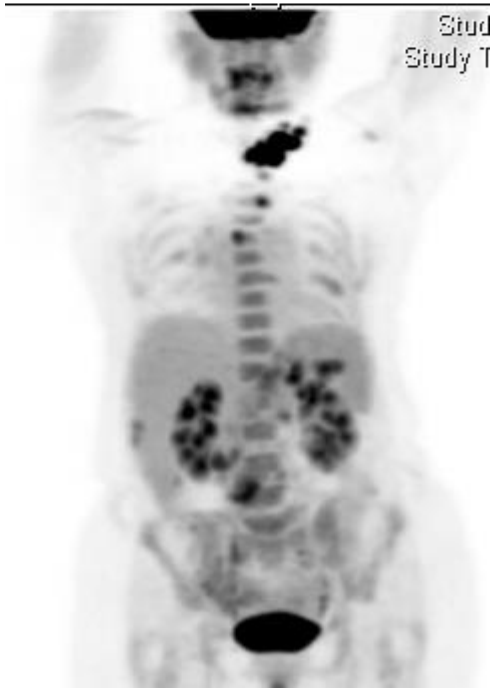

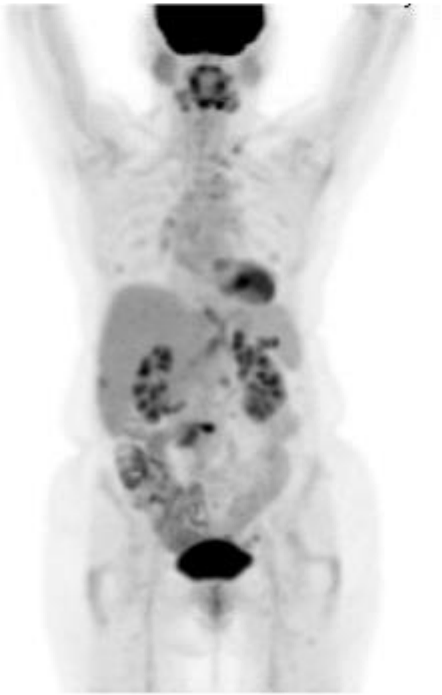

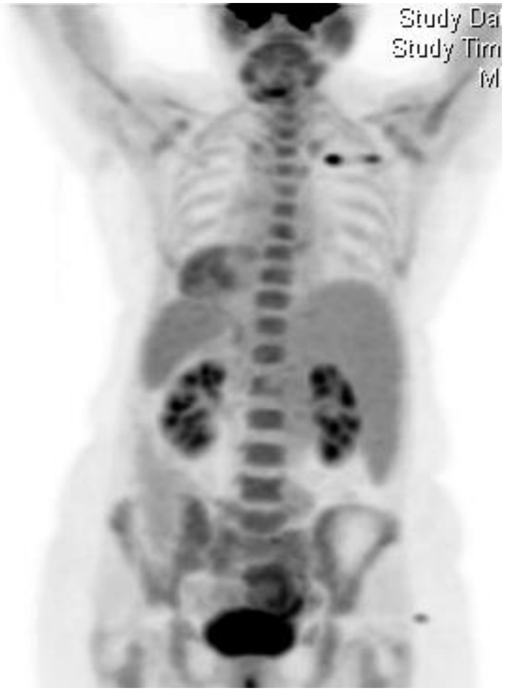

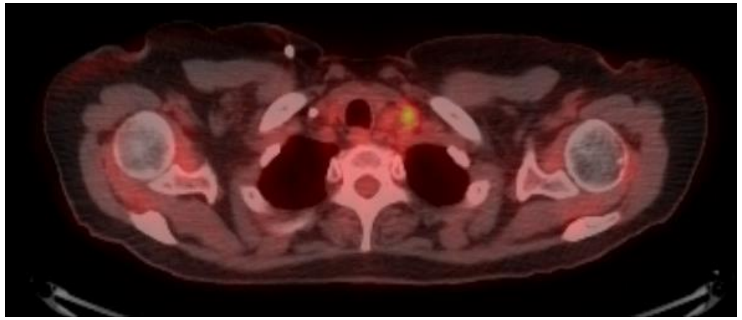

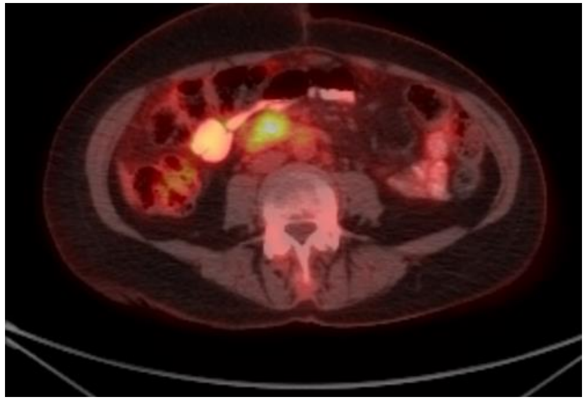

MRN	Age	IPS	Escalated	Cycles	SUV Ratio	Deauville	SUV	Liver	Response	RT	RT resp	Relapse	PFS	Death	OS	Rel prev site	Bx confirmed
	19	2-3	BEACOPP	5	>=1.3	>=4	6.4	2.1	PR	1	CR	0	35	0	35		

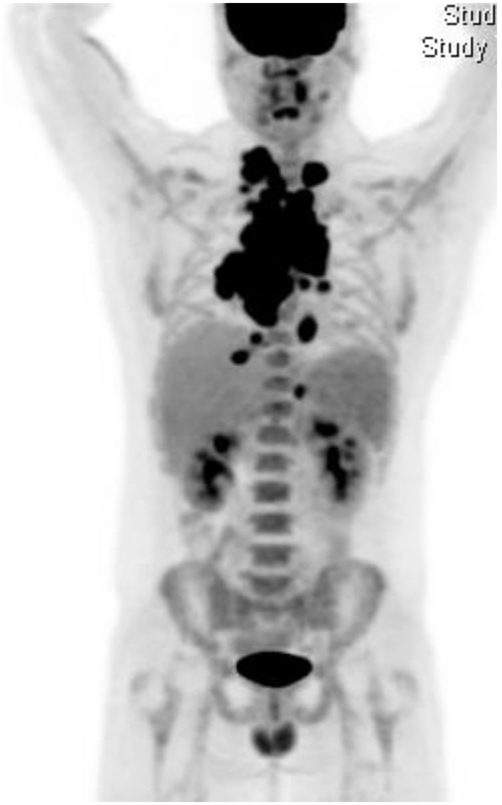

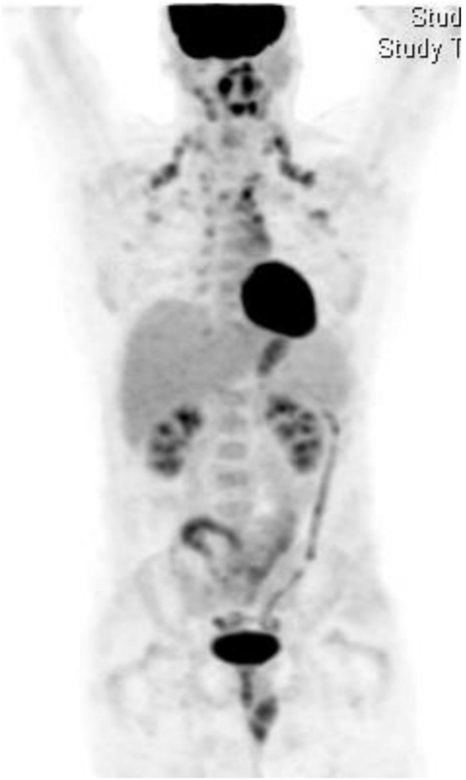

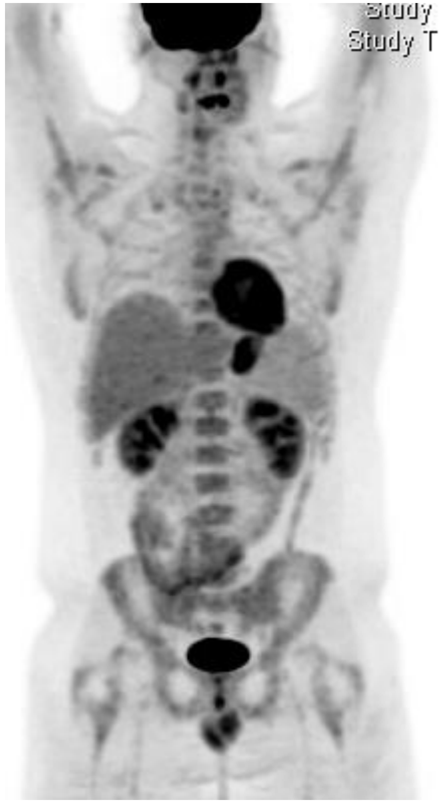

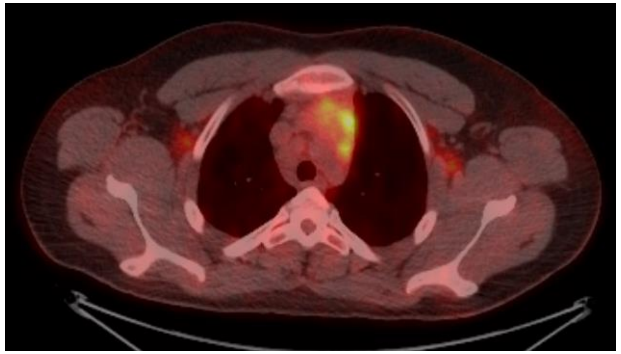

MRN	Age	IPS	Escalated	Cycles	SUV Ratio	Deauville	SUV	Liver	Response	RT	RT resp	Relapse	PFS	Death	OS	Rel prev site	Bx confirmed
	32	4-7	BEACOPP	6	>=1.3	<=3	2.7	1.8	CR	0		0	52	0	52		

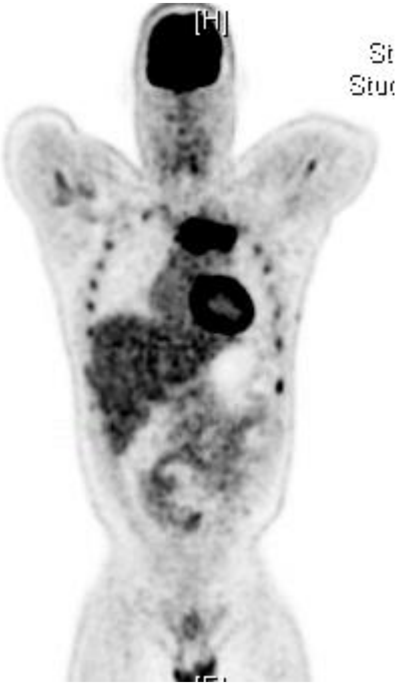

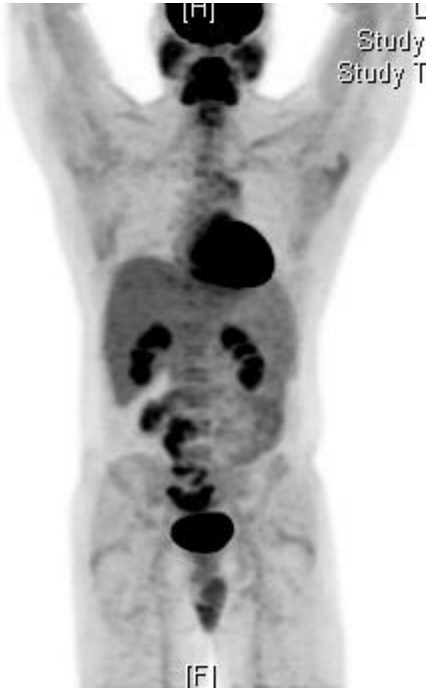

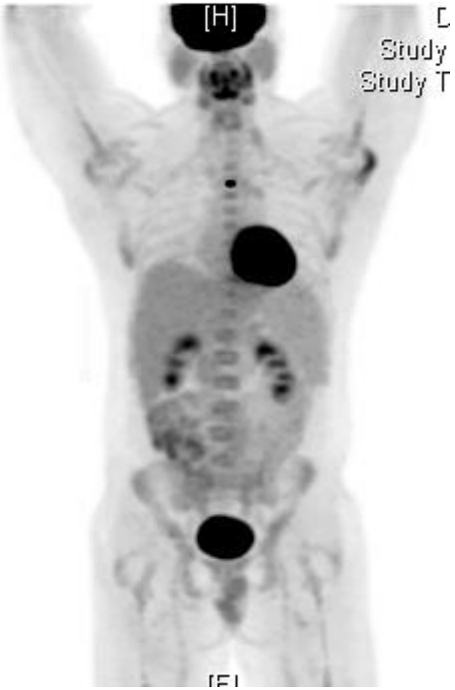

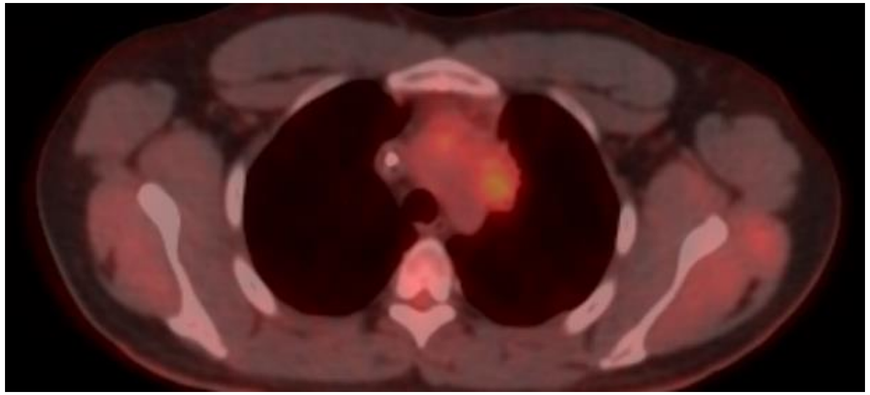

dvPET2+ (Deauville)
Patients with long-term Remission

MRN	Age	IPS	Escalated	Cycles	SUV Ratio	Deauville	SUV	Liver	Response	RT	RT resp	Relapse	PFS	Death	OS	Rel prev site	Bx confirmed
	39	0-1	ABVD	6	>=1.3	>=4	10.8	3.1	CR	0		0	42	0	42		NED

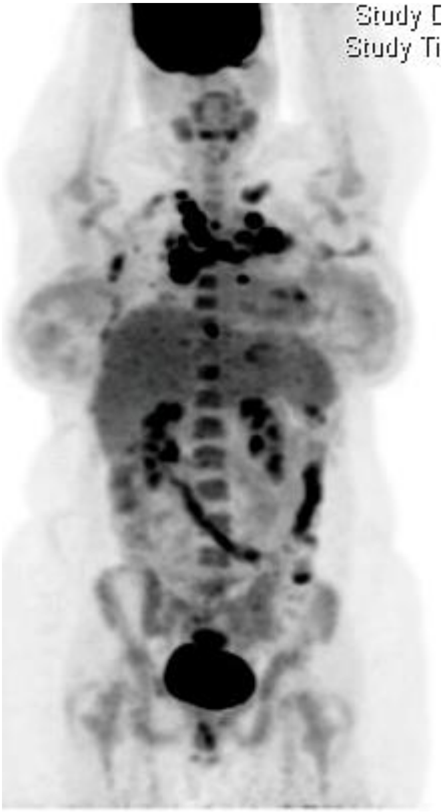

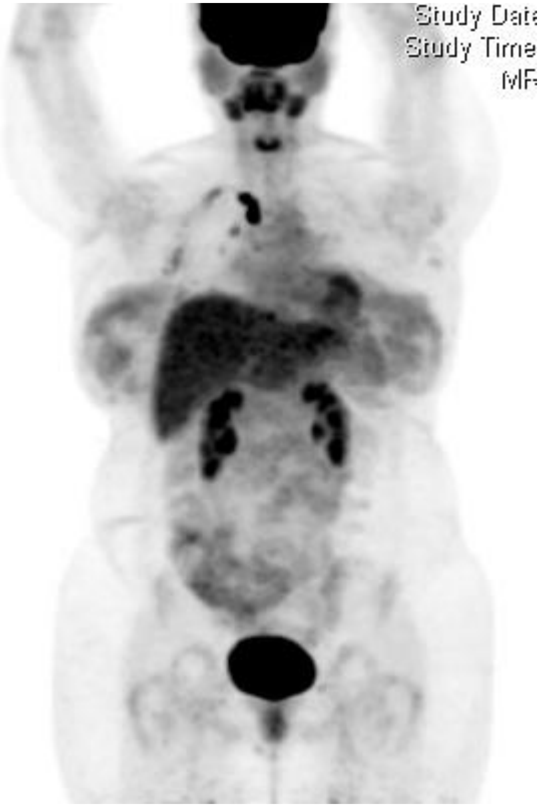

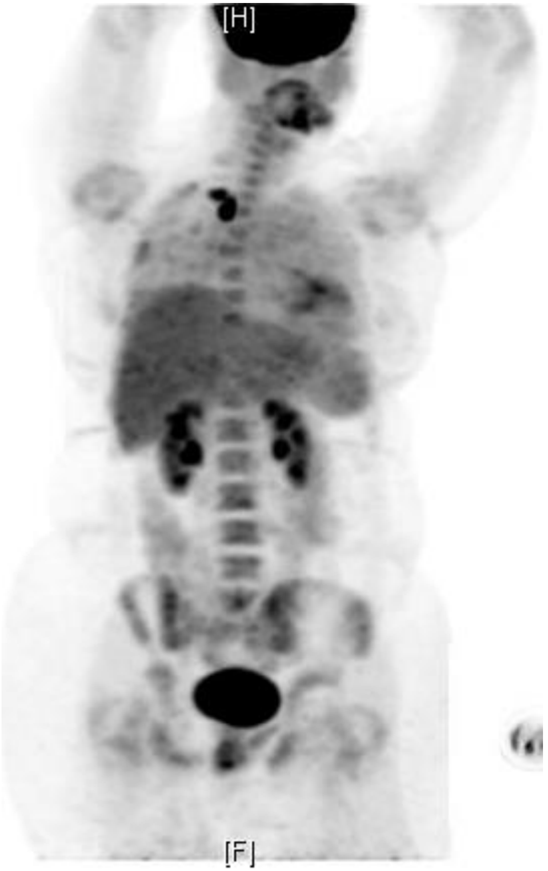

Biopsy w/o HL

MRN	Age	IPS	Escalated	Cycles	SUV Ratio	Deauville	SUV	Liver	Response	RT	RT resp	Relapse	PFS	Death	OS	Rel prev site	Bx confirmed
	47	2-3	ABVD	6	>=1.3	>=4	7.4	1.4	PR	1		0	84	0	84		NED

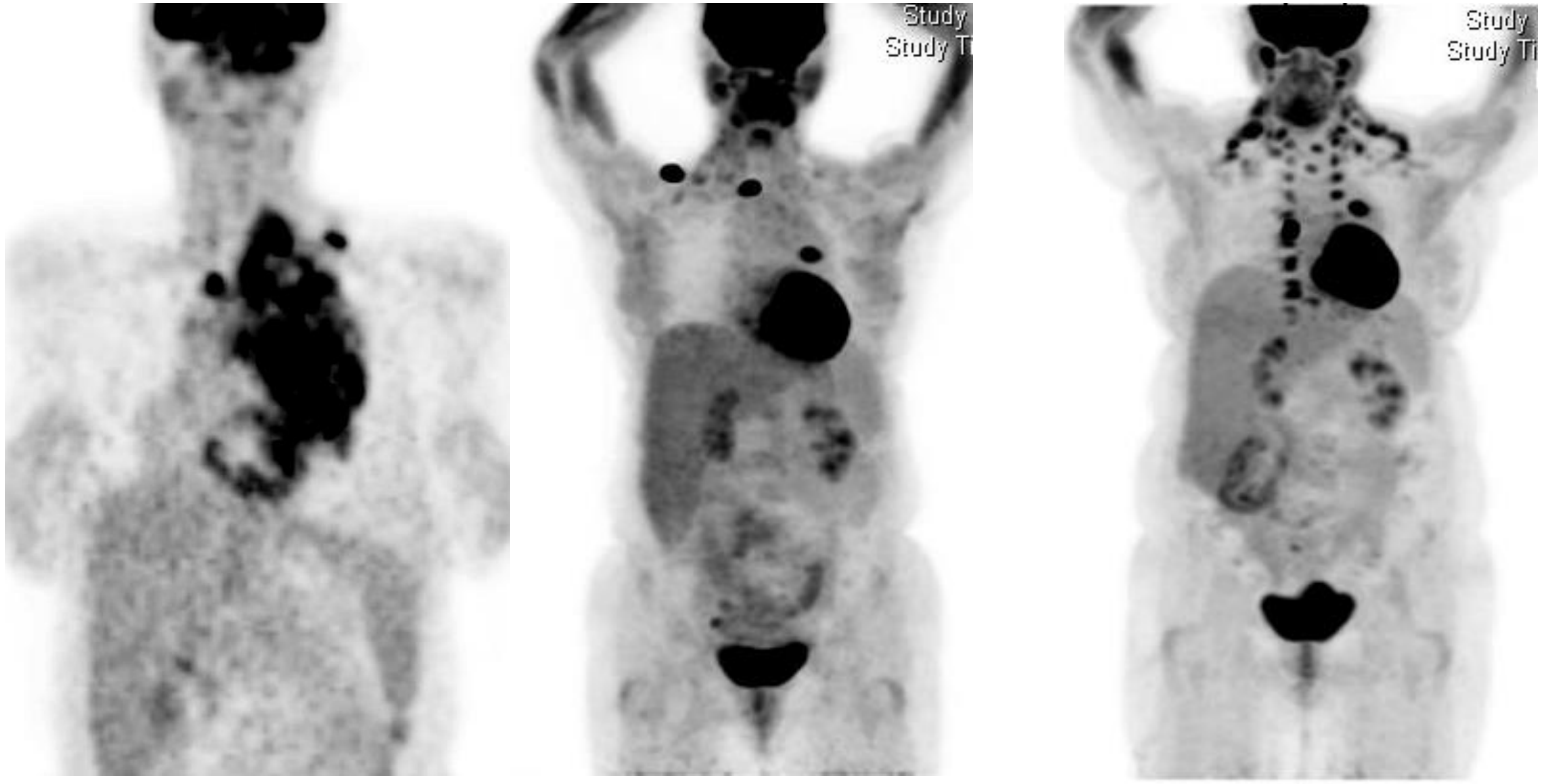

Persistent FDG avidity at EOT and after RT with biopsy x2
without evidence of HL (histiocytic infiltrate)

MRN	Age	IPS	Escalated	Cycles	SUV Ratio	Deauville	SUV	Liver	Response	RT	RT resp	Relapse	PFS	Death	OS	Rel prev site	Bx confirmed
	19	0-1	ABVD	6	>=1.3	>=4	5.5	1.7	CR	1		0	60	0	60		

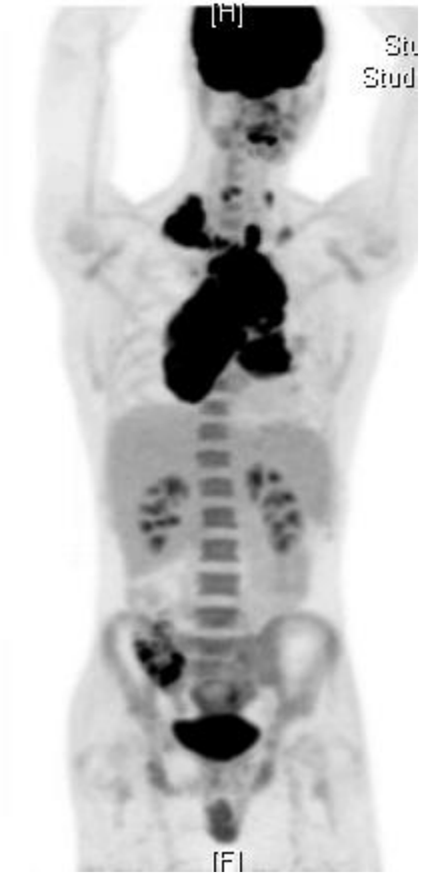

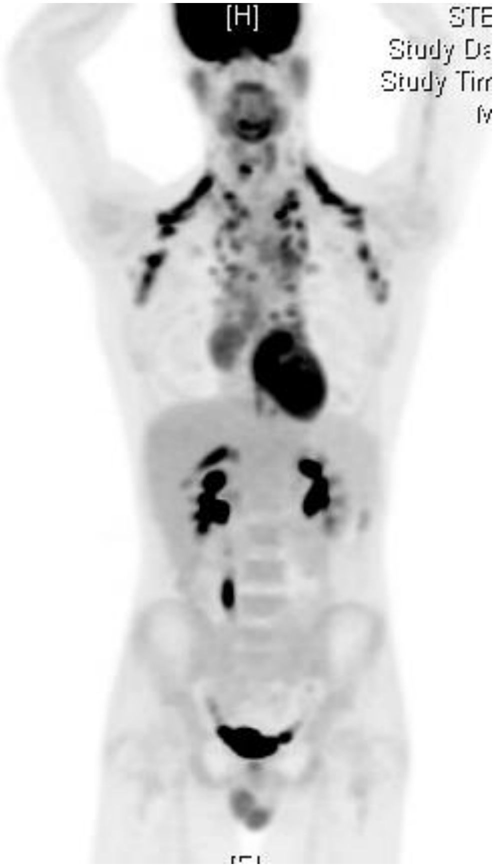

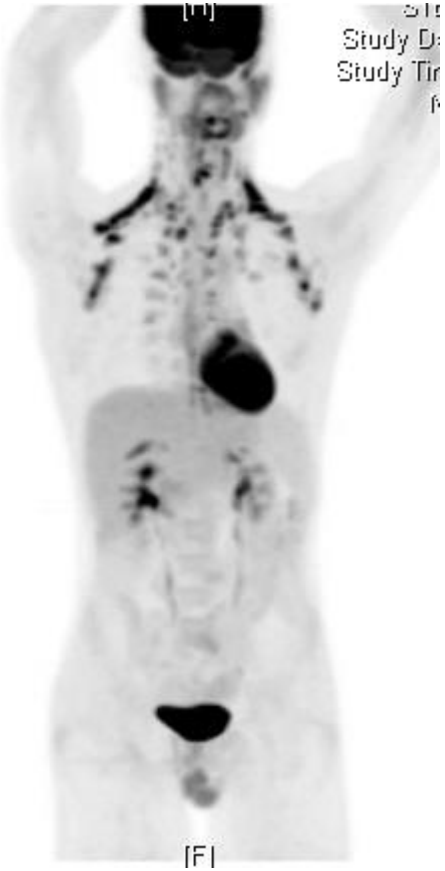

Before RT

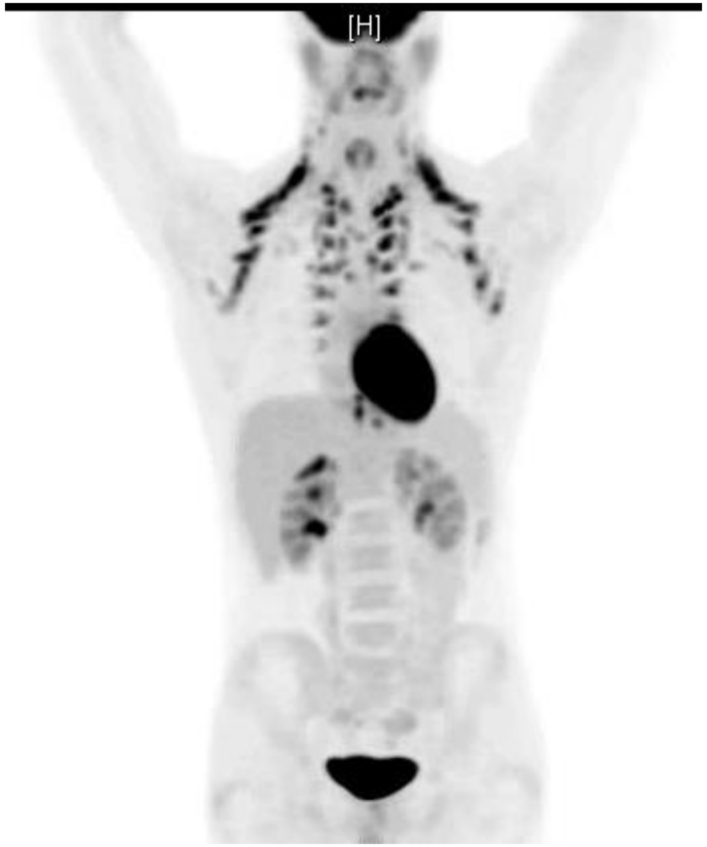

After RT

MRN	Age	IPS	Escalated	Cycles	SUV Ratio	Deauville	SUV	Liver	Response	RT	RT resp	Relapse	PFS	Death	OS	Rel prev site	Bx confirmed
	23	0-1	ABVD	6	>=1.3	>=4	3.6	1.6	CR	0		0	77	0	77		

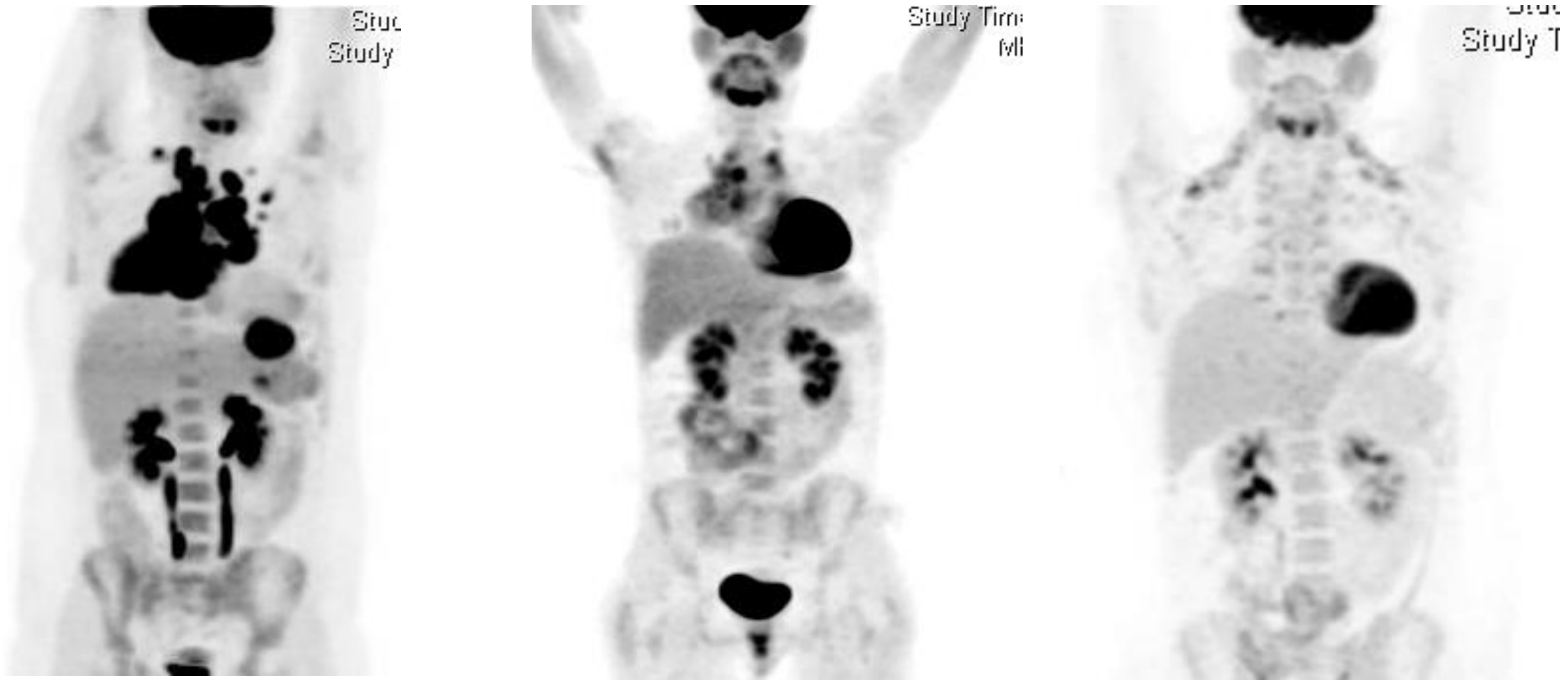

MRN	Age	IPS	Escalated	Cycles	SUV Ratio	Deauville	SUV	Liver	Response	RT	RT resp	Relapse	PFS	Death	OS	Rel prev site	Bx confirmed
	26	0-1	ABVD	6	>=1.3	>=4	2.8	1.68	CR	1		0	49	0	49		

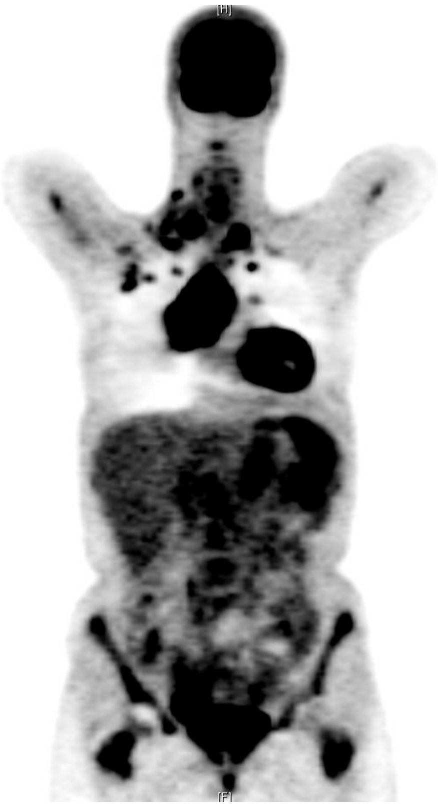

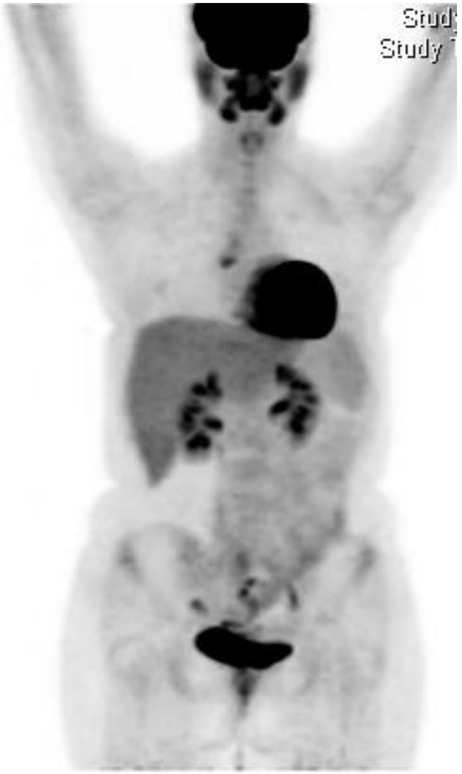

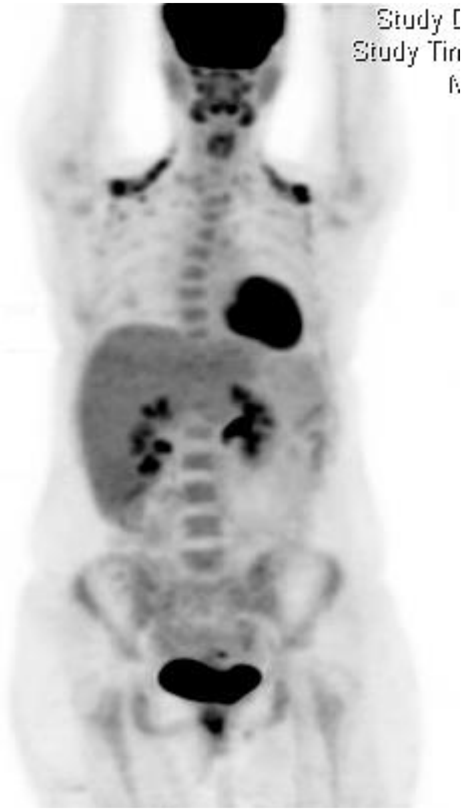

MRN	Age	IPS	Escalated	Cycles	SUV Ratio	Deauville	SUV	Liver	Response	RT	RT resp	Relapse	PFS	Death	OS	Rel prev site	Bx confirmed
	37	4-7	ABVD	6	>=1.3	>=4	4.6	2	CR	0		0	25	0	25		

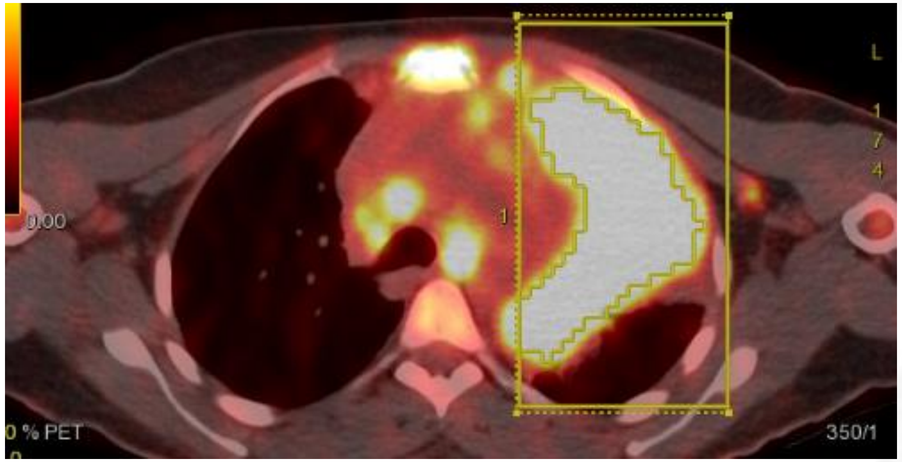

OSH

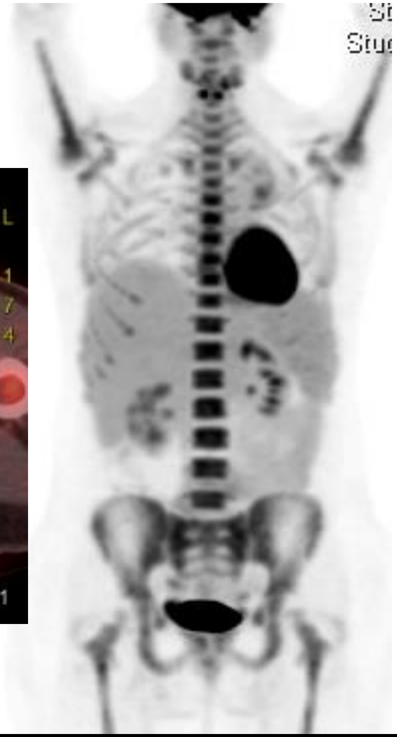

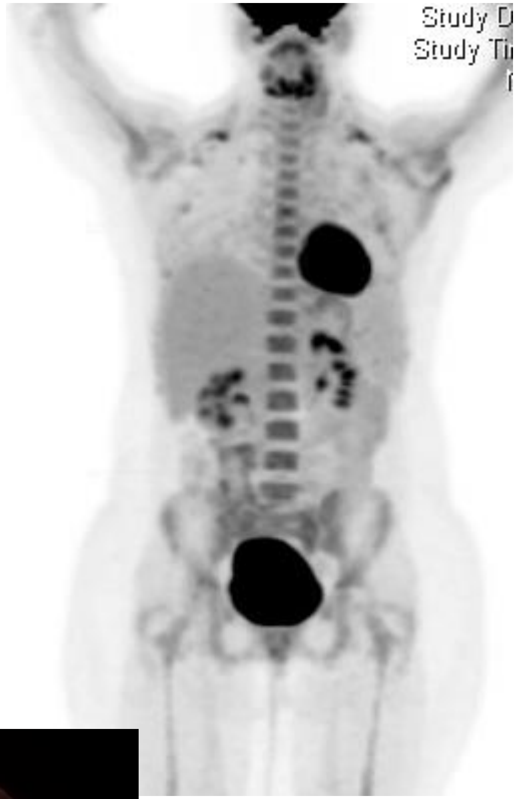

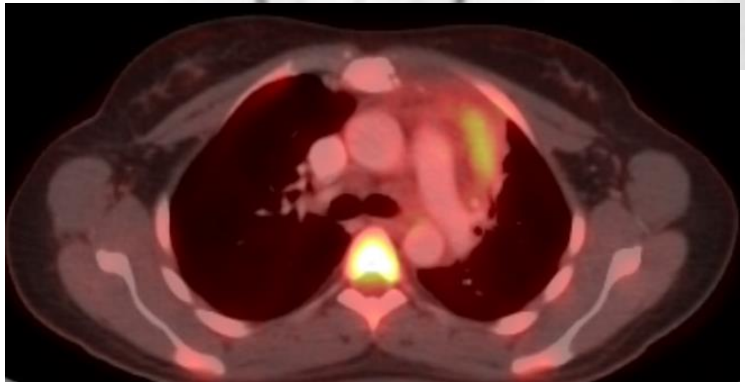

MRN	Age	IPS	Escalated	Cycles	SUV Ratio	Deauville	SUV	Liver	Response	RT	RT resp	Relapse	PFS	Death	OS	Rel prev site	Bx confirmed
	76	4-7	ABVD	6	>=1.3	>=4	5.2	3	CR	0		0	23	0	23		

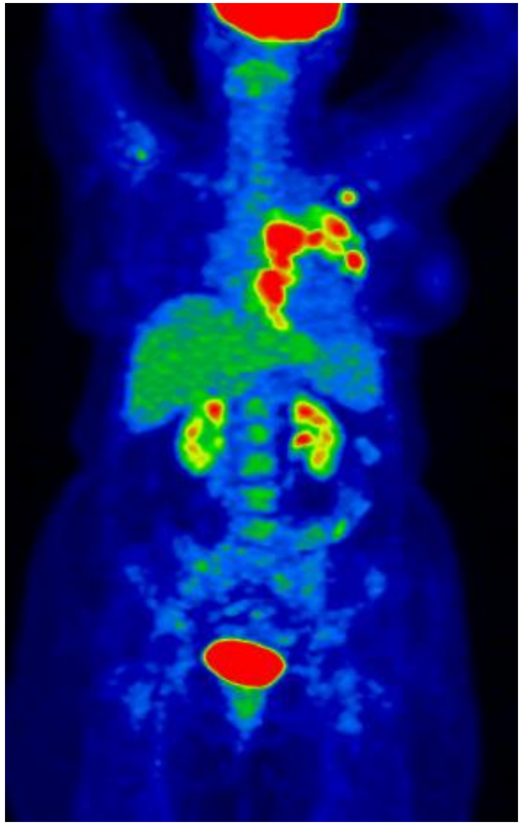

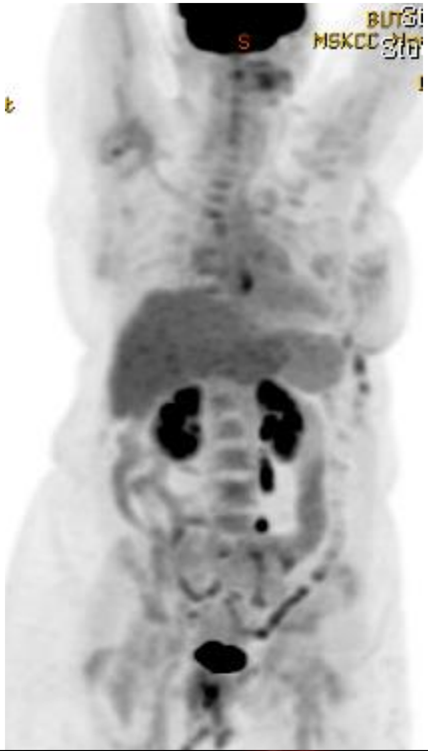

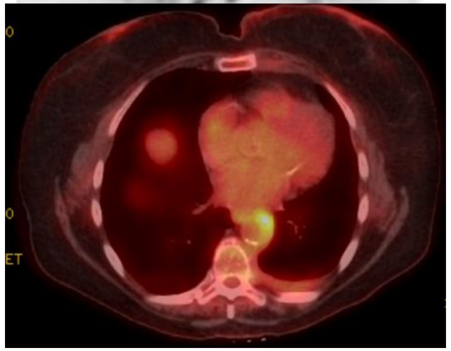

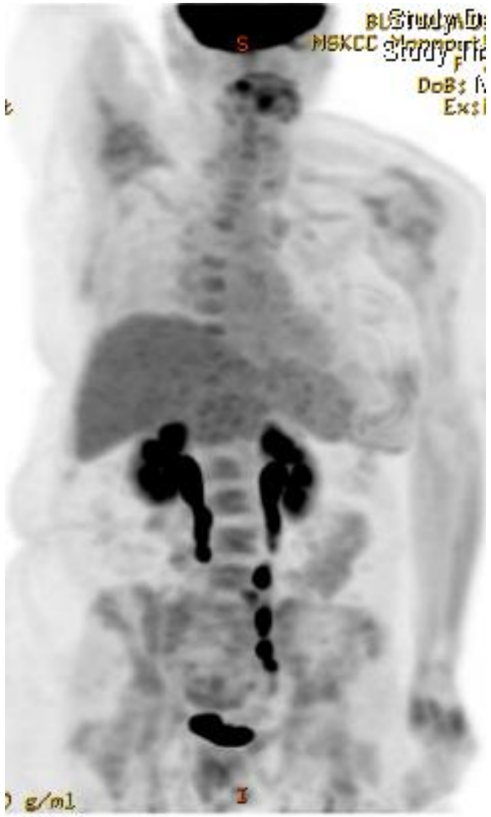

MRN	Age	IPS	Escalated	Cycles	SUV Ratio	Deauville	SUV	Liver	Response	RT	RT resp	Relapse	PFS	Death	OS	Rel prev site	Bx confirmed
	61	2-3	ABVD	6	>=1.3	>=4	4.4	1.9	PR	0		0	17	0	17		

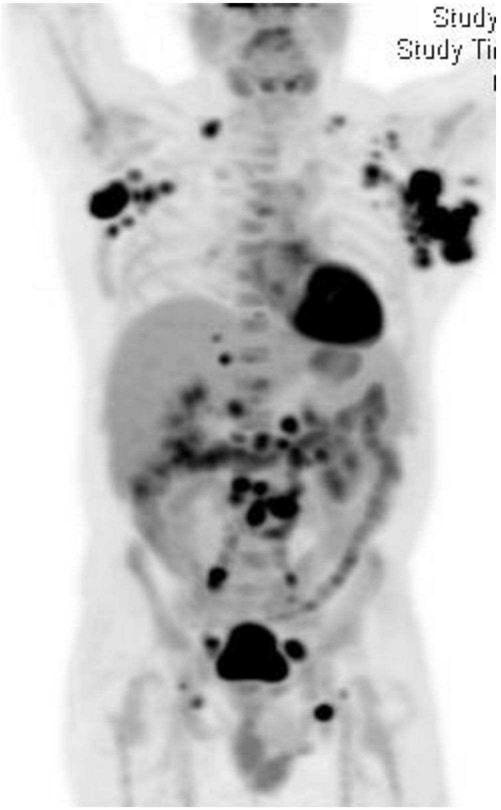
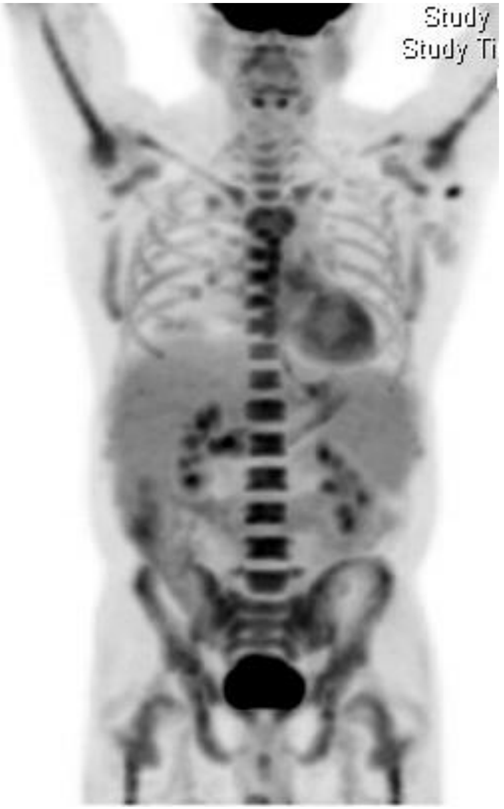
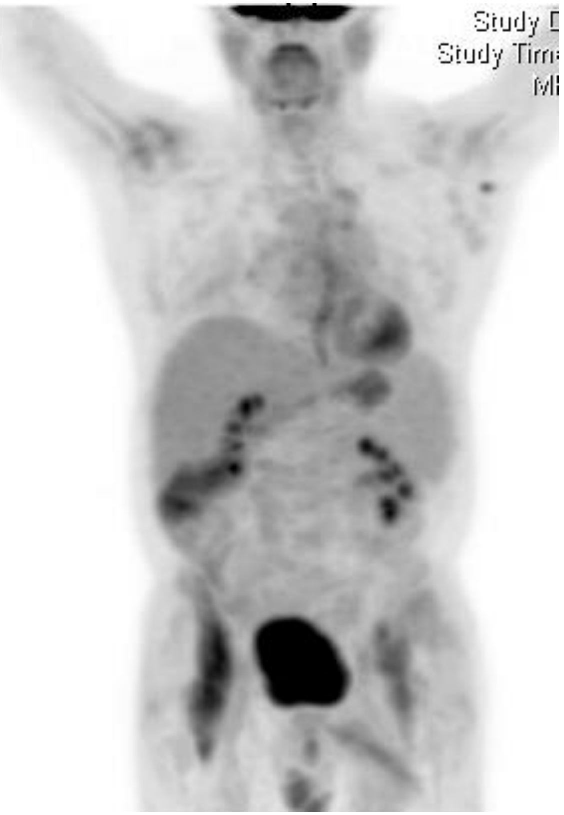
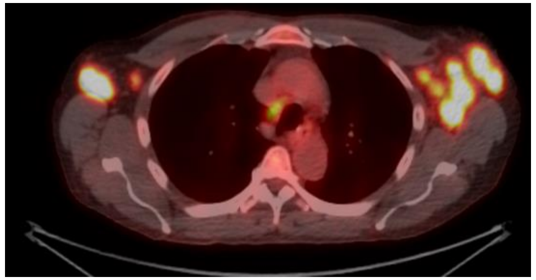
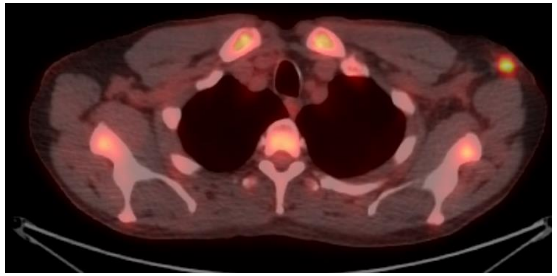

MRN	Age	IPS	Escalated	Cycles	SUV Ratio	Deauville	SUV	Liver	Response	RT	RT resp	Relapse	PFS	Death	OS	Rel prev site	Bx confirmed
	34	0-1	ABVD	6	>=1.3	>=4	8.1	2.6	CR	0		0	29	0	29		

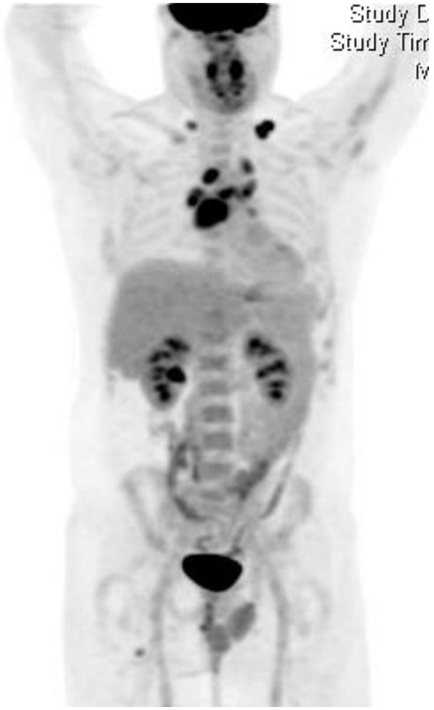

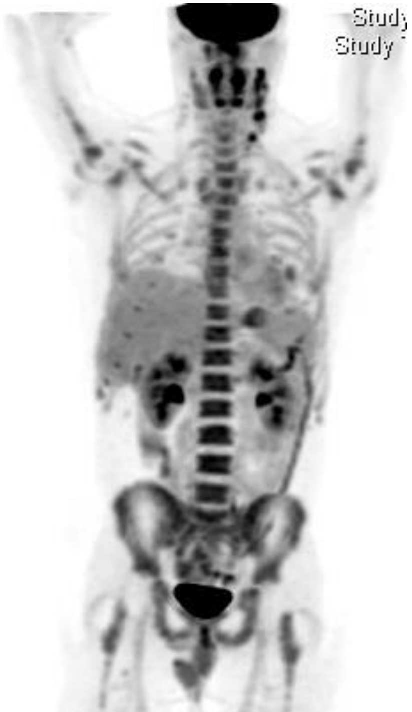

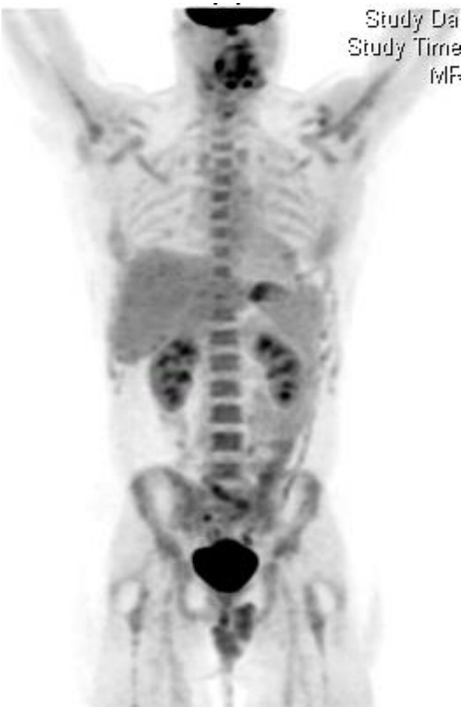

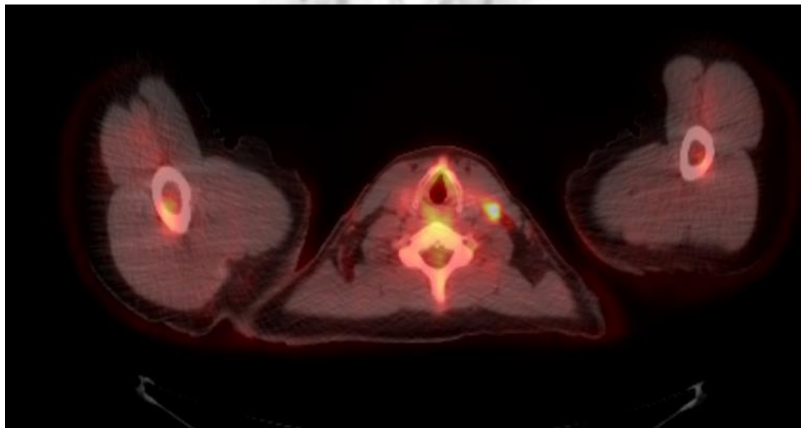

MRN	Age	IPS	Escalated	Cycles	SUV Ratio	Deauville	SUV	Liver	Response	RT	RT resp	Relapse	PFS	Death	OS	Rel prev site	Bx confirmed
	35	2-3	ABVD	6	>=1.3	>=4	9	2.3	PR	0		0	57	0	57		

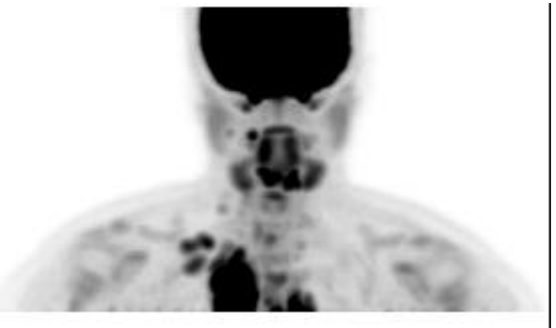

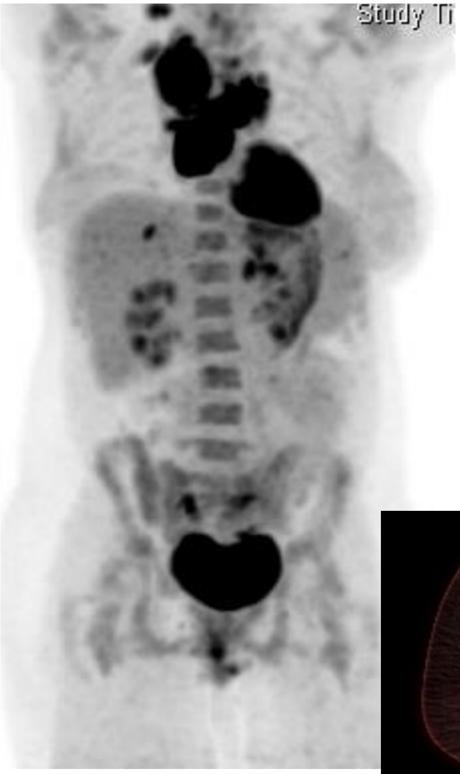

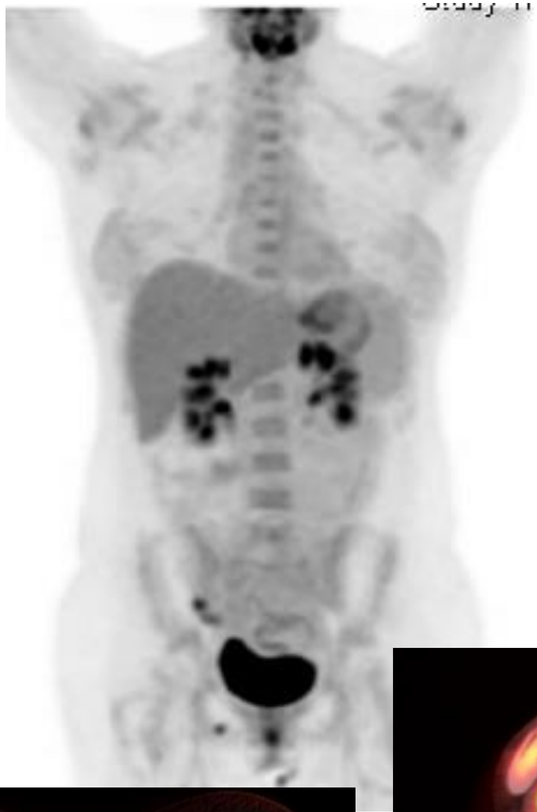

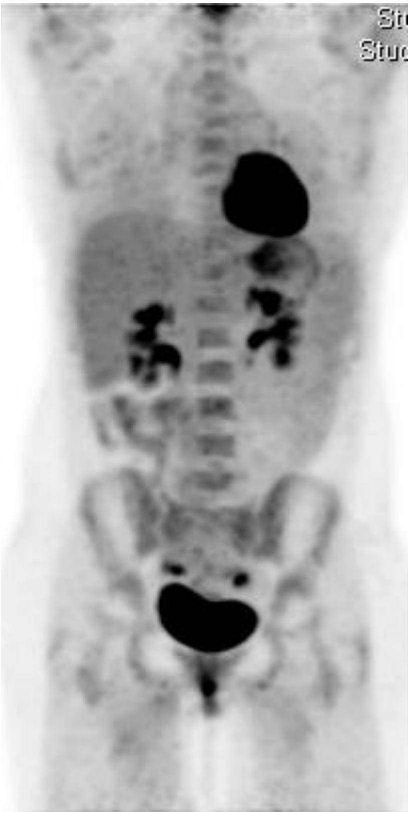

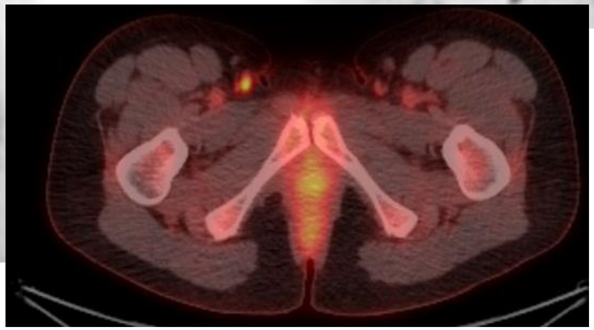

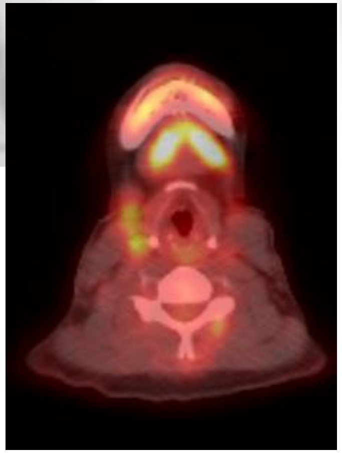

MRN	Age	IPS	Escalated	Cycles	SUV Ratio	Deauville	SUV	Liver	Response	RT	RT resp	Relapse	PFS	Death	OS	Rel prev site	Bx confirmed
	25	2-3	ABVD	6	>=1.3	>=4	2.9	1.8	CR	0		0	42	0	42		

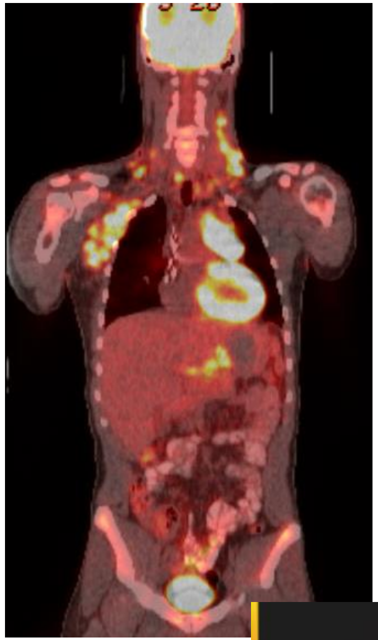

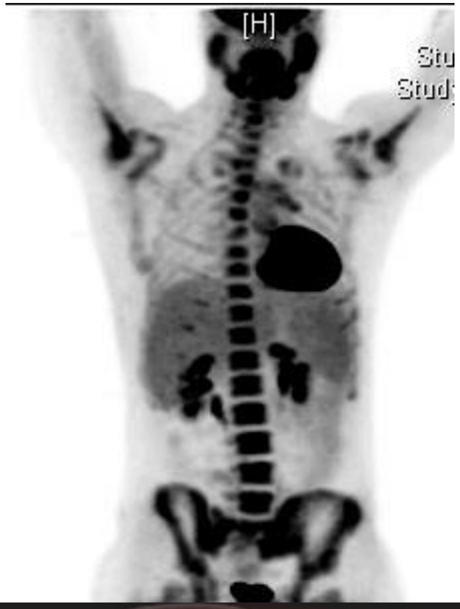

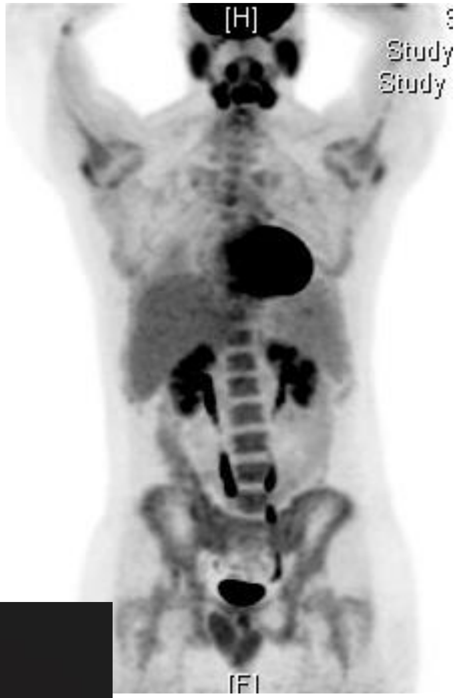

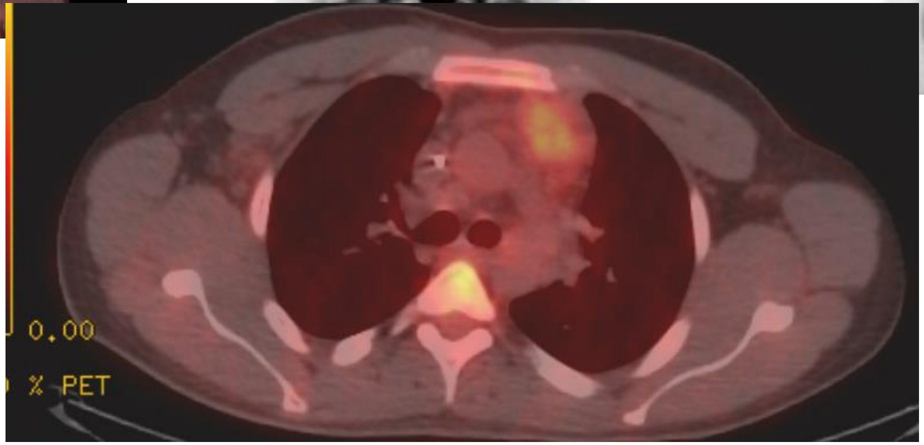

MRN	Age	IPS	Escalated	Cycles	SUV Ratio	Deauville	SUV	Liver	Response	RT	RT resp	Relapse	PFS	Death	OS	Rel prev site	Bx confirmed
	46	2-3	ABVD	6	>=1.3	>=4	4.2	1.9	PR	0		0	64	0	64		

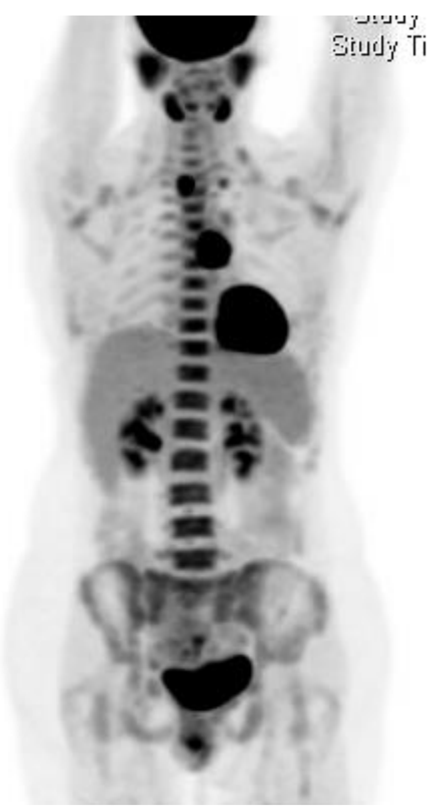

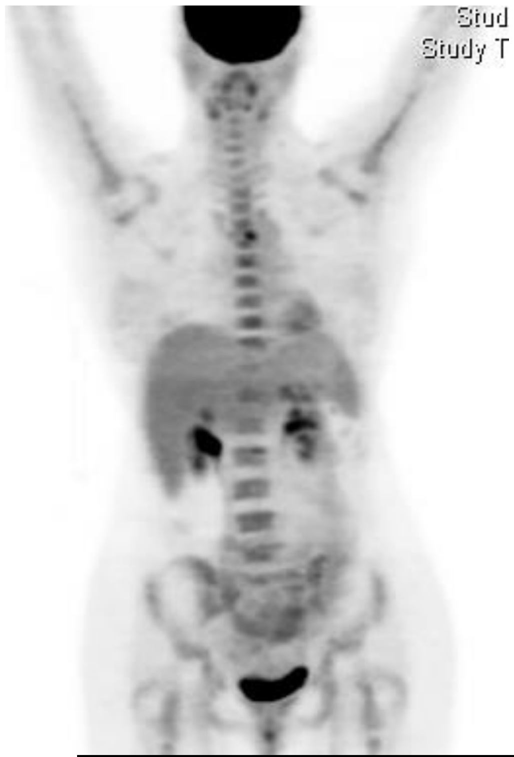

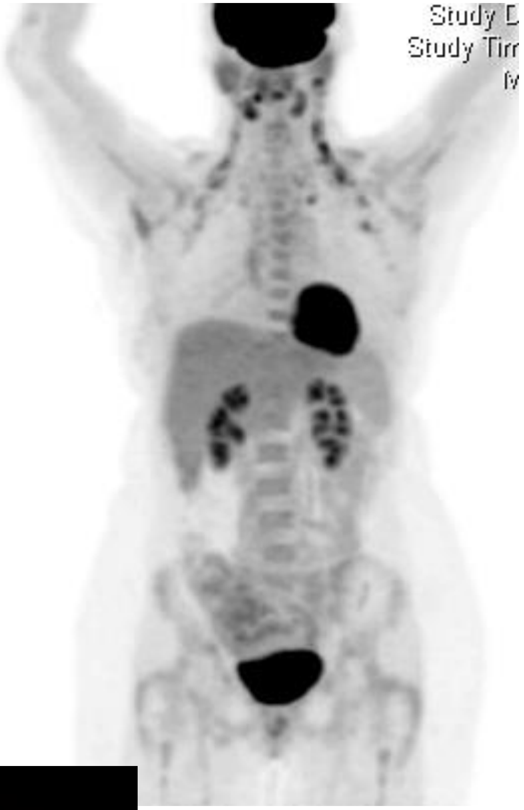

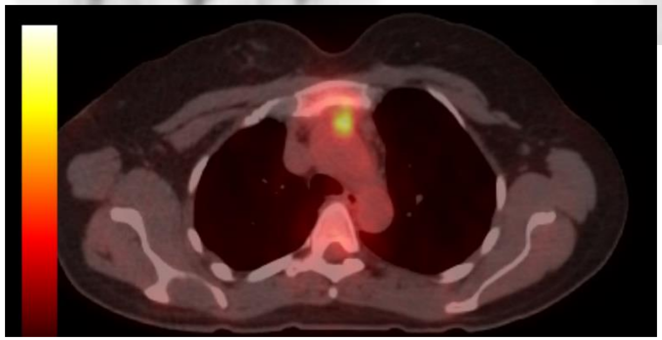

MRN	Age	IPS	Escalated	Cycles	SUV Ratio	Deauville	SUV	Liver	Response	RT	RT resp	Relapse	PFS	Death	OS	Rel prev site	Bx confirmed
	24	2-3	ABVD	6	>=1.3	>=4	8.7	1.6	CR	0		0	25	0	25		

OSH

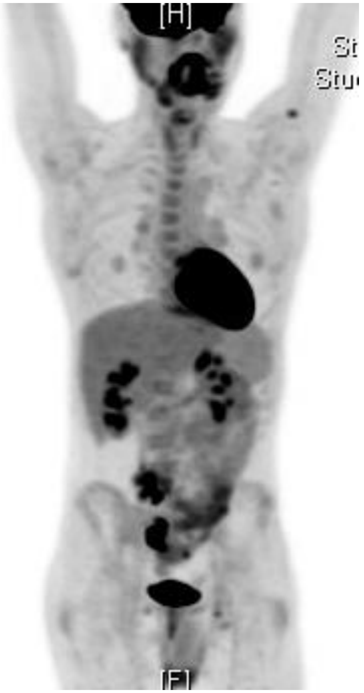

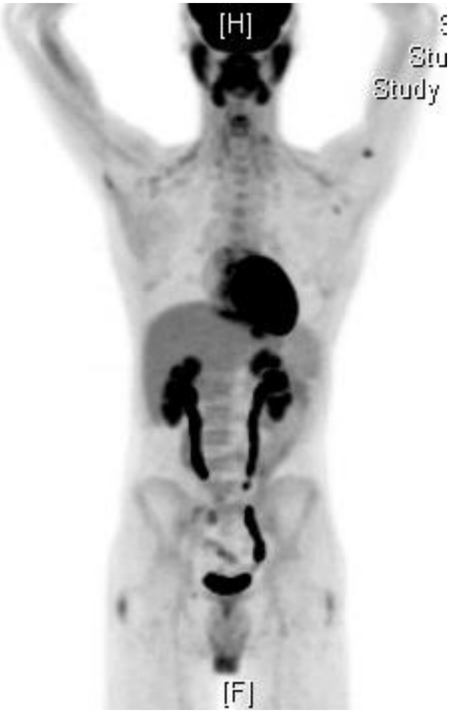

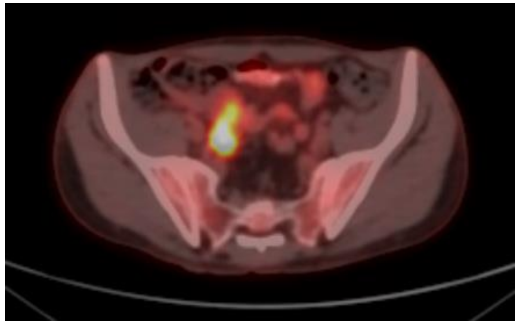

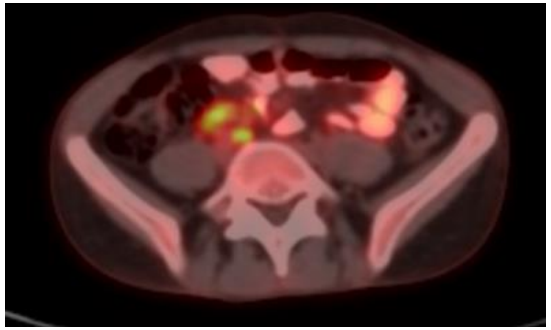

MRN	Age	IPS	Escalated	Cycles	SUV Ratio	Deauville	SUV	Liver	Response	RT	RT resp	Relapse	PFS	Death	OS	Rel prev site	Bx confirmed
	57	4-7	ABVD	6	>=1.3	>=4	3.7	2.3	CR	0		0	54	0	54		

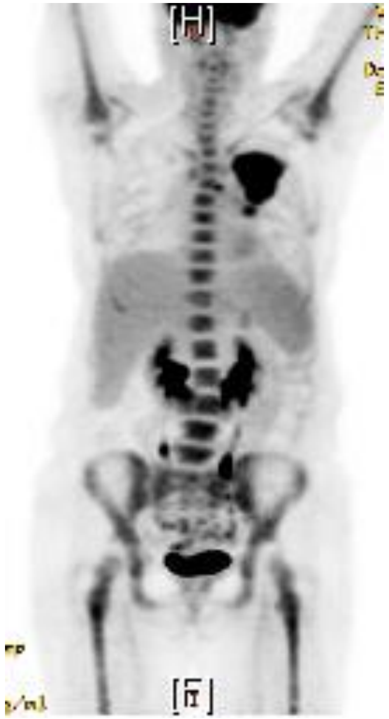

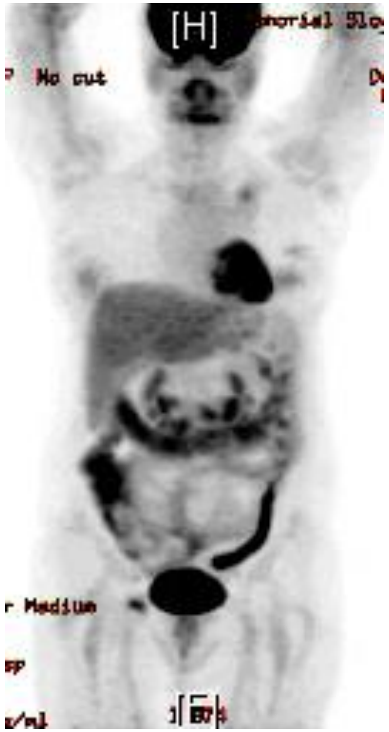

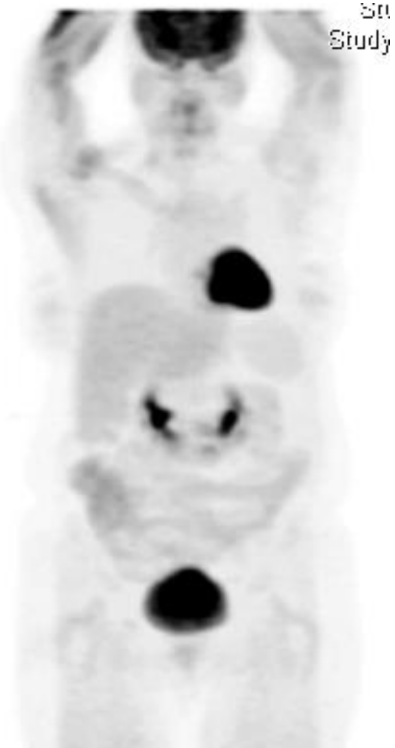

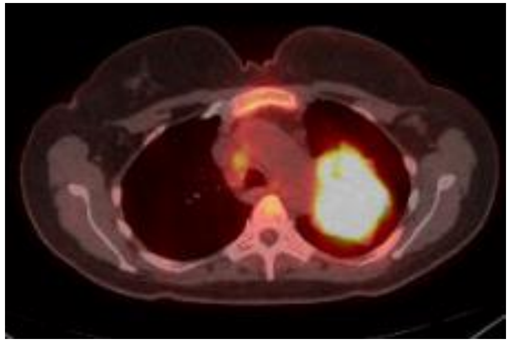

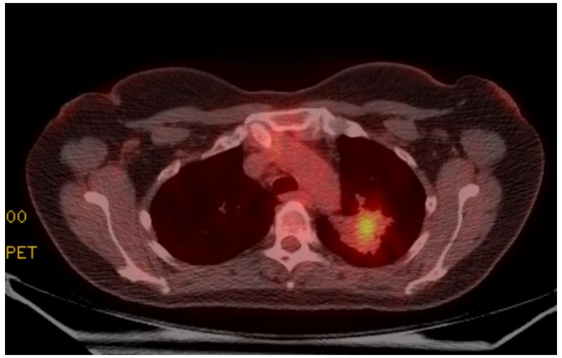

MRN	Age	IPS	Escalated	Cycles	SUV Ratio	Deauville	SUV	Liver	Response	RT	RT resp	Relapse	PFS	Death	OS	Rel prev site	Bx confirmed
	39	0-1	ABVD	6	>=1.3	>=4	4.2	2.9	CR	0		0	61	0	61		

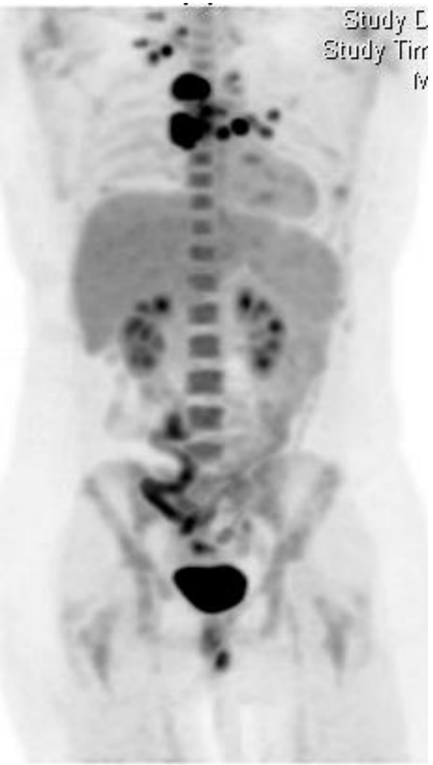

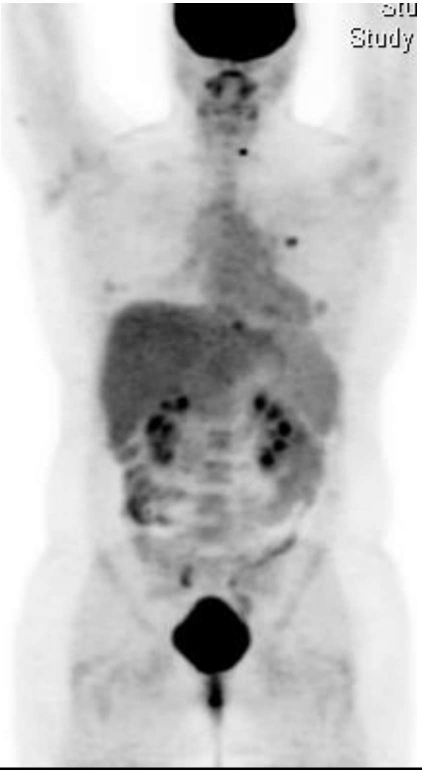

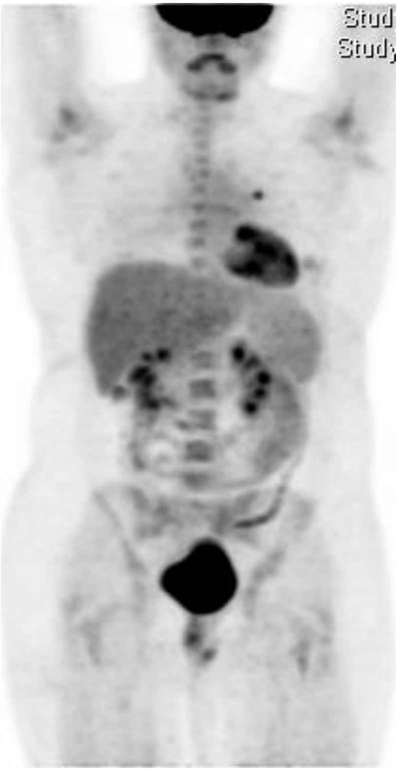

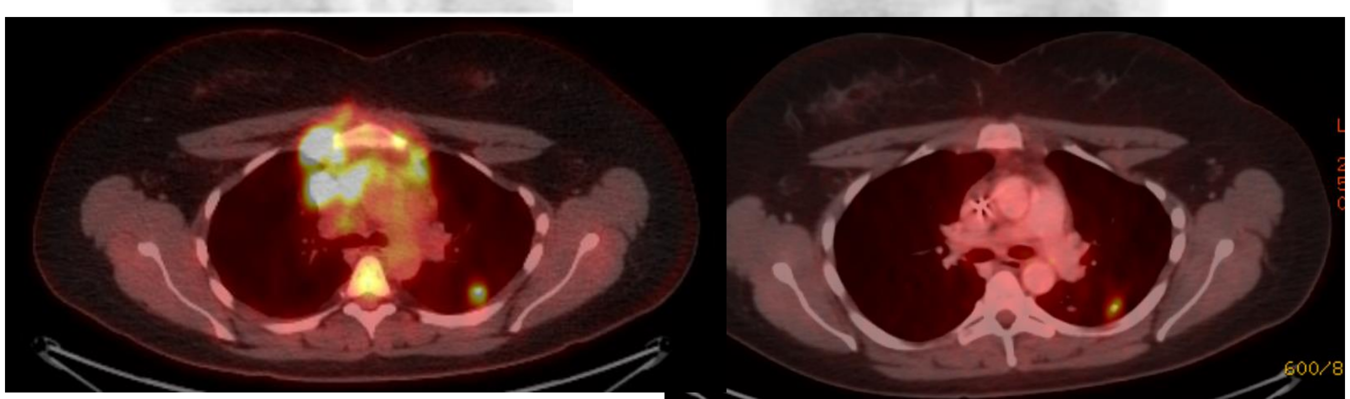

MRN	Age	IPS	Escalated	Cycles	SUV Ratio	Deauville	SUV	Liver	Response	RT	RT resp	Relapse	PFS	Death	OS	Rel prev site	Bx confirmed
	34	0-1	ABVD	6	>=1.3	>=4	4.2	3.32	CR	0		0	30	0	30		

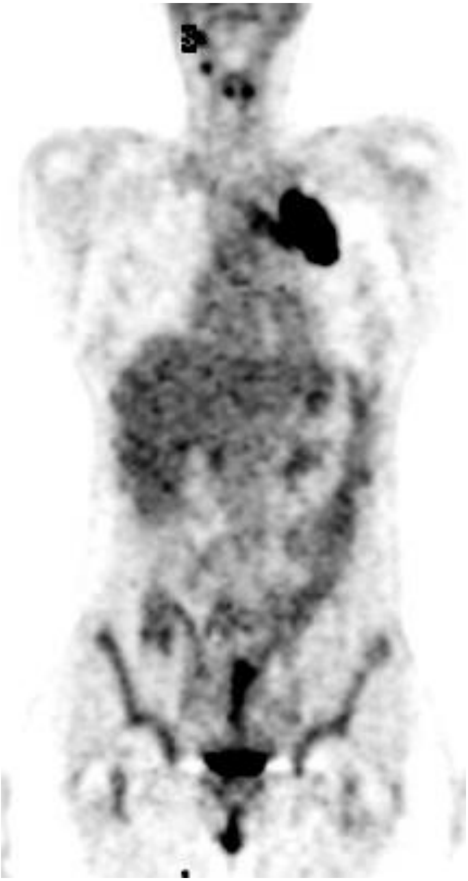

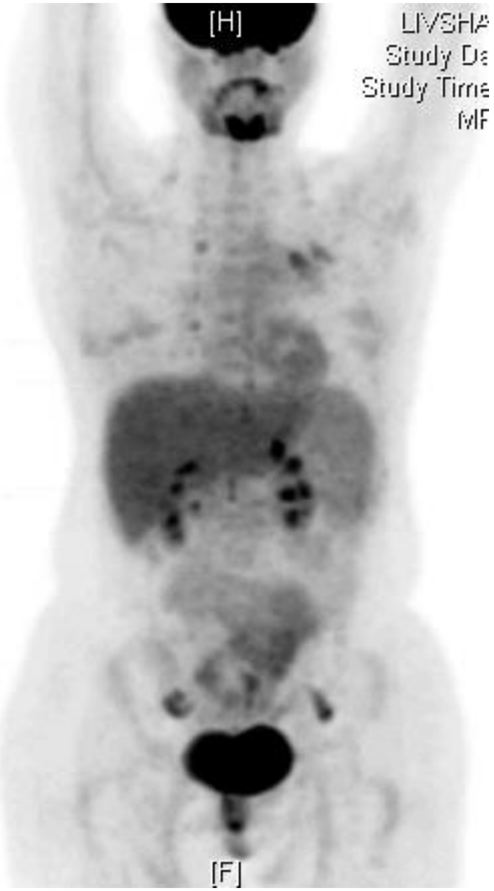

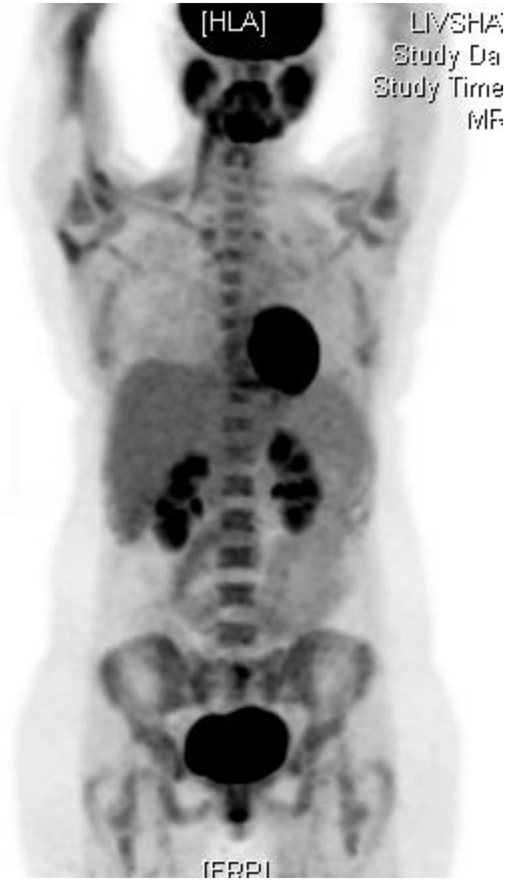

MRN	Age	IPS	Escalated	Cycles	SUV Ratio	Deauville	SUV	Liver	Response	RT	RT resp	Relapse	PFS	Death	OS	Rel prev site	Bx confirmed
	22	2-3	ABVD	6	>=1.3	>=4	3.6	2	CR	0		0	27	0	27		

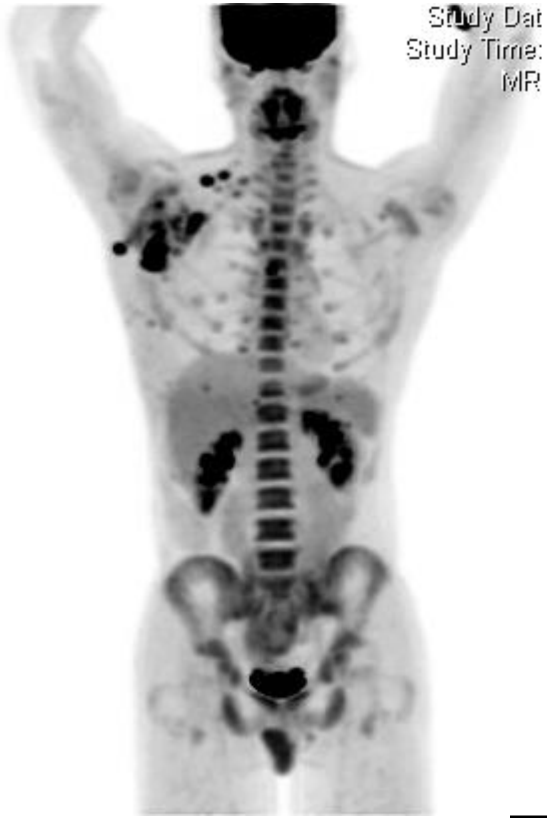

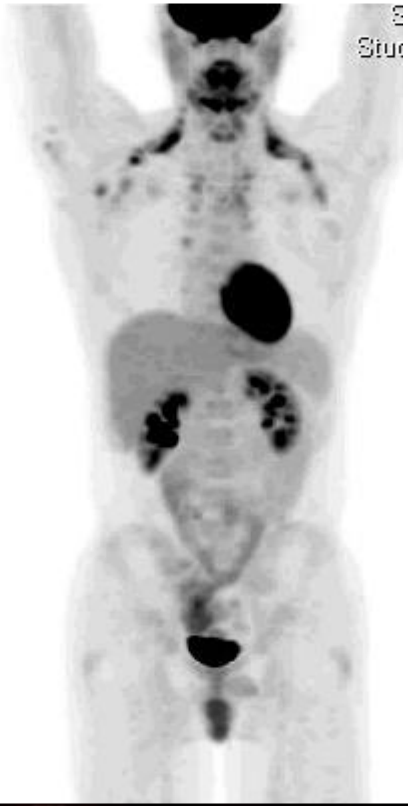

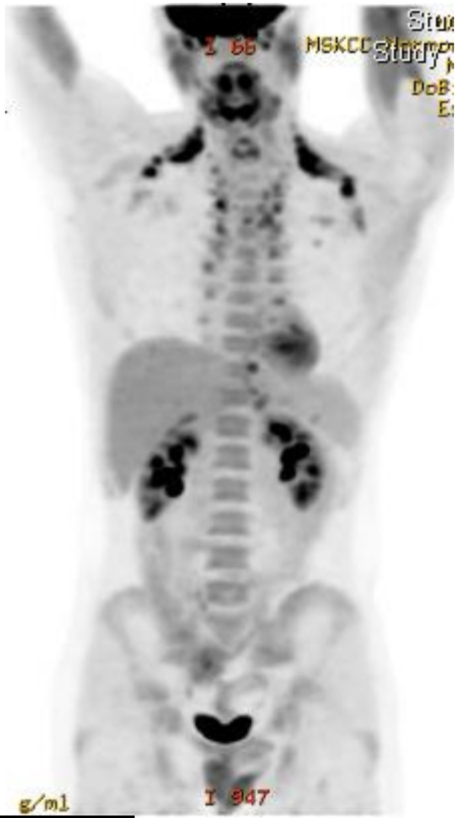

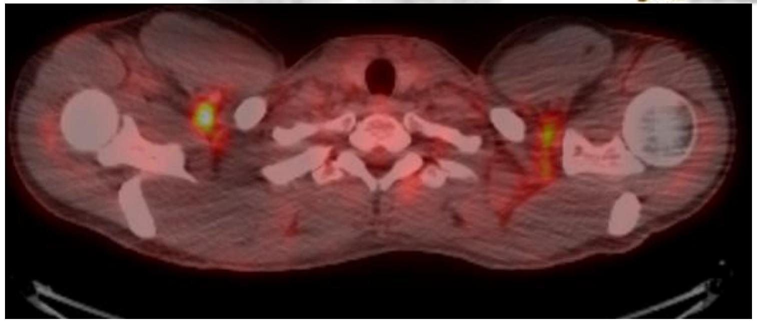

dvPET2+ (Deauville)
Patients with POD

MRN	Age	IPS	Escalated	Cycles	SUV Ratio	Deauville	SUV	Liver	Response	RT	RT resp	Relapse	PFS	Death	OS	Rel prev site	Bx confirmed
	37	2-3	ABVD	4	>=1.3	>=4	7.2	3.4	PR	0		1	4	0	39	1	1

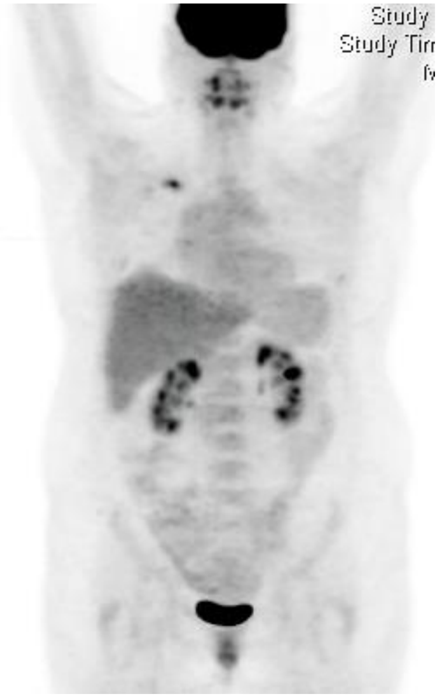

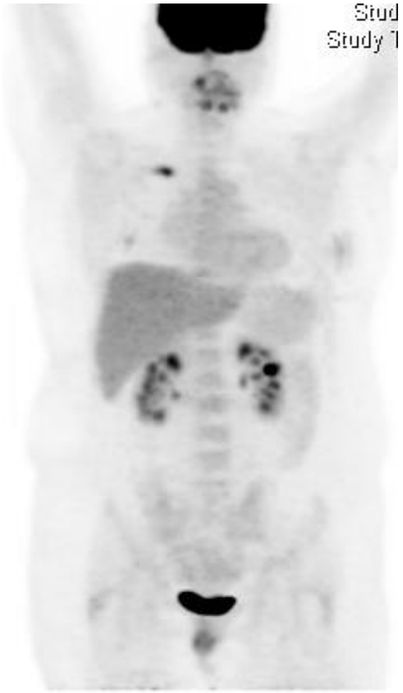

Tx 2
BV+nivolumab - ASCT

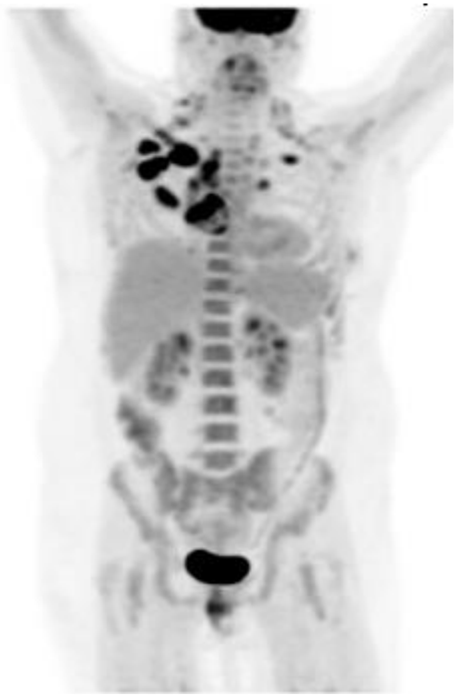

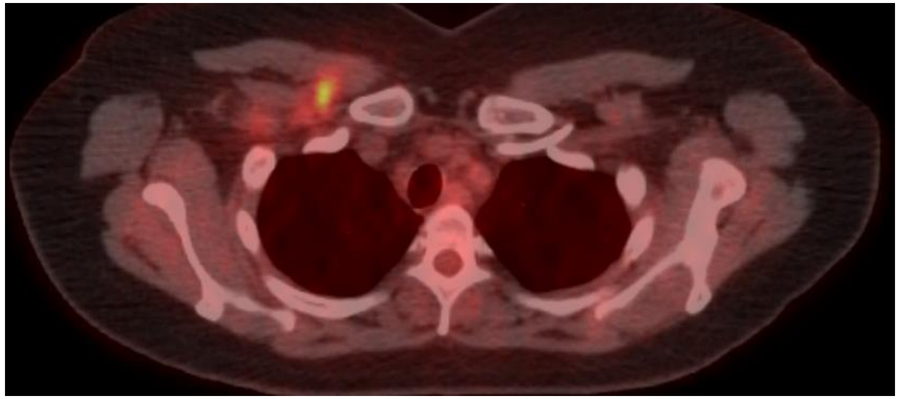

MRN	Age	IPS	Escalated	Cycles	SUV Ratio	Deauville	SUV	Liver	Response	RT	RT resp	Relapse	PFS	Death	OS	Rel prev site	Bx confirmed
	36	0-1	ABVD	6	>=1.3	>=4	4.3	1.6	POD	0		1	6	1	79	1	1

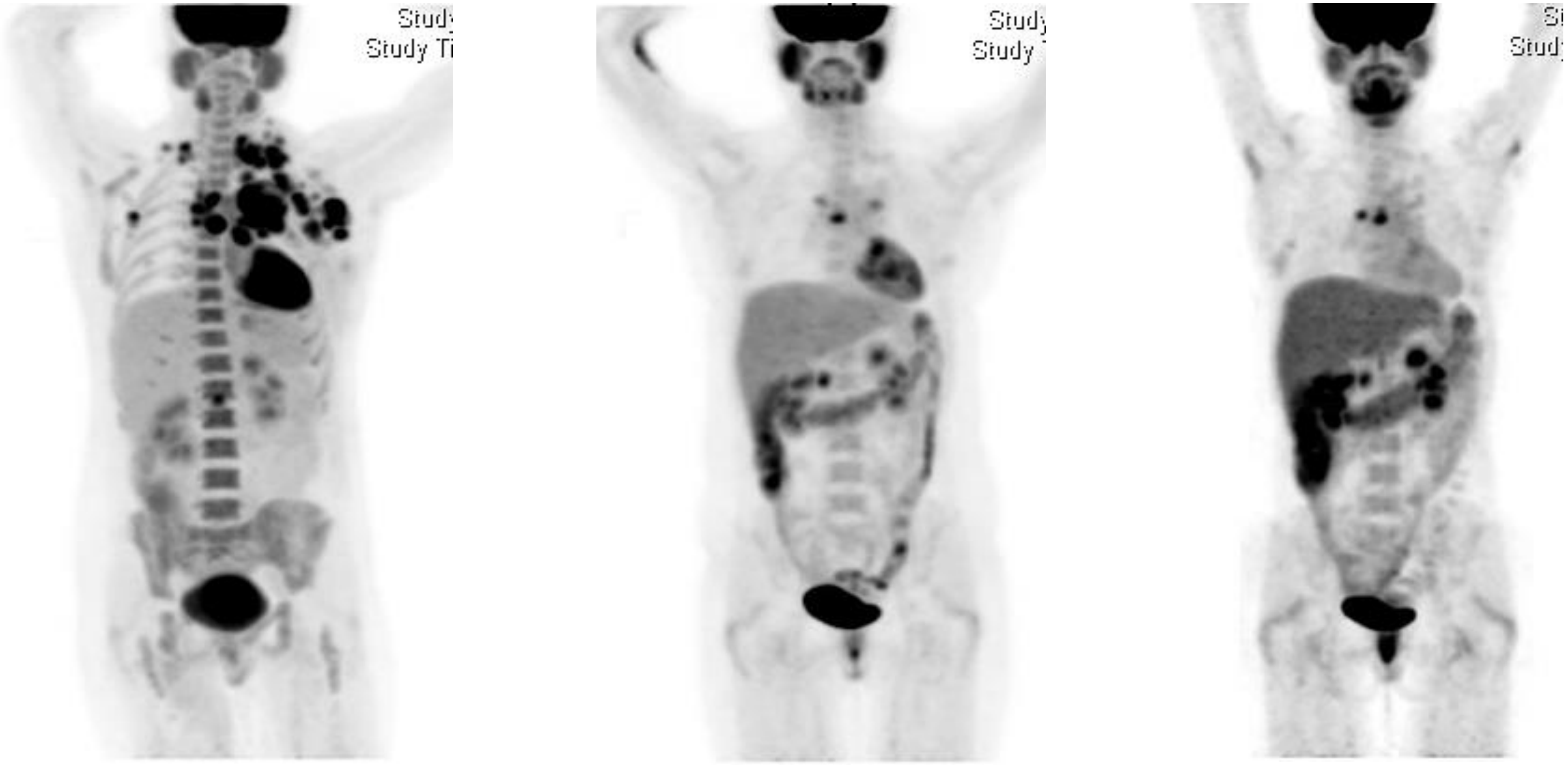

Tx-2
BV-ICE-ASCT

MRN	Age	IPS	Escalated	Cycles	SUV Ratio	Deauville	SUV	Liver	Response	RT	RT resp	Relapse	PFS	Death	OS	Rel prev site	Bx confirmed
	57	0-1	ABVD	6	>=1.3	DV-4	3.7	2.29	POD	0		1	6	0	28	1	1

Tx-2
BV-ASCT

OSH

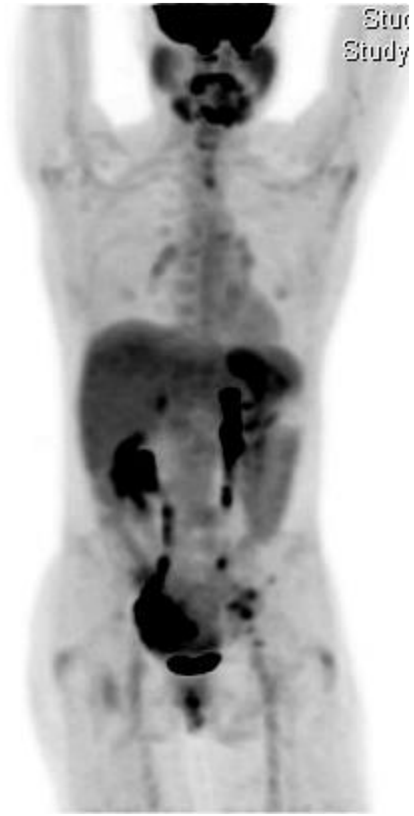

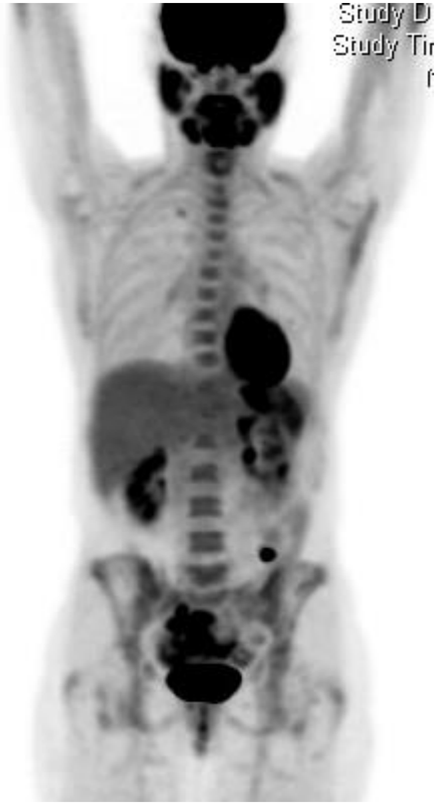

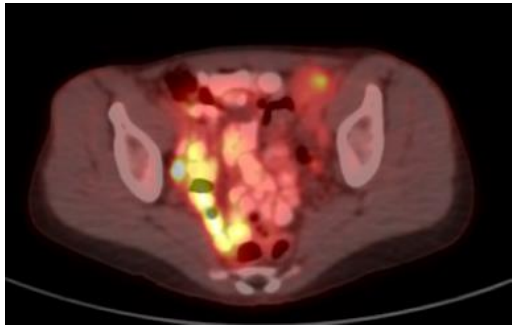

MRN	Age	IPS	Escalated	Cycles	SUV Ratio	Deauville	SUV	Liver	Response	RT	RT resp	Relapse	PFS	Death	OS	Rel prev site	Bx confirmed
	31	0-1	ABVD	6	>=1.3	>=4	6.9	3.04	POD	0		1	6	1	102	1	1

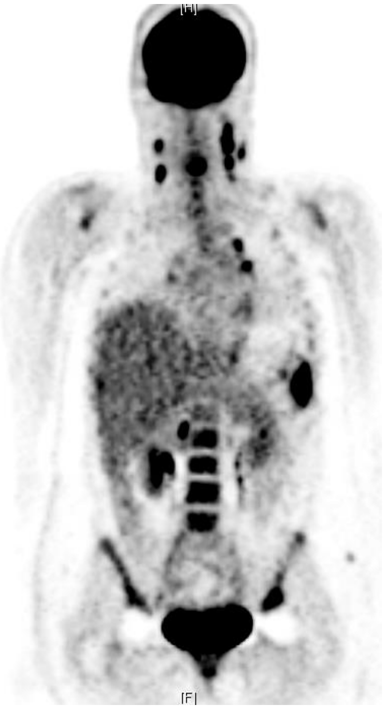

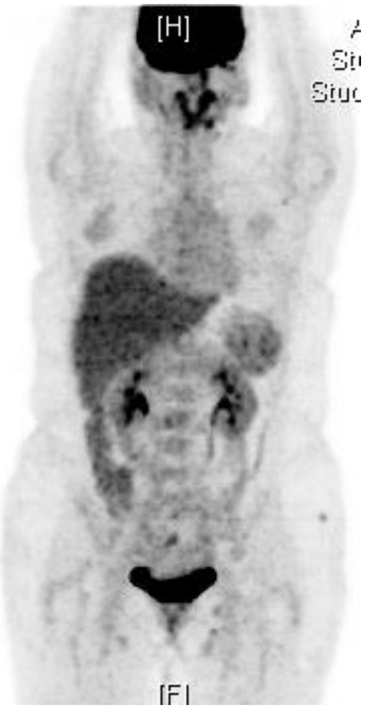

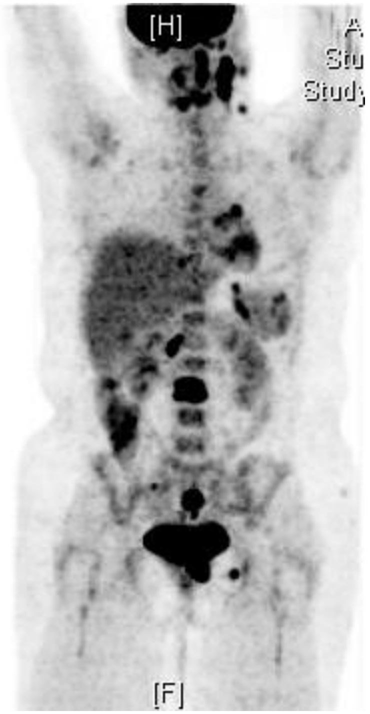

Tx-2
IVG + allo SCT

MRN	Age	IPS	Escalated	Cycles	SUV Ratio	Deauville	SUV	Liver	Response	RT	RT resp	Relapse	PFS	Death	OS	Rel prev site	Bx confirmed
	22	0-1	ABVD	4	>=1.3	>=4	4	1.7	PR	0		1	4	0	54	1	1

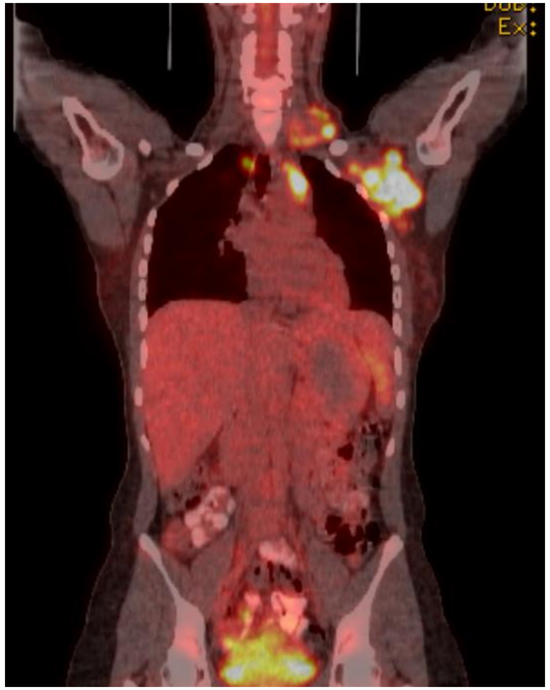

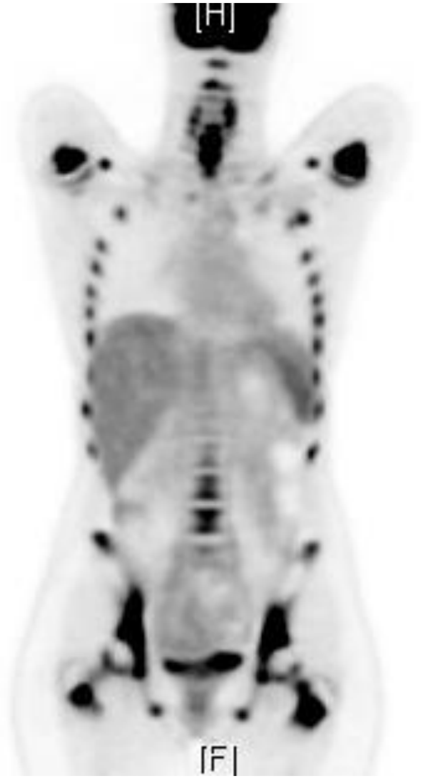

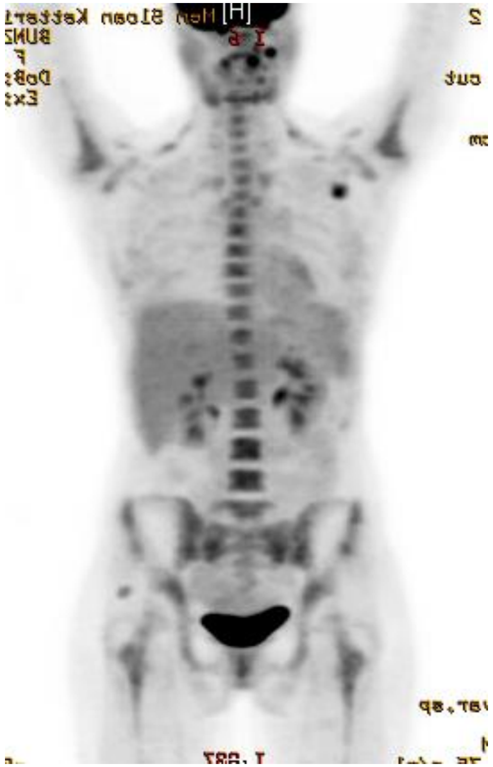

Tx-2
BV-ICE-ASCT

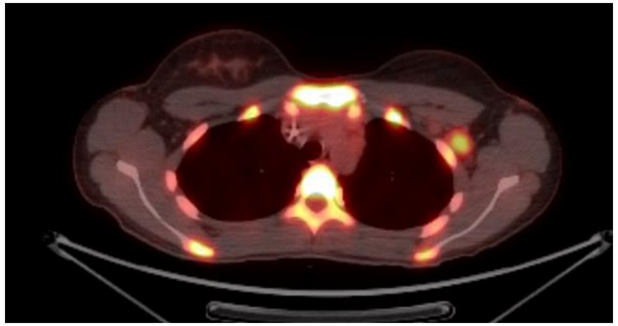

MRN	Age	IPS	Escalated	Cycles	SUV Ratio	Deauville	SUV	Liver	Response	RT	RT resp	Relapse	PFS	Death	OS	Rel prev site	Bx confirmed
	65	4-7	ABVD	6	>=1.3	>=4	3.7	2.55	CR	0		1	25	1	86	1	1

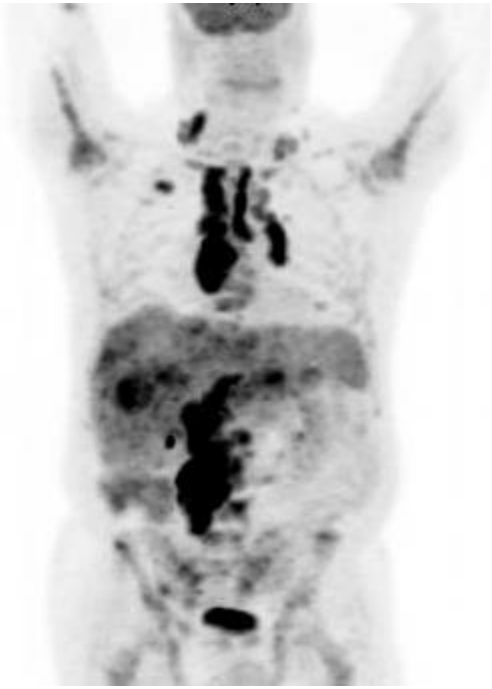

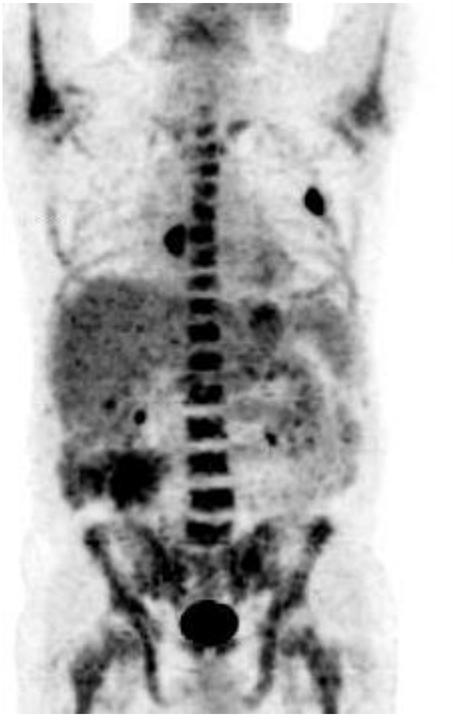

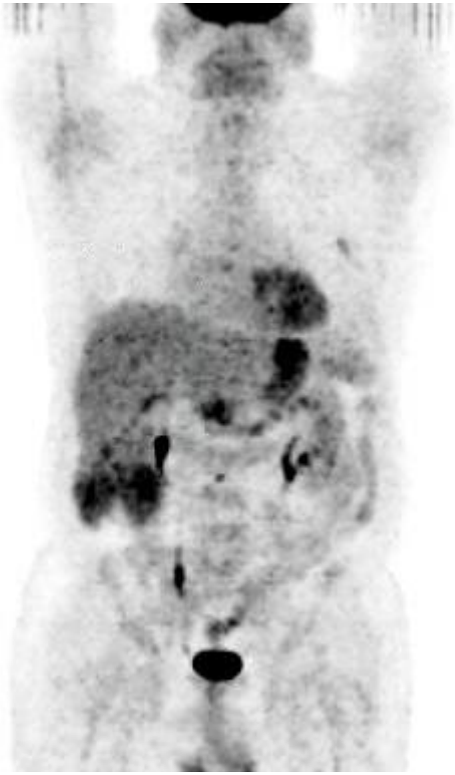

Note: relapse after 25m

Tx-2
ICE-ASCT

MRN	Age	IPS	Escalated	Cycles	SUV Ratio	Deauville	SUV	Liver	Response	RT	RT resp	Relapse	PFS	Death	OS	Rel prev site	Bx confirmed
	32	4-7	ABVD	6	>=1.3	>=4	3.7	2.5	CR	0		1	22	0	54	1	1

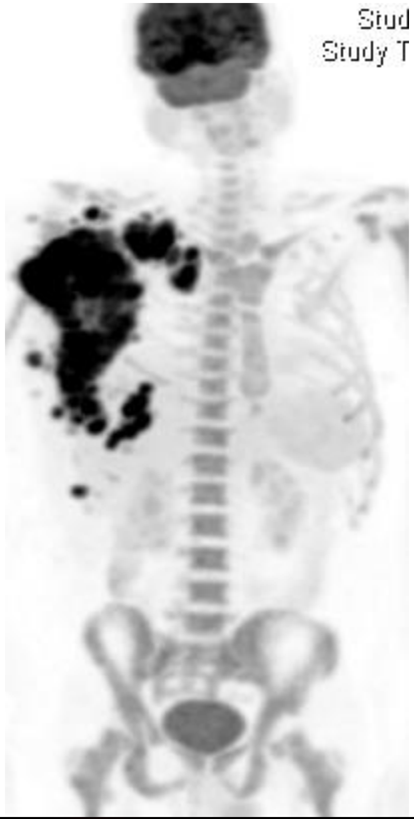

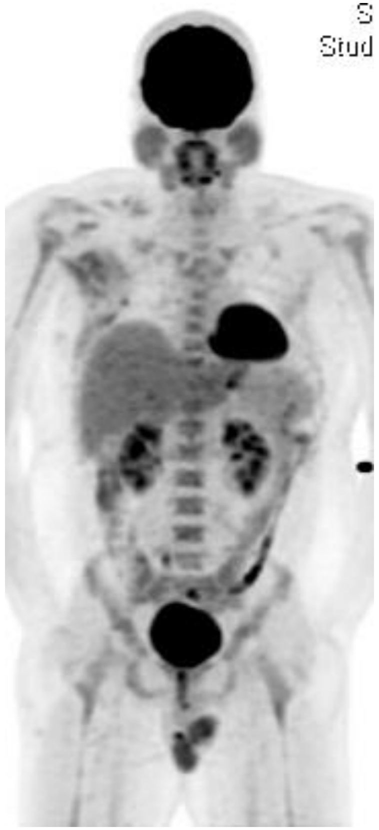

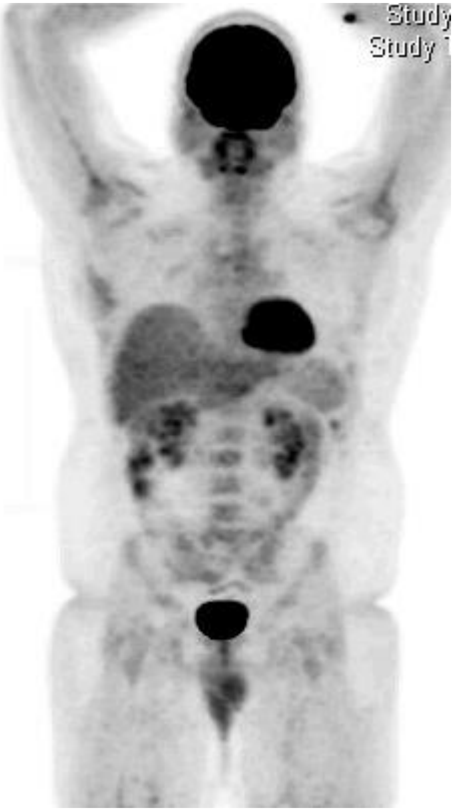

Note: relapse after 22m

Tx-2
BV-ASCT

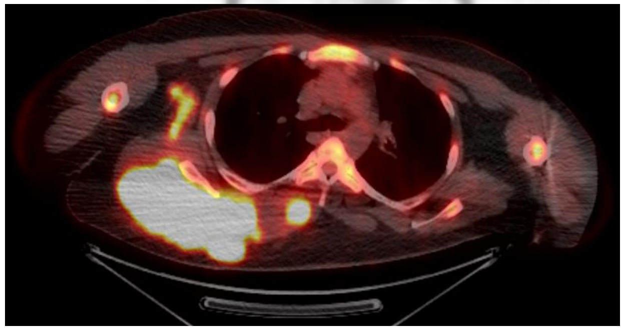

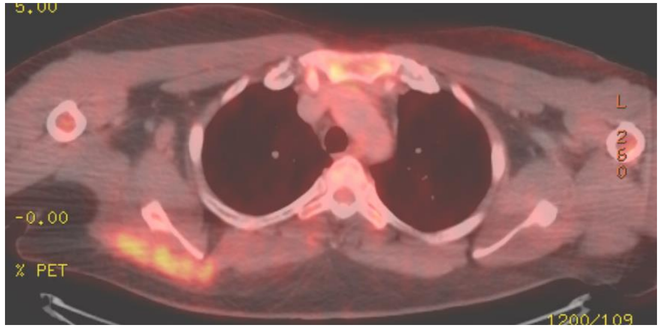

MRN	Age	IPS	Escalated	Cycles	SUV Ratio	Deauville	SUV	Liver	Response	RT	RT resp	Relapse	PFS	Death	OS	Rel prev site	Bx confirmed
	22	0-1	ABVD	6	>=1.3	>=4	3.7	2	POD	0		1	6	0	76	1	1

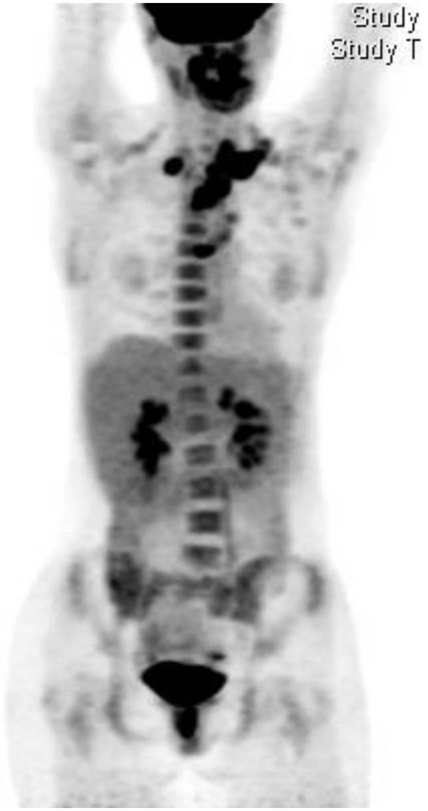

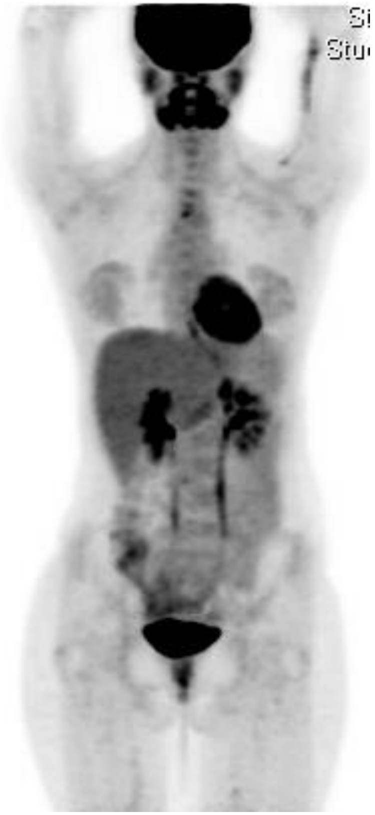

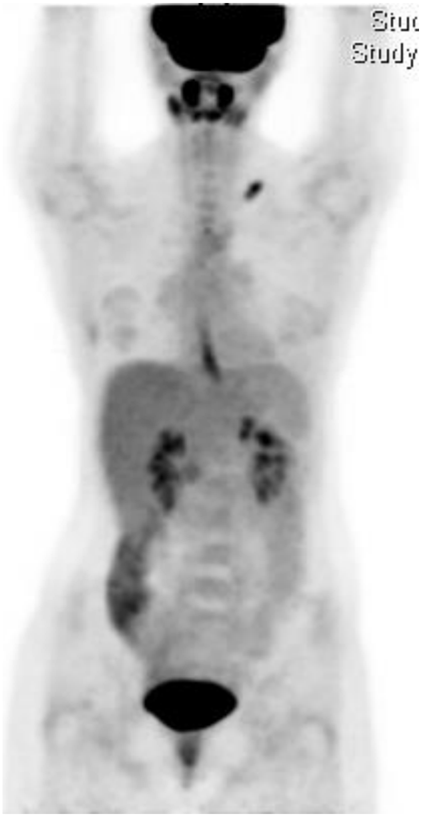

Tx-2
BV-ICE-ASCT

qPET2+ by LLS only (dvPET2 -)
Patients with POD

MRN	Age	IPS	Escalated	Cycles	SUV Ratio	Deauville	SUV	Liver	Response	RT	RT resp	Relapse	PFS	Death	OS	Rel prev site	Bx confirmed
	20	2-3	ABVD	6	>=1.3	DV-1-3	2.7	1.8	POD	0		1	6	0	66	1	1

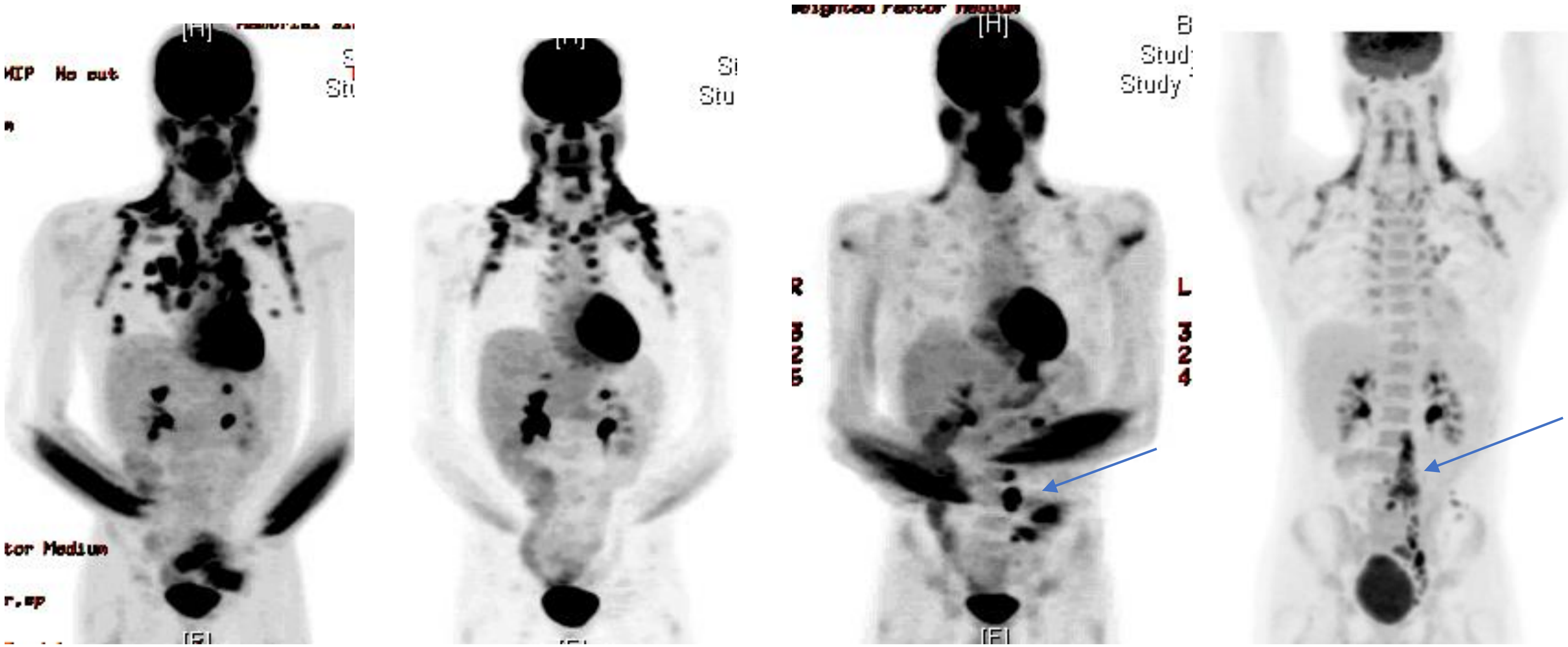

Tx-2
BV-ICE-ASCT

MRN	Age	IPS	Escalated	Cycles	SUV Ratio	Deauville	SUV	Liver	Response	RT	RT resp	Relapse	PFS	Death	OS	Rel prev site	Bx confirmed
	34	4-7	ABVD	6	>=1.3	DV-1-3	2.3	1.8	POD	0		1	10	0	27	1	1

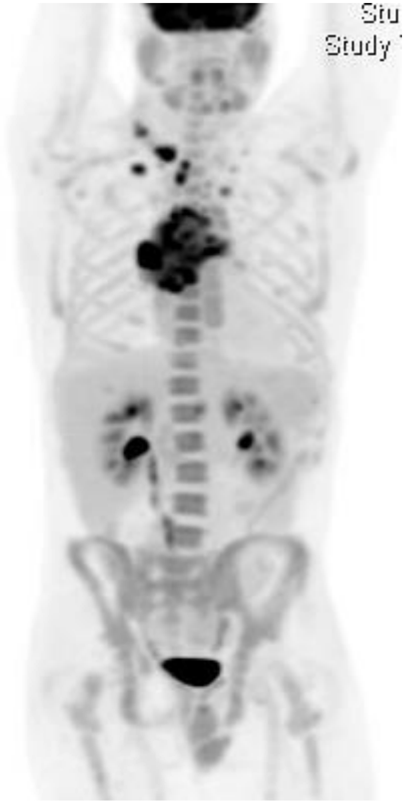

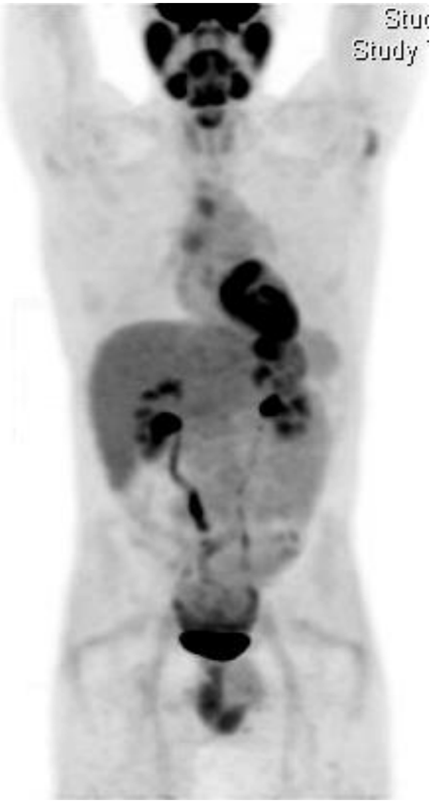

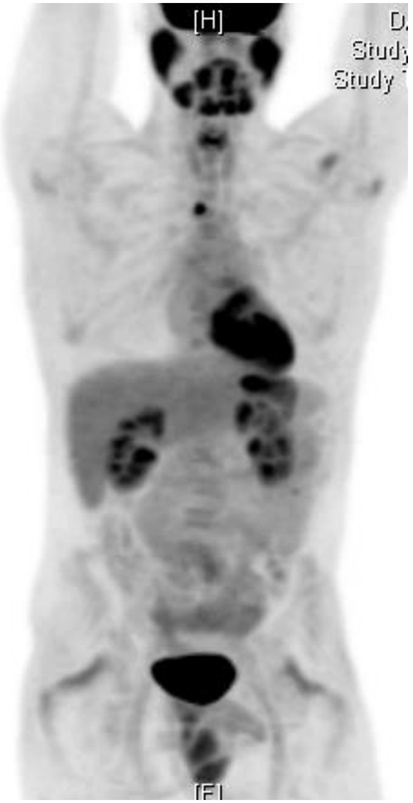

Tx-2
ICE-ASCT

MRN	Age	IPS	Escalated	Cycles	SUV Ratio	Deauville	SUV	Liver	Response	RT	RT resp	Relapse	PFS	Death	OS	Rel prev site	Bx confirmed
	31	0-1	ABVD	6	>=1.3	DV-1-3	2.8	1.9	POD	0		1	9	0	47	1	1

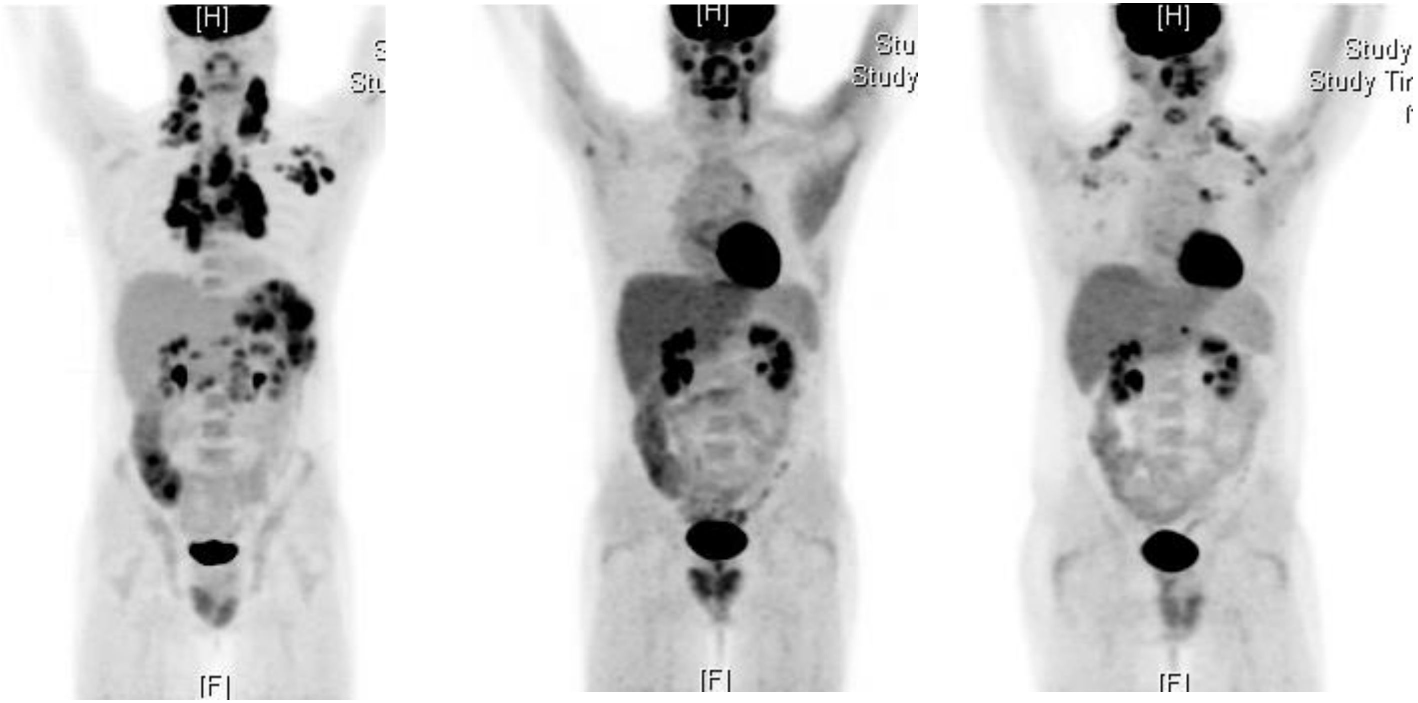

Tx-2
ICE-ASCT

MRN	Age	IPS	Escalated	Cycles	SUV Ratio	Deauville	SUV	Liver	Response	RT	RT resp	Relapse	PFS	Death	OS	Rel prev site	Bx confirmed
	36	0-1	ABVD	6	>=1.3	DV-1-3	3.3	2.5	PR	1	CR	1	16	0	51	1	1

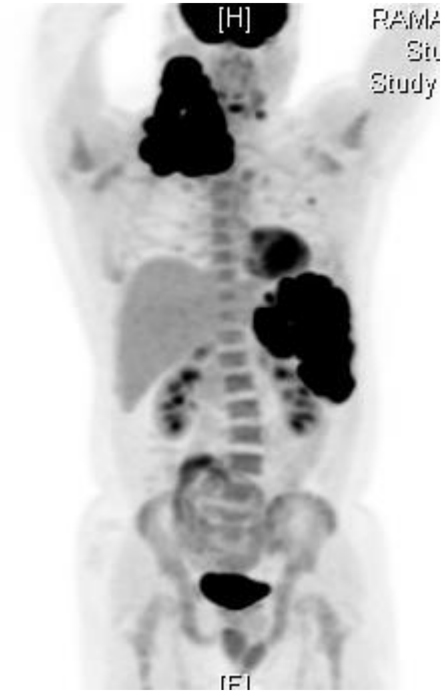

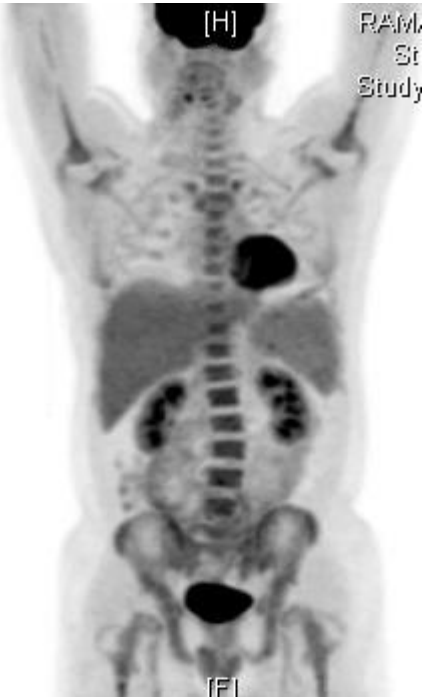

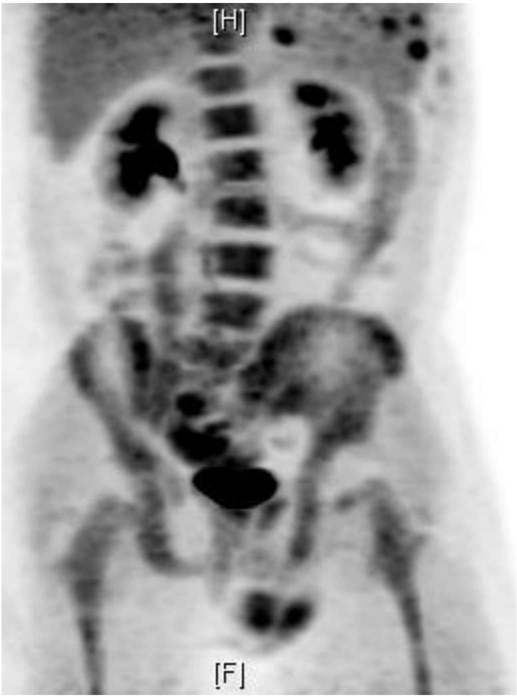

Tx 2
BV+nivolumab - ASCT

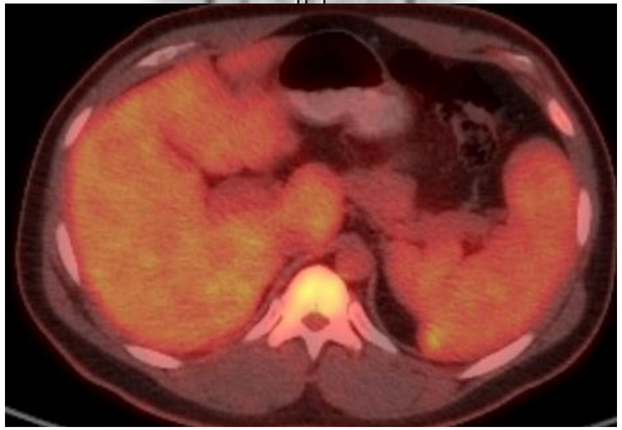

Residual mild tiny focal areas of hypermetabolism in the spleen with maximum SUV 3.3 compared to 26.

MRN	Age	IPS	Escalated	Cycles	SUV Ratio	Deauville	SUV	Liver	Response	RT	RT resp	Relapse	PFS	Death	OS	Rel prev site	Bx confirmed
	32	0-1	ABVD	6	>=1.3	DV-1-3	3.8	2.4	PR	0		1	9	0	91	1	1

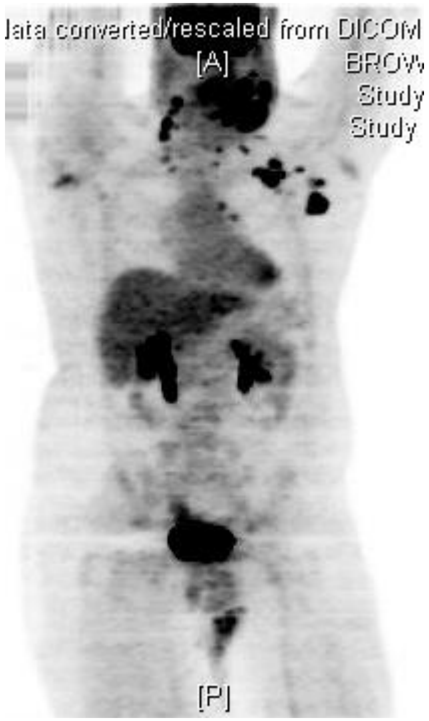

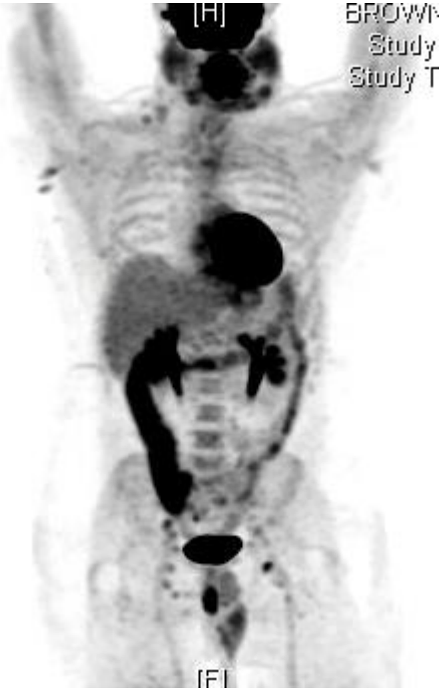

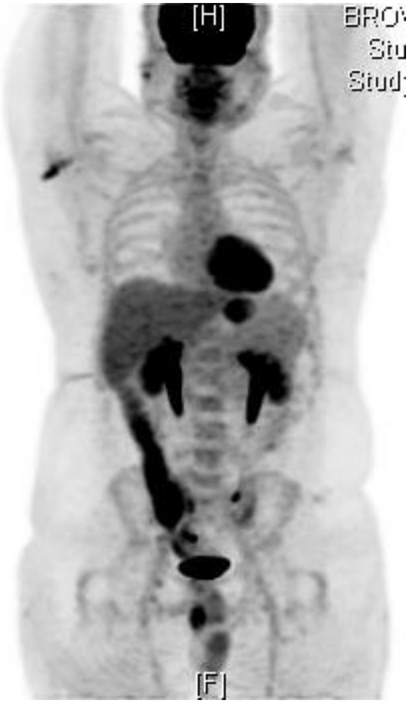

Tx 2
R-da-EPOCH

MRN	Age	IPS	Escalated	Cycles	SUV Ratio	Deauville	SUV	Liver	Response	RT	RT resp	Relapse	PFS	Death	OS	Rel prev site	Bx confirmed
	43	2-3	ABVD	6	>=1.3	DV-1-3	2.2	1.68	PR	0		1	49	0	88	1	1

Tx-2
ICE-ASCT

OSH

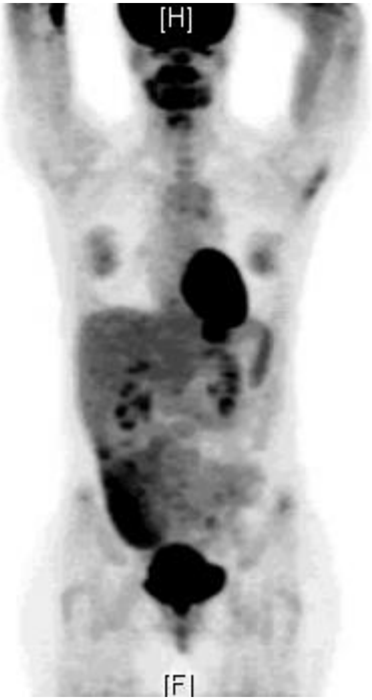

Interim only

EOT - CT

MRN	Age	IPS	Escalated	Cycles	SUV Ratio	Deauville	SUV	Liver	Response	RT	RT resp	Relapse	PFS	Death	OS	Rel prev site	Bx confirmed
	64	4-7	ABVD	6	>=1.3	DV-1-3	3.53	2.9	TRM	0		0	6	1	6		

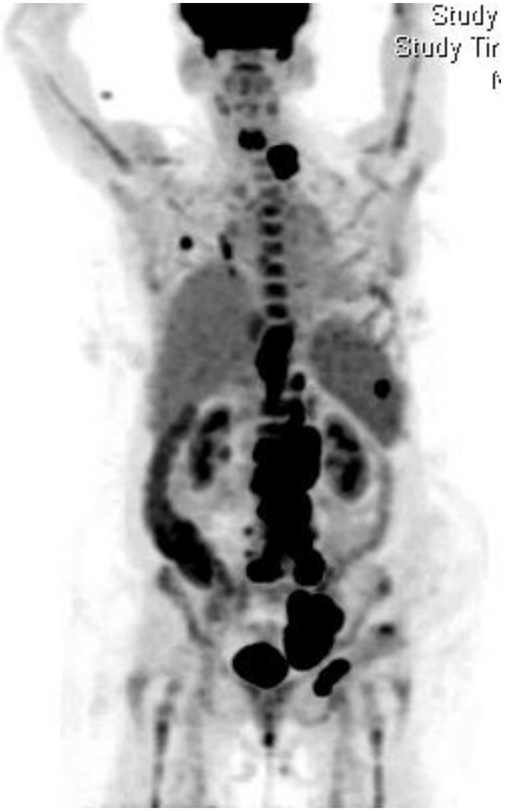

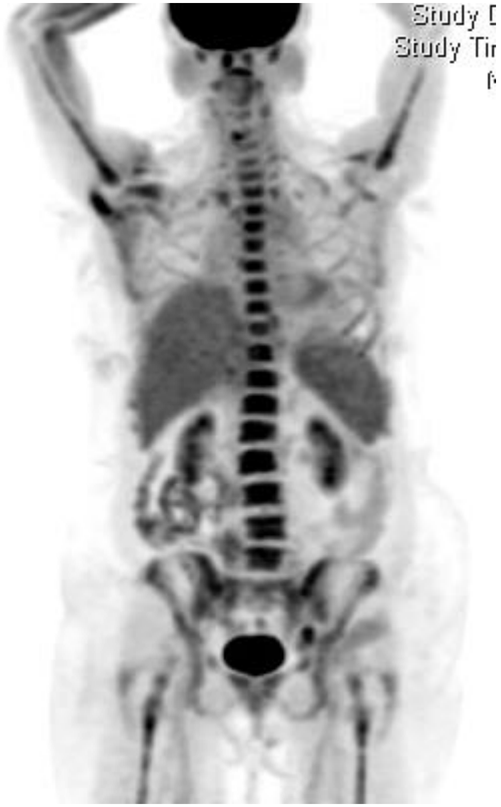

TRM

qPET2+ by LLS only (dvPET2-)
Patients with long term remission

MRN	Age	IPS	Escalated	Cycles	SUV Ratio	Deauville	SUV	Liver	Response	RT	RT resp	Relapse	PFS	Death	OS	Rel prev site	Bx confirmed
	87	2-3	ABVD	6	>=1.3	DV-1-3	4.5	2.56	CR	0		0	11	0	11		

OSH

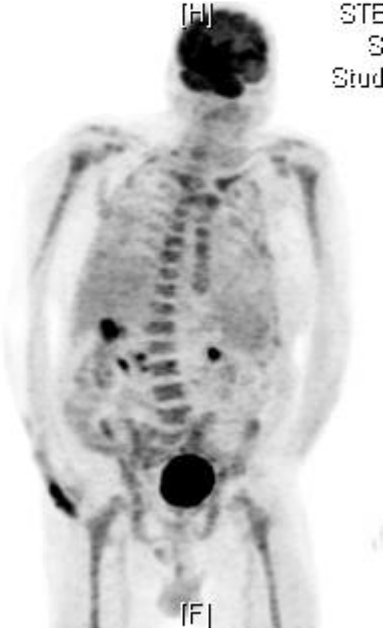

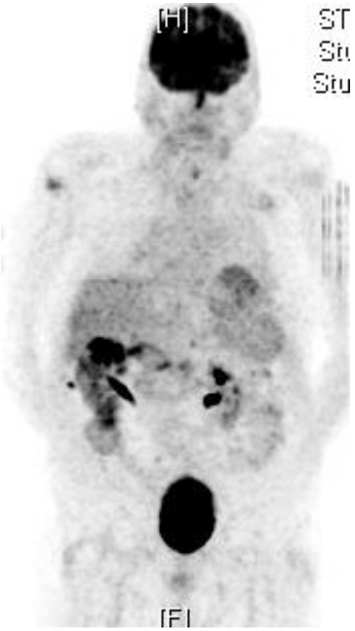

MRN	Age	IPS	Escalated	Cycles	SUV Ratio	Deauville	SUV	Liver	Response	RT	RT resp	Relapse	PFS	Death	OS	Rel prev site	Bx confirmed
	56	2-3	ABVD	6	>=1.3	DV-1-3	2.9	2.2	CR	0		0	83	0	83		

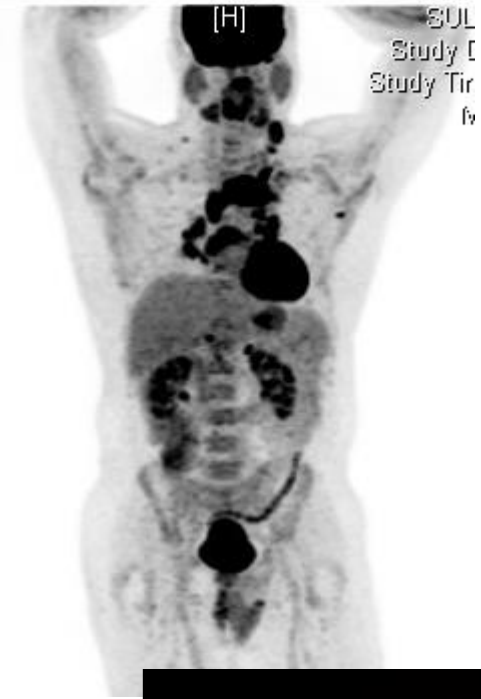

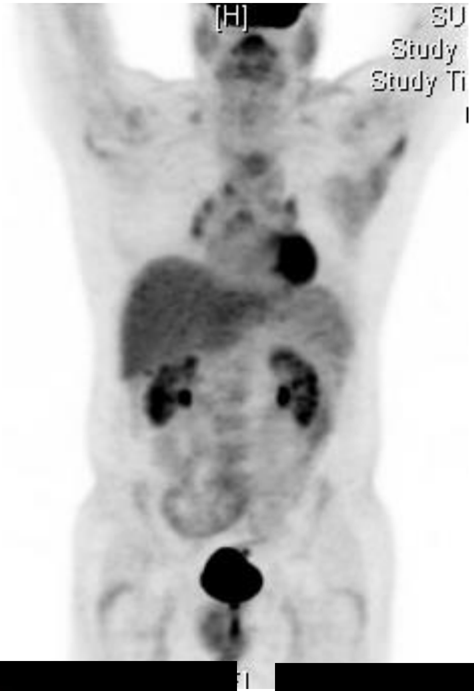

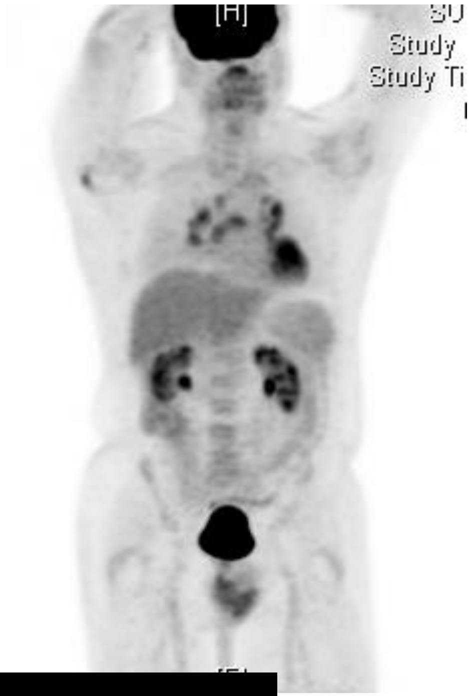

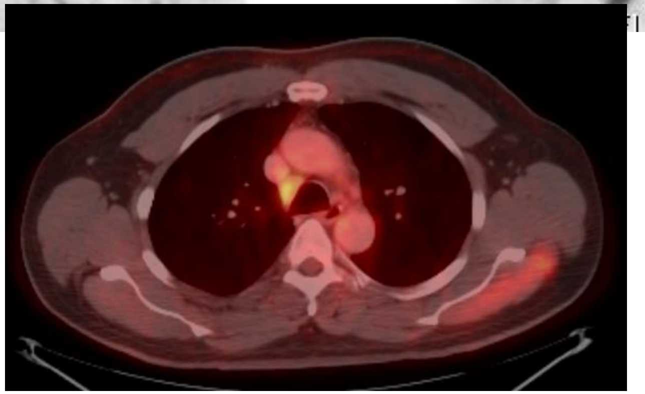

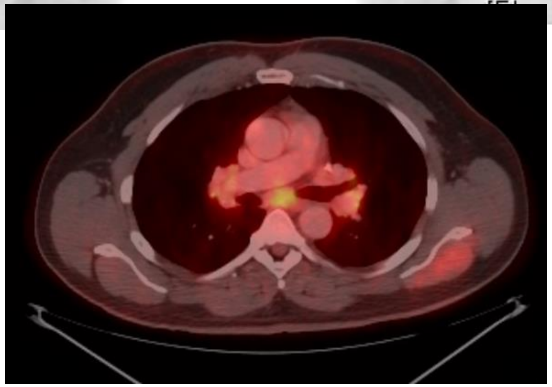

MRN	Age	IPS	Escalated	Cycles	SUV Ratio	Deauville	SUV	Liver	Response	RT	RT resp	Relapse	PFS	Death	OS	Rel prev site	Bx confirmed
	19	0-1	ABVD	6	>=1.3	DV-1-3	2.8	1.6	CR	0		0	51	0	51		

OSH

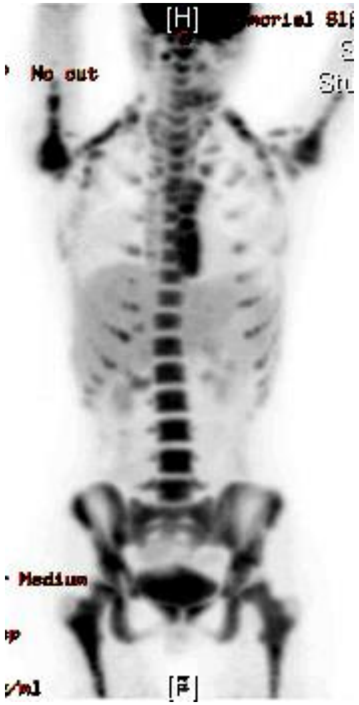

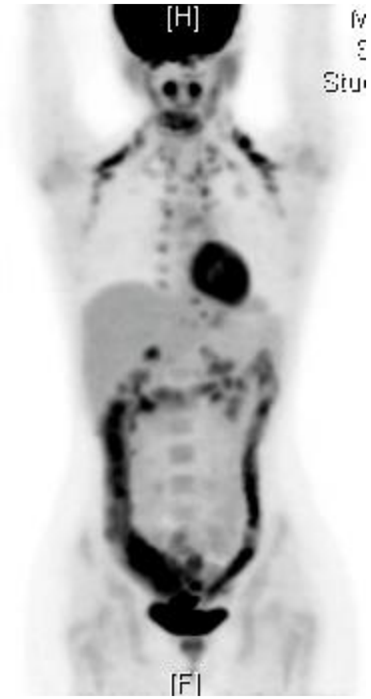

MRN	Age	IPS	Escalated	Cycles	SUV Ratio	Deauville	SUV	Liver	Response	RT	RT resp	Relapse	PFS	Death	OS	Rel prev site	Bx confirmed
	32	2-3	ABVD	6	>=1.3	DV-1-3	3.6	2.8	CR	0		0	32	0	32		

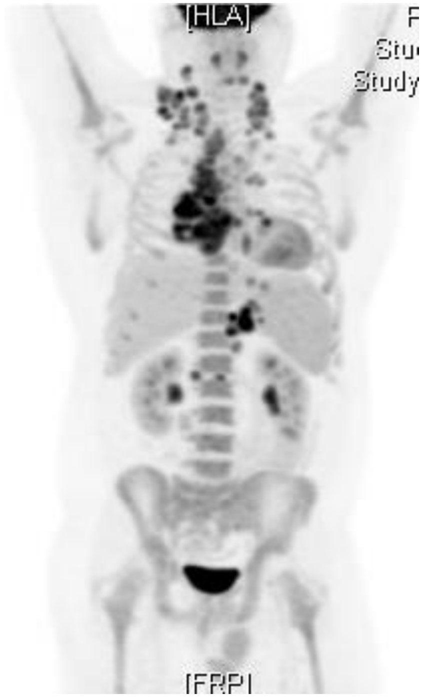

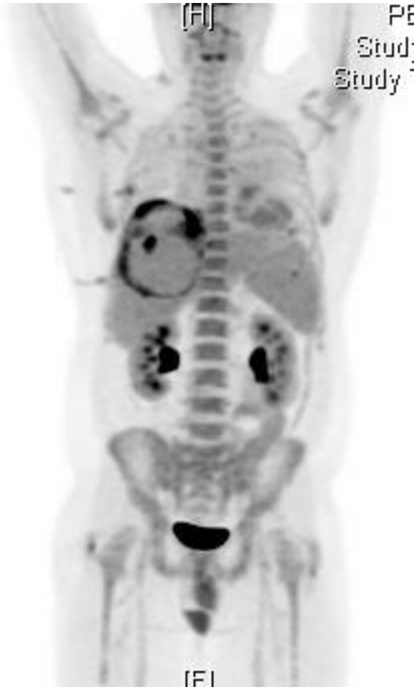

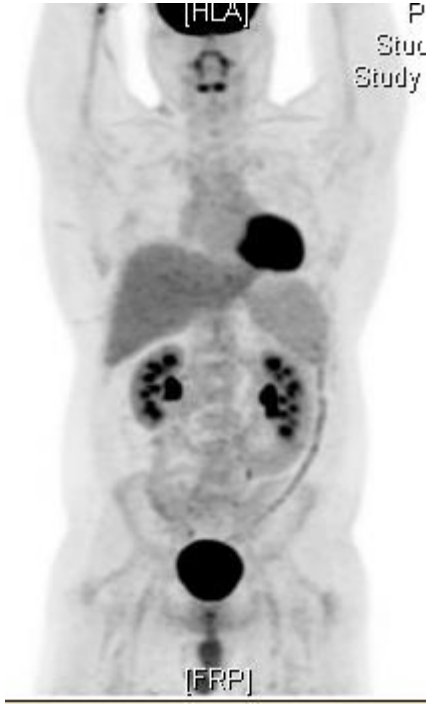

MRN	Age	IPS	Escalated	Cycles	SUV Ratio	Deauville	SUV	Liver	Response	RT	RT resp	Relapse	PFS	Death	OS	Rel prev site	Bx confirmed
	35	2-3	ABVD	6	>=1.3	DV-1-3	2.5	1.9	CR	0		0	50	0	50		

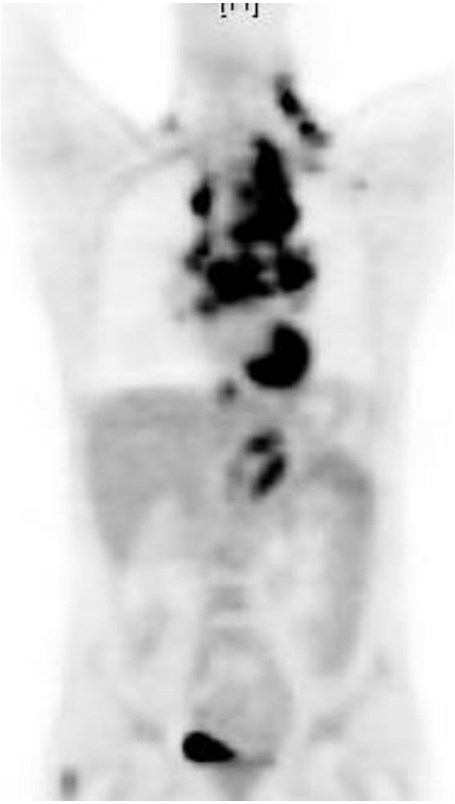

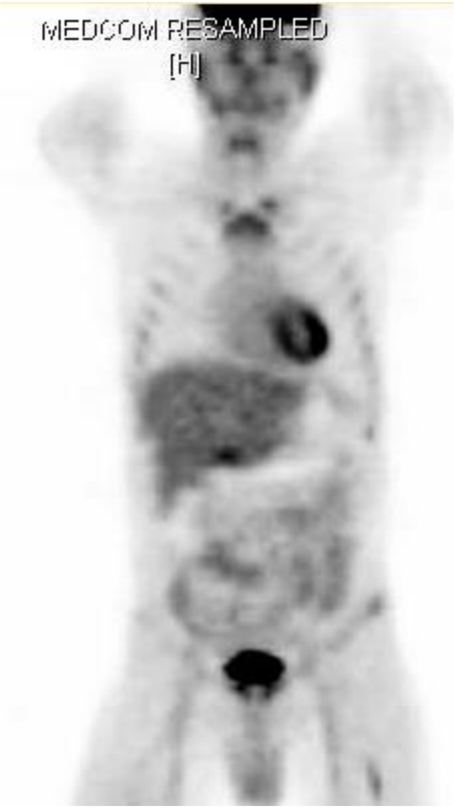

EOT - CT

MRN	Age	IPS	Escalated	Cycles	SUV Ratio	Deauville	SUV	Liver	Response	RT	RT resp	Relapse	PFS	Death	OS	Rel prev site	Bx confirmed
	47	4-7	ABVD	6	>=1.3	DV-1-3	2.3	1.6	CR	0		0	27	0	27		

OSH

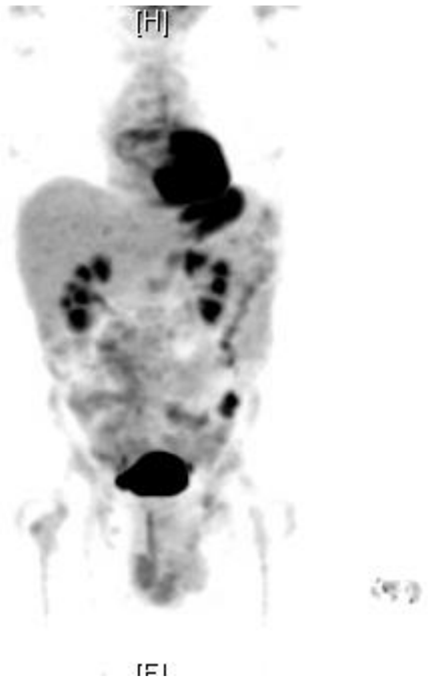

Only interim PET is available

OSH

MRN	Age	IPS	Escalated	Cycles	SUV Ratio	Deauville	SUV	Liver	Response	RT	RT resp	Relapse	PFS	Death	OS	Rel prev site	Bx confirmed
	19	0-1	ABVD	6	>=1.3	DV-1-3	4.4	1.8	CR	0		0	66	0	66		

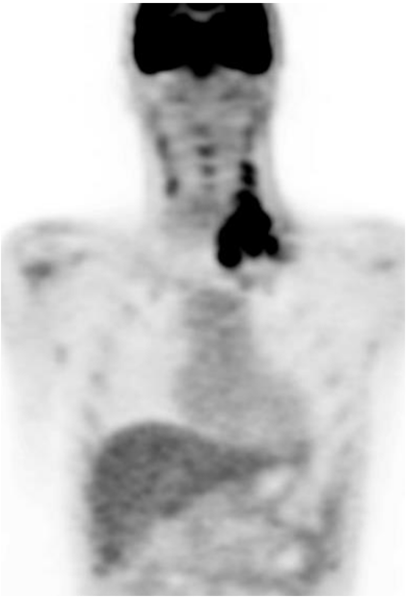

+ splenic lesion not visible on this section

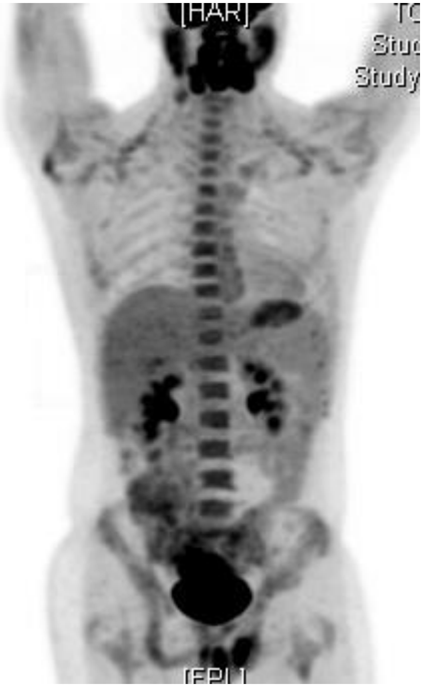

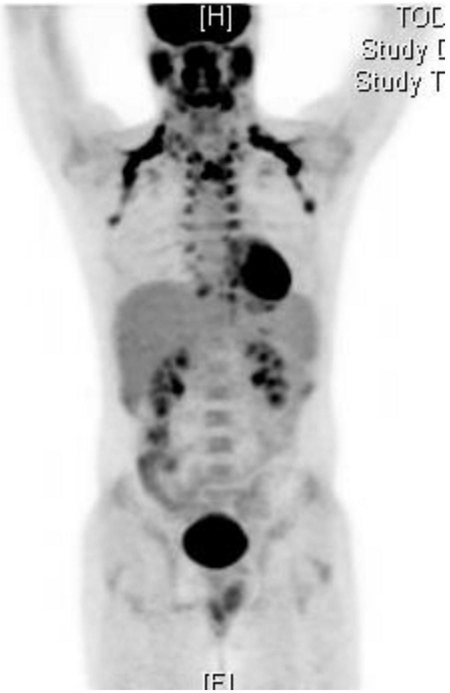

MRN	Age	IPS	Escalated	Cycles	SUV Ratio	Deauville	SUV	Liver	Response	RT	RT resp	Relapse	PFS	Death	OS	Rel prev site	Bx confirmed
	36	4-7	ABVD	6	>=1.3	DV-1-3	1.4	2	CR	0		0	66	0	66		

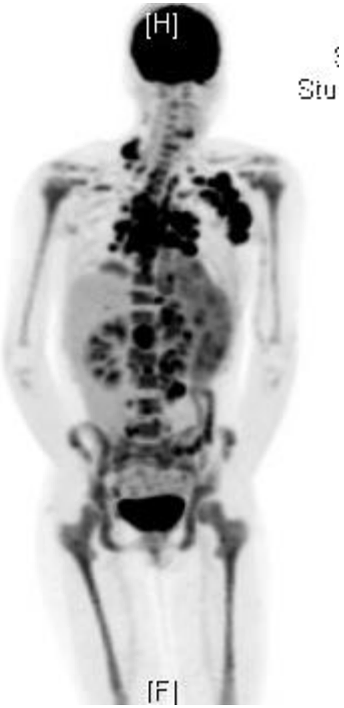

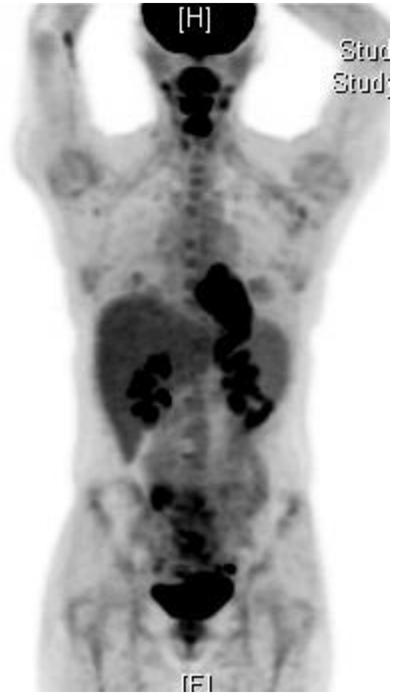

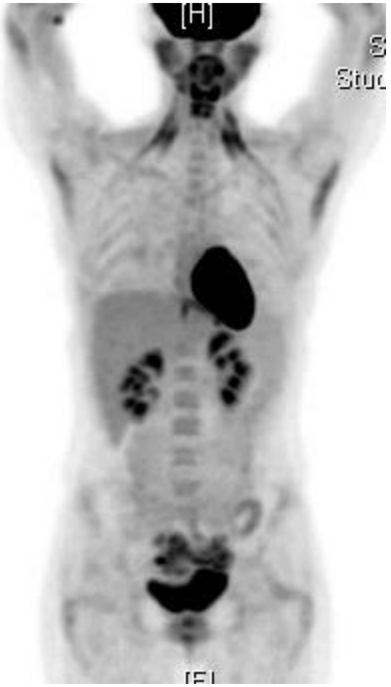

MRN	Age	IPS	Escalated	Cycles	SUV Ratio	Deauville	SUV	Liver	Response	RT	RT resp	Relapse	PFS	Death	OS	Rel prev site	Bx confirmed
	27	2-3	ABVD	6	>=1.3	DV-1-3	2.8	2	CR	0		0	38	0	38		

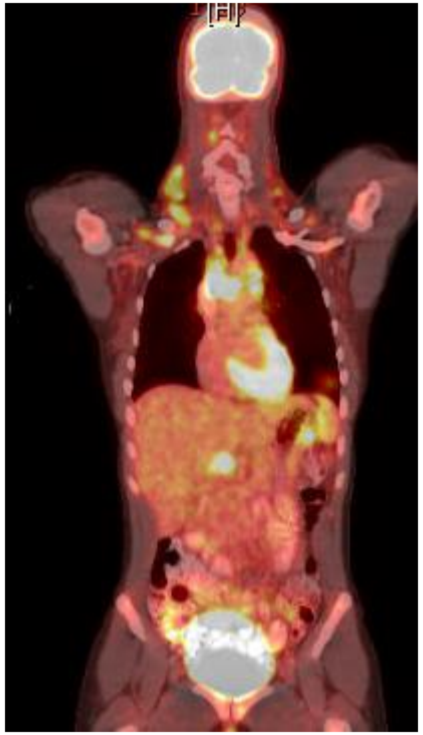
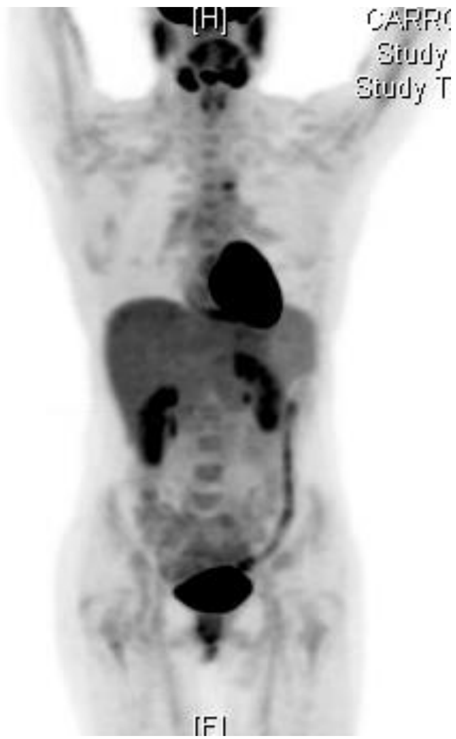

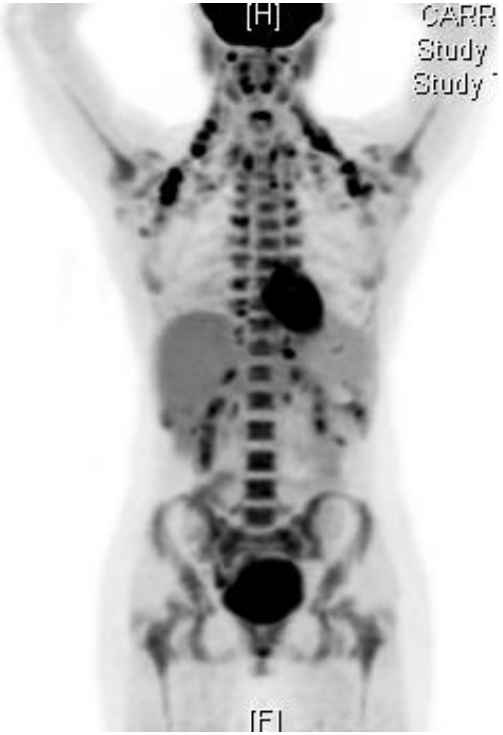

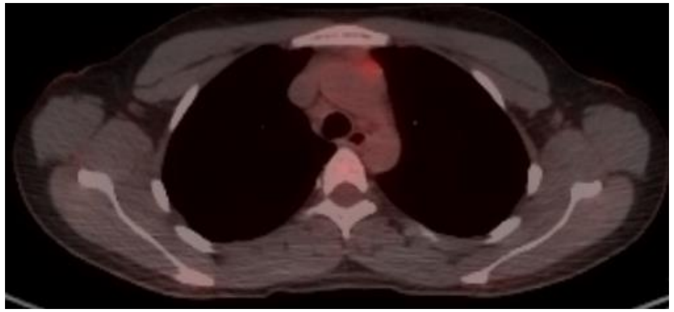

MRN	Age	IPS	Escalated	Cycles	SUV Ratio	Deauville	SUV	Liver	Response	RT	RT resp	Relapse	PFS	Death	OS	Rel prev site	Bx confirmed
	32	2-3	ABVD	6	>=1.3	DV-1-3	3.1	2.4	CR	0		0	32	0	32		

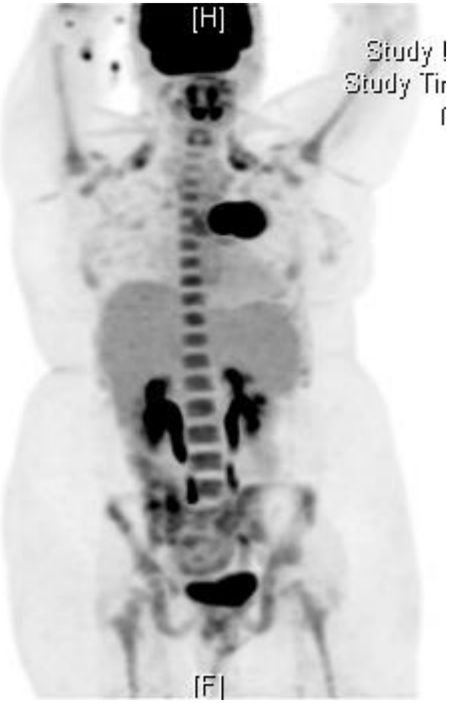

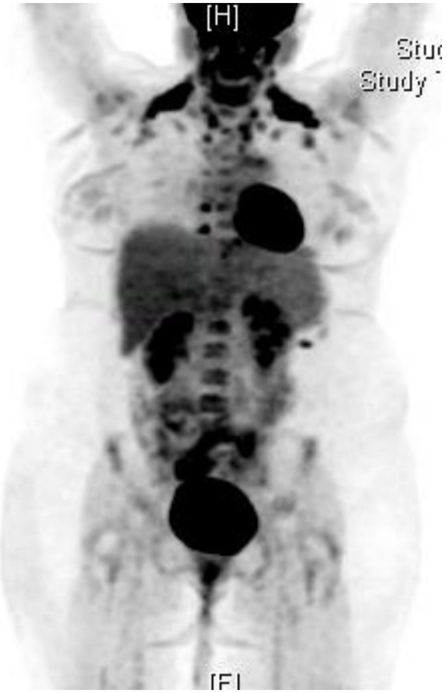

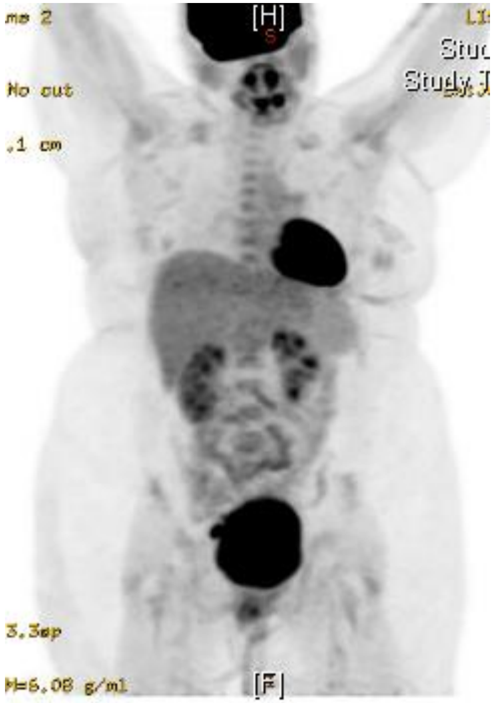

MRN	Age	IPS	Escalated	Cycles	SUV Ratio	Deauville	SUV	Liver	Response	RT	RT resp	Relapse	PFS	Death	OS	Rel prev site	Bx confirmed
	59	2-3	ABVD	6	>=1.3	DV-1-3	9.6	3.1	CR	0		0	81	0	81		

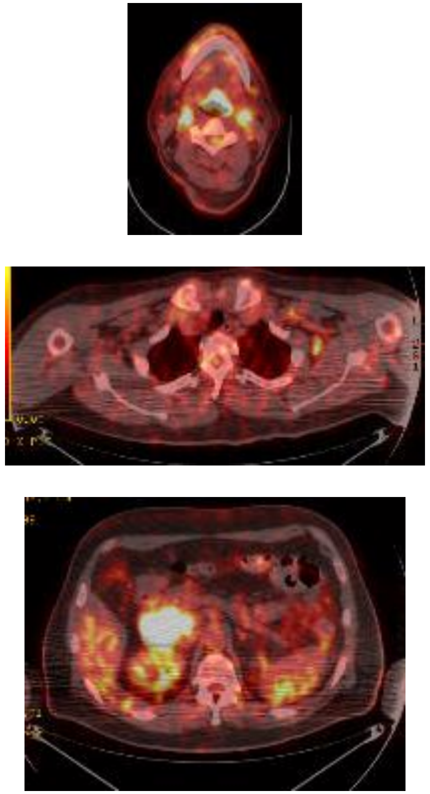

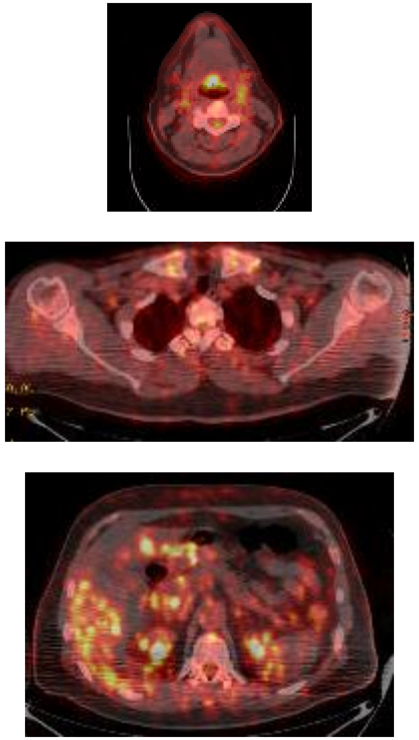

portocaval mass anterior to the right kidney 5.7cm SUVmax 9.6 aortocaval lymph node, which has increased SUV of 7.1

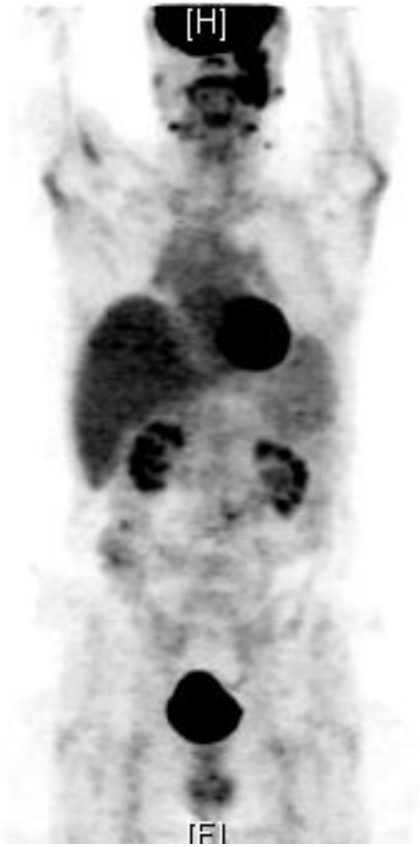

Hypermetabolic left masseter and pterygoid muscles SUV 6.9. All sites of initial disease resolved

MRN	Age	IPS	Escalated	Cycles	SUV Ratio	Deauville	SUV	Liver	Response	RT	RT resp	Relapse	PFS	Death	OS	Rel prev site	Bx confirmed
	36	4-7	ABVD	6	>=1.3	DV-1-3	3.6	2.2	CR	1		0	42	0	42		

OSH

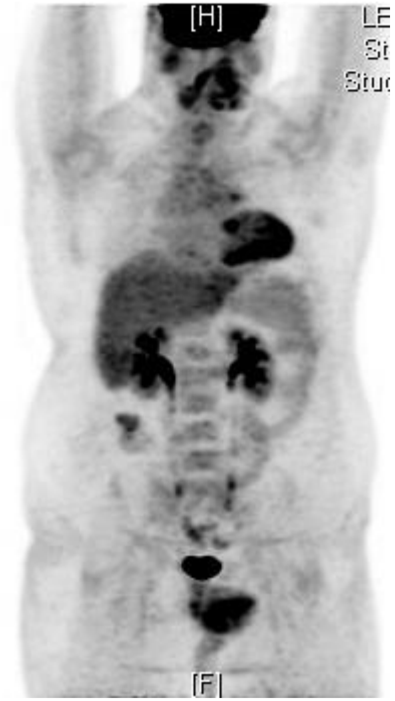

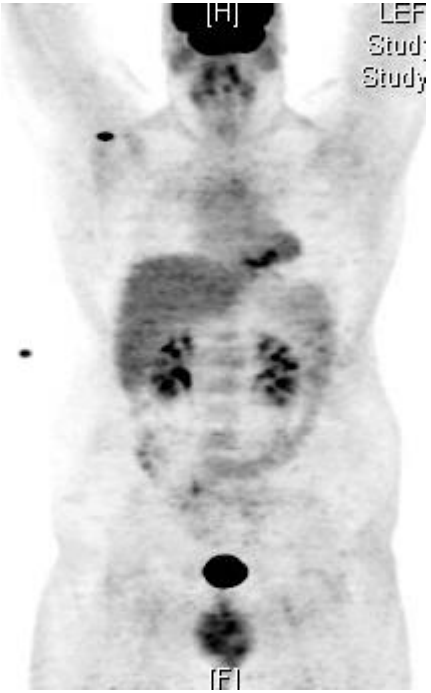

MRN	Age	IPS	Escalated	Cycles	SUV Ratio	Deauville	SUV	Liver	Response	RT	RT resp	Relapse	PFS	Death	OS	Rel prev site	Bx confirmed
	44	2-3	ABVD	6	>=1.3	DV-1-3	3.9	1.9	PR	1	CR	0	23	0	23		

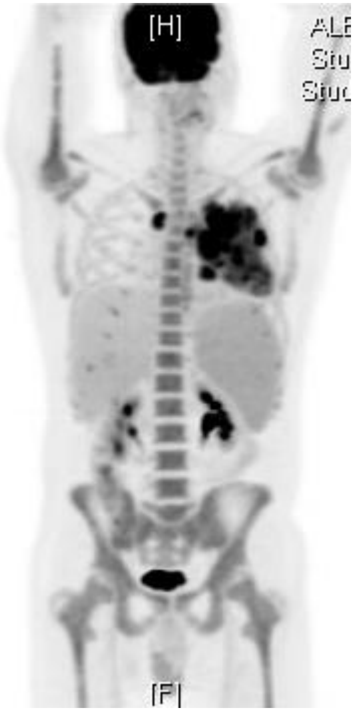

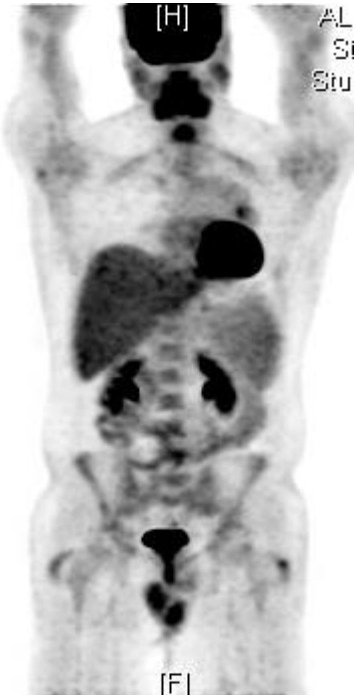

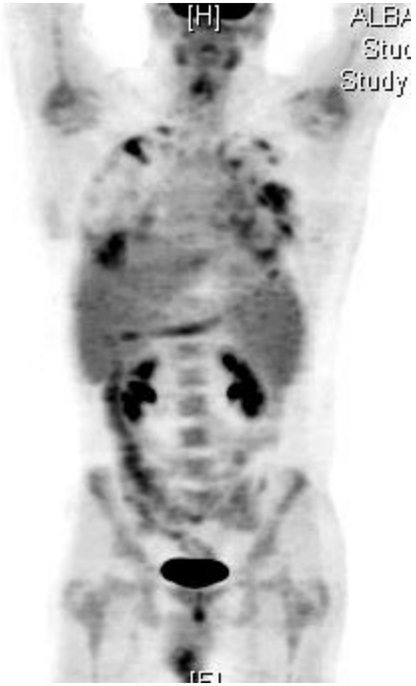

MRN	Age	IPS	Escalated	Cycles	SUV Ratio	Deauville	SUV	Liver	Response	RT	RT resp	Relapse	PFS	Death	OS	Rel prev site	Bx confirmed
	24	0-1	ABVD	5	>=1.3	DV-1-3	3	1.9	CR	1	CR	0	68	0	68		

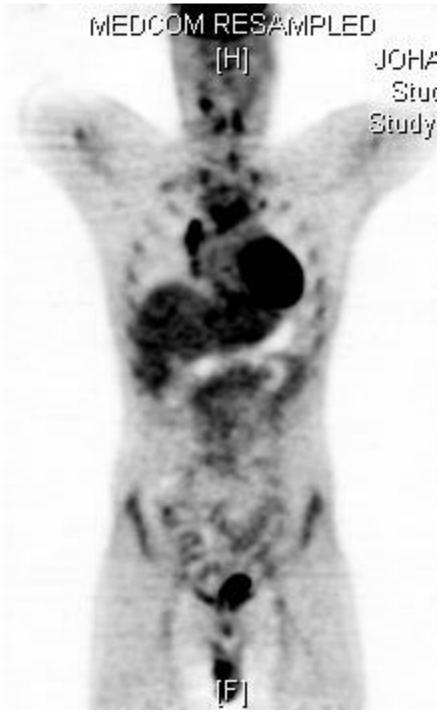

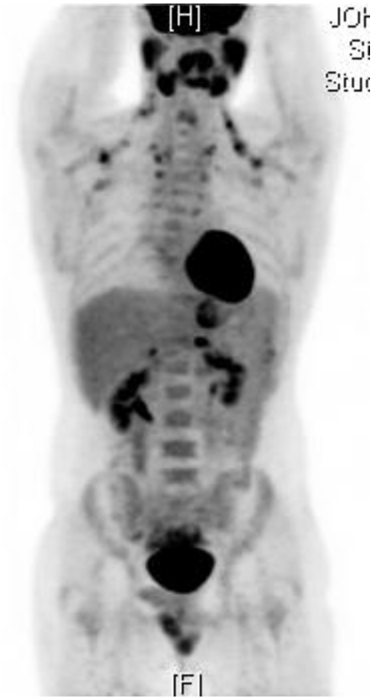

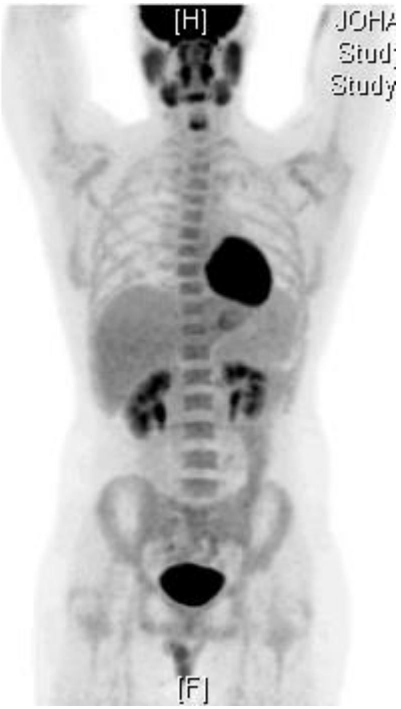

MRN	Age	IPS	Escalated	Cycles	SUV Ratio	Deauville	SUV	Liver	Response	RT	RT resp	Relapse	PFS	Death	OS	Rel prev site	Bx confirmed
	19	2-3	ABVD	6	>=1.3	DV-1-3	2.8	1.9	CR	0		0	40	0	40		

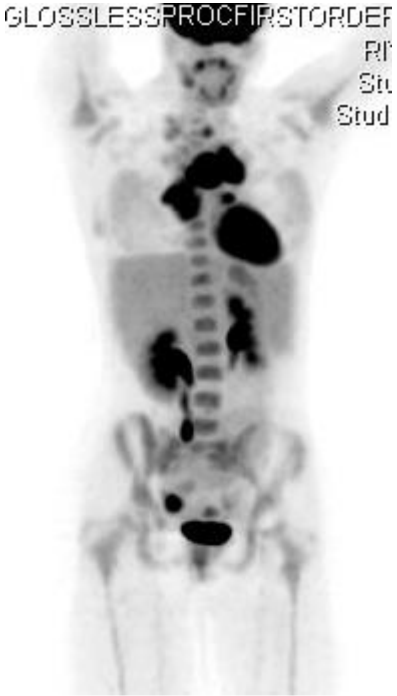

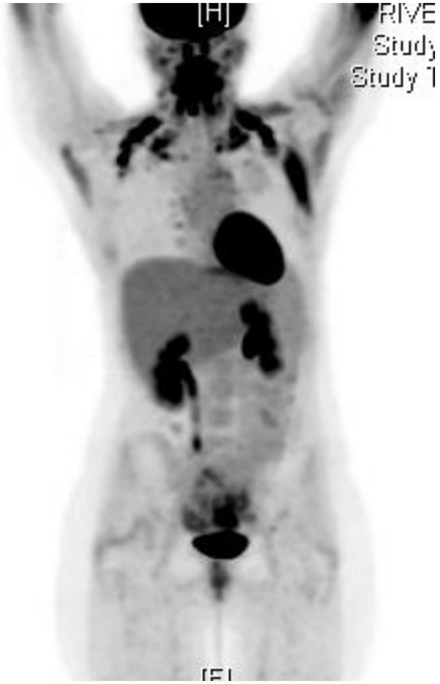

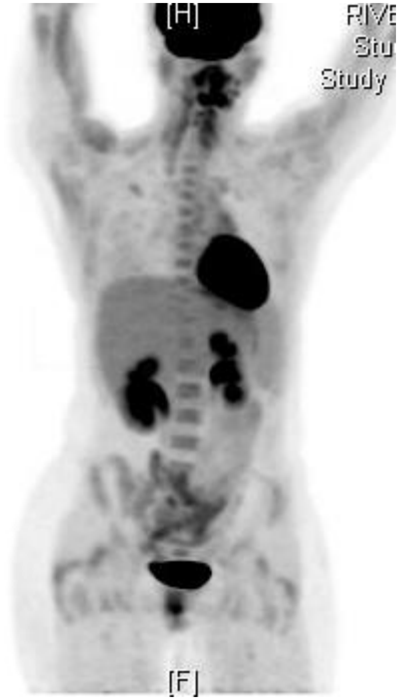

MRN	Age	IPS	Escalated	Cycles	SUV Ratio	Deauville	SUV	Liver	Response	RT	RT resp	Relapse	PFS	Death	OS	Rel prev site	Bx confirmed
	59	4-7	ABVD	6	>=1.3	DV-1-3	3	2.1	CR	0		0	9	0	9		

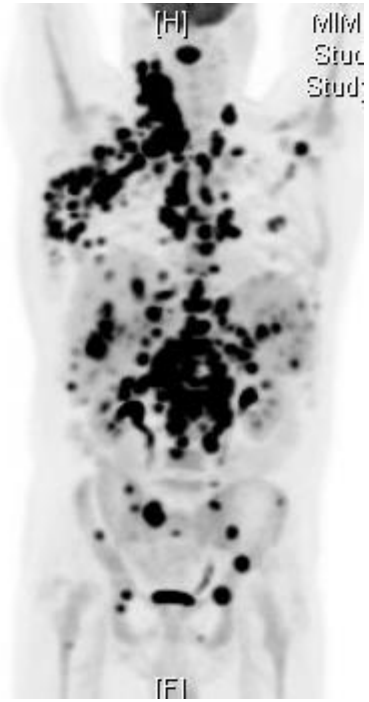

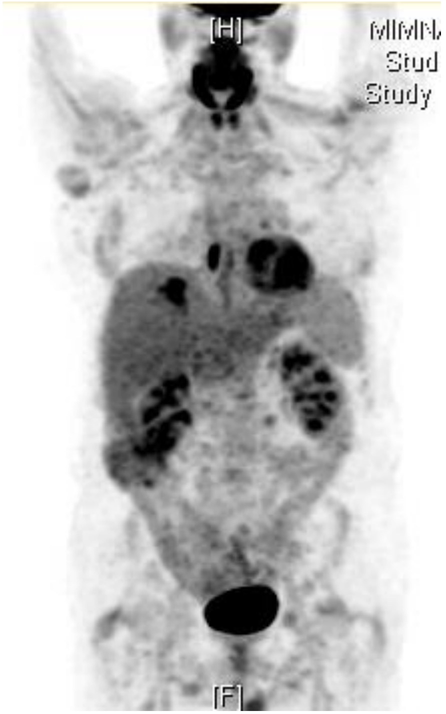

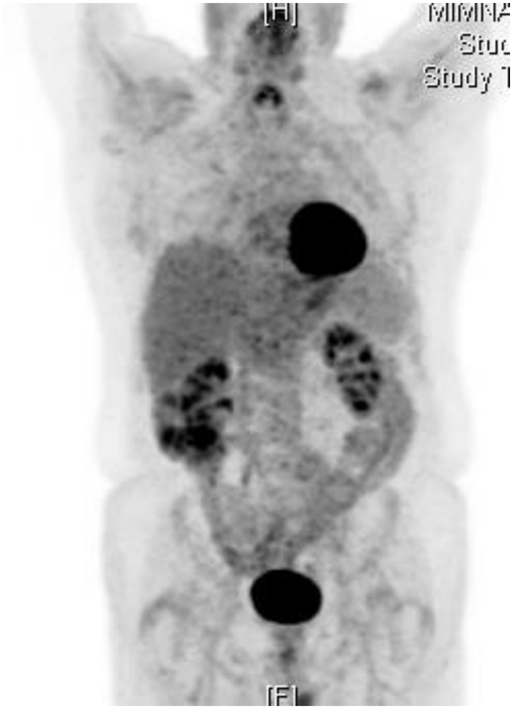

Supplement: Supplementary file 1 [file cancers-15-01760-s001.zip › cancers-2180835-supplementary/supplementary PET2 ABVD.pdf]
