# Supplementary material for: Outcomes of Patients with Positive Interim Positron Emission Tomography (PET) Continuing ABVD in the Clinical Setting
Source: Cancers (Basel). 2023 Mar 14;15(6):1760. doi: 10.3390/cancers15061760 (PMC10046293; doi:10.3390/cancers15061760)
Supplement: Supplementary file 1 [file cancers-15-01760-s001.zip › cancers-2180835-supplementary/Supplementary table S2.pdf]

Supplementary Table S2: Descriptive statistics by dvPET2 status

Characteristic	All	dvPET2 -	dvPET2 +	escBEACOPP†	*p**
No. of patients	227	170	48	9	
Median age (range), years	34 [18;87]	34.0 [18.0;87.0]	37.0 [19.0;76.0]	32.0 [19;59]	0.26
Age ≥ 45	57 (25%)	46 (23.6%)	8 (34.8%)	3 (33%)	0.36
Female sex	115 (51%)	94 (48.2%)	17 (73.9%)	4 (44%)	0.04
ECOG > 1	6 (3%)	5 (2.56%)	1 (4.35%)	0 (0%)	0.49
Ann Arbor Stage					0.04
II-U	52 (23%)	42 (21.5%)	7 (30.4%)	3 (33%)	
II-X	12 (5%)	9 (4.62%)	2 (8.70%)	1 (11%)	
III	83 (37%)	78 (40.0%)	3 (13.0%)	2 (22%)	
IV	80 (35%)	66 (33.8%)	11 (47.8%)	3 (33%)	
IPS score					0.02
0-3	201 (89%)	178 (91.3%)	17 (73.9%)	6 (67%)	
4-7	26 (12%)	17 (8.72%)	6 (26.1%)	3 (33%)	
Extranodal Involvement	87 (38%)	72 (36.9%)	12 (52.2%)	3 (33%)	0.23
Response (chemotherapy)					
CR	194 (86%)	176 (90.3%)	14 (60.9%)	4 (44%)	<0.001
PR	12 (5%)	5 (2.56%)	5 (21.7%)	2 (22%)	
POD	19 (8%)	13 (6.67%)	3 (13.0%)	3 (33%)	
TRM	2 (1%)	1 (0.51%)	1 (4.35%)	0 (0%)	
Radiotherapy	21 (9%)	14 (7.18%)	3 (13.0%)	4 (44%)	0.4

ECOG, Eastern Cooperative Oncology Group Performance Status; escBEACOPP, escalated bleomycin, etoposide, doxorubicin, cyclophosphamide, vincristine, procarbazine, prednisolone; IPS, International Prognostic Score; PET2, ^{18}F-fluoro-deoxy-D-glucose positron emission tomography performed after two cycles of adriamycin, bleomycin, vinblastine and dacarbazine. Data presented as N (%) for discrete rates and median [IQR] for numeric values. *p-values were calculated for the comparison ABVD PET2 positive vs. PET2 negative only. †Data about BEACOPP are presented for reference.
